# The Right Strap: The Role of Tether Position and Length in Acene Distortion

**DOI:** 10.1021/acs.orglett.5c02906

**Published:** 2025-09-11

**Authors:** Israa Shioukhi, Abhijeet Agrawal, Yinon Deree, Benny Bogoslavsky, Ori Gidron

**Affiliations:** Institute of Chemistry and Center for Nanoscience and Nanotechnology, 26742 The Hebrew University, Jerusalem 9190401, Israel

## Abstract

We report how the tether position affects the conformation, rigidity, and (chiro)­optical properties in anthracene derivatives. While 1,5- and 1,10-tethers (long and short diagonal tethering, respectively) induce comparable twisting, short diagonal tethered derivatives display greater conformational flexibility, significantly impacting fluorescence and chiroptical behavior. Notably, short and long diagonal octyl-tethered anthracenes exhibit inverse temperature-dependent CD responses attributed to thermally accessible twisted conformers. These findings highlight the critical role of the tether position in the design of functional curved aromatics.

## Graphical abstract

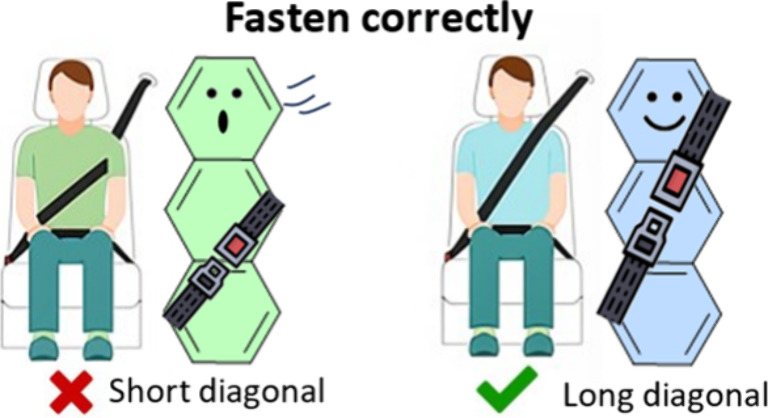

Distorting polyaromatic molecules out of planarity alters their electronic, optical, and chiroptical properties.^1,2^ For example, twisted acenes, commonly known as twistacenes, exhibit distinct properties compared to their planar counterparts, with applications in devices such as organic light-emitting diodes (OLEDs).^3,4^ One of the most effective strategies for inducing twisting in acenes is the tether-to-twist approach, which utilizes a diagonal tether of varying lengths.^5–8^ We have previously demonstrated that anthracene molecules diagonally tethered at positions 1 and 5 with different tether lengths (termed “long diagonal” or **1-C*n***, where C*n* designates the tether length, C8 for octyl, C4 for butyl, and C0 for no tether (Figure 1a)) allow systematic modulation of the twist angle (φ (Figure 1)) and rigidity.^9^ Consequently, the corresponding electronic, optical, and chiroptical properties, and even the chemical reactivity, are affected by tether length.^10,11^ For instance, an increased backbone twist correlates with a decrease in the HOMO–LUMO gap, an increased intersystem crossing rate, and enhanced molar ellipticity and circularly polarized luminescence (CPL).^12,13^ Tethering also results in increased rigidity, as a shorter tether increases the strain energy (the energy required to distort the backbone from equilibrium), resulting in a more rigid backbone.^9,14^

**Figure 1:**
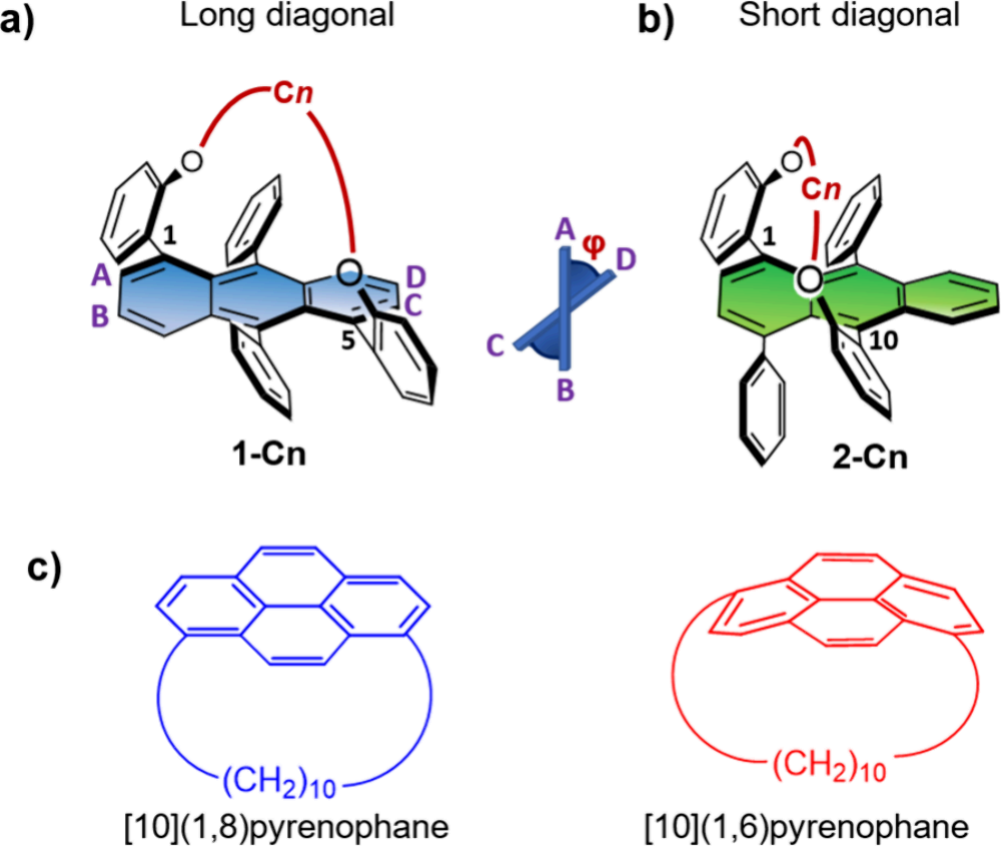
Structures of (a) **1-C*n*** and (b) **2-C*n***, tethered at the 1,5- and 1,10-positions of anthracenes, respectively. The A–B–C–D dihedral angle (φ) represents the acene twist. C*n* accounts for the tether length (C4, butyl; C8, octyl; C0, no tether). (c) [10]­(1,8)­pyrenophane and [10]­(1,6)­pyrenophane.^15^

In addition to variation of the tether length, positioning of the tether along the backbone can also affect the conformation and rigidity. For example, Bodwell demonstrated that for two pyranophanes consisting of a decyl tether in different positions, the pyrene backbone is more distorted in [10]­(1,6)­pyrenophane than in [10]­(1,8)­pyrenophane (Figure 1c).^15^ We recently reported isobenzofuranophane as a new synthon for inducing helicity in a quantitative manner.^16^ As the resulting tether is diagonally connected between two adjacent rings at positions 1 and 10 (termed “short diagonal” or **2-C*n*** (Figure 1b)), this new synthetic pathway enables us to examine the effects of both substitution patterns and tether lengths on backbone twisting and conformational rigidity and, in turn, on their optical and chiroptical properties.

Here, we present a comparative study of regioisomers of tethered twistacenes, specifically the 1,5- and 1,10-substituted derivatives, each synthesized with varying alkyl tether lengths (either butyl or octyl), as well as their nontethered analogues. Through X-ray crystallography, photophysical, and chiroptical characterizations, we investigate how substitution patterns and tether length affect the degree of twist, conformational rigidity, and chiroptical behavior.

The synthetic routes for **1-C*n*** and **2-C*n*** are described in Scheme 1. While we have previously reported the 1,5-tethered anthracenes,^9^ we present here an improved synthetic strategy by avoiding the use of a methoxy protecting group as well as the lithiation step, which reduces the number of steps from four to two and improves the overall yield from 1% to 12%. In addition, this allowed us to avoid the use of phenyls with electron-withdrawing groups at the 9,10-positions. The 1,5-substituted twistacenes were synthesized by coupling 1,5-dichloro-9,10-diphenylanthracene with 2-hydroxyphenyl boronic acid, followed by Williamson ether synthesis using either 1,4-dibromobutane or 1,8-dibromooctane to yield **1-C4** or **1-C8**, respectively (Scheme 1a). Nontethered derivative **1-C0** was synthesized via an analogous route by coupling phenylboronic acid (see the Supporting Information).

**Scheme 1:**
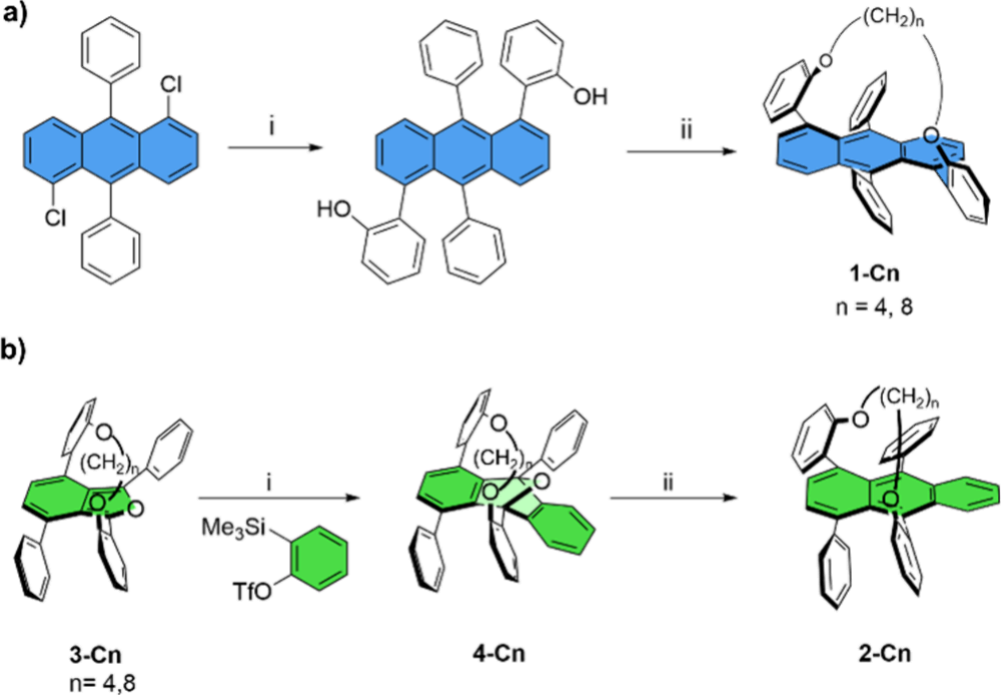
Syntheses of (a) **1-C*n*** ^a^ and **2-C*n*** ^b^

a Reagents and conditions: (i) 2-(hydroxy)­phenylboronic acid, Na_2_CO_3_, Pd­(PPh_3_)_4_, dioxane/water (4:1), 100 °C, 24 h, 35% yield; (ii) Br-(CH_2_)_*n*_-Br, K_2_CO_3_, DMF, 40–60 °C, *n* = 4 or 8 (28% or 33% yield, respectively).
b Reagents and conditions: (i) CsF, trifluoromethanesulfonate, acetonitrile/DCM (1:4), rt, 12 h, *n* = 4 or 8 (43% or 50% yield, respectively); (ii) NaI, trimethylsilyl chloride, 0 °C, 30 min, *n* = 4 or 8 (34% or 41% yield, respectively).

**2-C*n*** were synthesized via Diels–Alder cycloaddition between an isobenzofuranophene synthon **3-C4** and **3-C8**, with butyl or octyl tethers, respectively, and 2-(trimethylsilyl)­phenyl triflate, in the presence of cesium fluoride, which afforded adducts **4-C4** and **4-C8**, respectively (Scheme 1b).^16^ Subsequent deoxygenation using trimethylsilyl chloride and sodium iodide afforded the desired 1,10-substituted twistacene derivatives **2-C4** and **2-C8**. The nontethered derivative, **2-C0**, was synthesized in a similar manner starting from tetraphenyl-isobenzofuran (see the Supporting Information). All synthesized compounds were characterized by using NMR spectroscopy, mass spectrometry, and X-ray analysis. Single crystals were obtained through the slow evaporation of DCM/hexane mixtures.

The X-ray structures of **2-C*n*** and **1-C*n*** display a similar effect of the tether length on the backbone twisting, regardless of the substitution pattern (Figure 2). Both butyl-tethered twistacenes display significant end-to-end twist, with values of 31.9° for **2-C4** and a slightly larger twist of 33.8° for **1-C4**. In contrast, both **1-C8** and **2-C8** are nearly planar, with twists of 4.4° and 2.8°, respectively. Nontethered **2-C0** is nearly as twisted as butyl analogue **2-C4**, with 29.9° twisting, as a result of the steric repulsion between the peri-phenyl substituents. This demonstrates that the tether not only induces twisting (for the case of the butyl tether) but also can induce planarization depending on its length (such as for the case of octyl analogues **1-C8** and **2-C8**).

**Figure 2:**
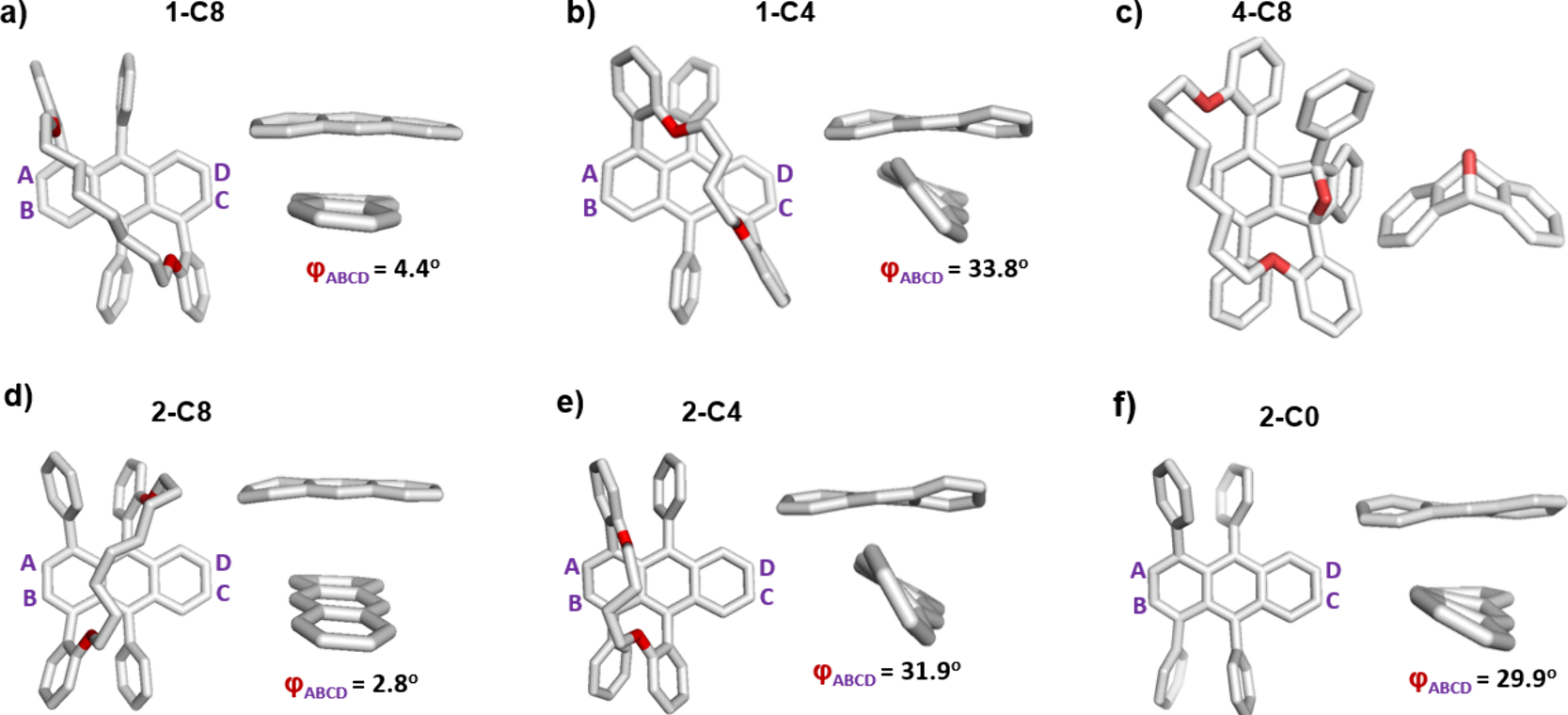
X-ray structures of (a) **1-C8**, (b) **1-C4**, (c) **4-C8**, (d) **2-C8**, (e) **2-C4**, and (f) **2-C0**. Hydrogens have been omitted for the sake of clarity. The anthracene core for each structure is depicted to the right of each X-ray structure, with the substituents removed for the sake of clarity.

The UV–vis absorption and fluorescence spectra are shown in Figure 3. All compounds display the characteristic anthracene absorption *p* band (S_0_ → S_1_ transition), which is essentially a π–π* HOMO–LUMO transition. A bathochromic shift of 0.1 eV is observed for both **1-C*n*** and **2-C*n*** upon twisting (from C8 to C4), explained previously by the increase in HOMO energy and decrease in LUMO energy levels, resulting in a smaller gap.^13^ The vibronic shoulders are broader in **2-C8** than in **1-C8**, indicating that the latter is more conformationally stable in the ground state. Additionally, nontethered analogue **1-C0** shows a featureless S_0_ → S_1_ transition, in contrast to **2-C0**. This suggests that both tethering and substitution patterning influence the rigidity, which can be attributed to the free rotation of the phenyl substituents.

**Figure 3:**
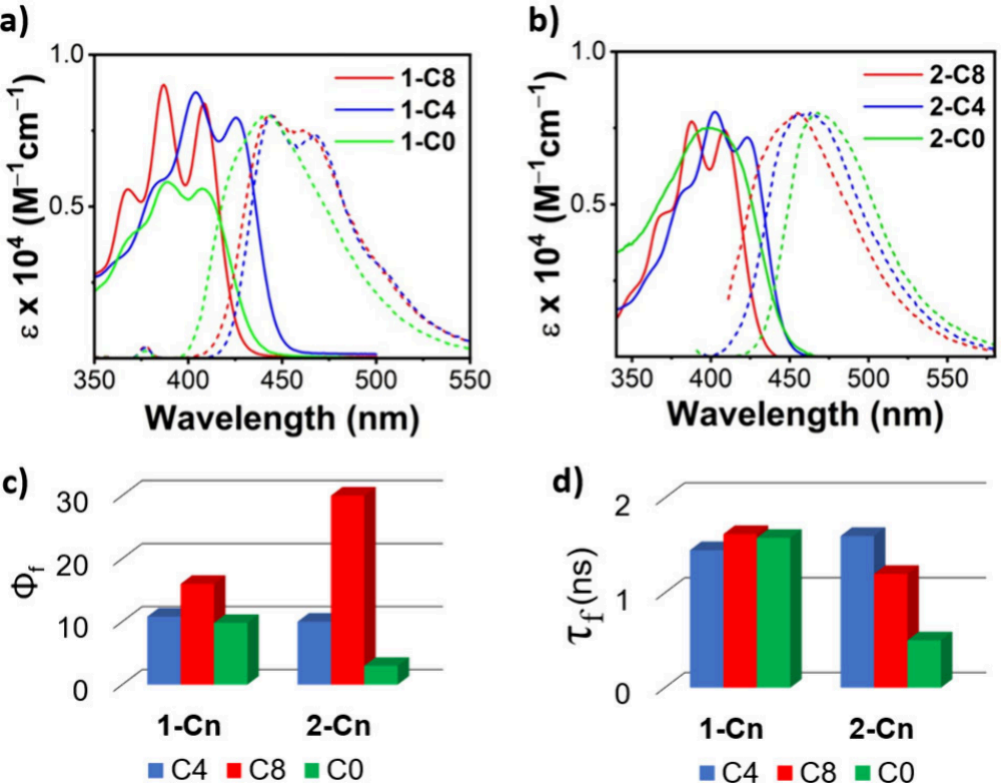
UV–vis absorption (solid line) and fluorescence (dashed line) spectra (a) **1-C*n*** and (b) **2-C*n***. (c) Fluorescence quantum efficiencies and (d) fluorescence lifetimes. Measured in hexane.

The increased rigidity of **1-C*n*** is also expressed in their emission spectra, with a clear vibronic shoulder for **1-C4** and a slightly broader vibration for **1-C8**. In contrast, all of the **2-C*n*** compounds display featureless emission spectra regardless of the tether lengths (Figure 3a,b). The fluorescence quantum efficiency, Φ_f_, decreases with an increase in backbone twist. For **1-C*n***, Φ_f_ decreases from 16% for **1-C8** to 10% for **1-C4**, and a similar Φ_f_ decreases for **1-C0**. In comparison, the Φ_f_ of **2-C*n*** is more strongly dependent on tether length, ranging from 30% for **2-C8** to 10% for **2-C4**, and from only 3% for **2-C0** (Figure 3c). The reduction of Φ_f_ with an increase in twist was previously ascribed to an increase in the intersystem crossing as the deviation from planarity increases.^17^ The fluorescence lifetime (τ_f_) follows a similar trend, with significant dependency on the bridge length for **2-C*n*** and only a weak dependency for **1-C*n*** (Figure 3d). Overall, the short diagonal (**2-C*n***) tethering results in a larger variability of the excited-state geometry, depending on the tether length, as indicated by the fluorescence spectra, quantum yields, and lifetimes. These differences can stem from the greater conformational flexibility of short tethered **2-C*n*** compared with long tethered **1-C*n***.

All tethered twistacenes were separated into their *P* and *M* enantiomers using chiral HPLC, and the ECD spectra are displayed in Figure 4. The absolute configuration (*P*/*M*) was determined by comparing the calculated and experimental ECD spectra (Figures S107 and S108). While the ECD increases significantly for shorter tethers and larger twisting angles, as was previously observed, the molar ellipticity values (Δε) for 1,5-substituted **1-C*n*** are overall higher compared with those of 1,10-substituted **2-C*n*** for the same *n*. This can be explained in part by the large degree of twisting of **1-C*n*** compared with that of **2-C*n***, but it can also be related to the induced chirality of the different substitution patterns.

**Figure 4:**
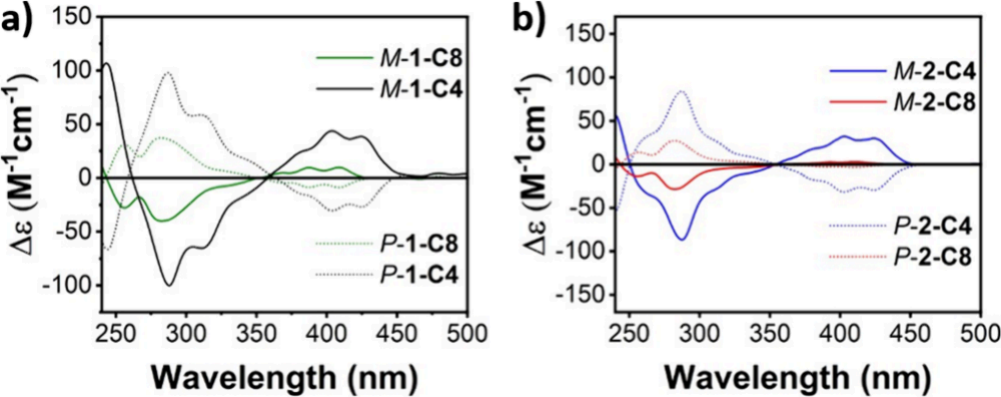
ECD spectra of (a) **1-C*n*** and (b) **2-C*n***. Measured in chloroform.

Since the ECD is highly sensitive to conformational changes, we were interested in studying variable-temperature ECD (VT-ECD) as a tool to assess conformational stability with short and long diagonal tethering orientations and lengths. In most cases, cooling leads to an increase in molar ellipticity, as it reduces the different degrees of freedom (e.g., phenyl rotations) and stabilizes the lowest-energy conformer.^18,19^ We were therefore surprised that for the longer octyl tether, **1-C8** and **2-C8** displayed opposing temperature dependencies. While for **1-C8**, |Δε| of the S_0_ → S_1_ transition increases with cooling (Figure 5a, blue line), as well as for higher-energy transitions, for **2-C8**, |Δε| decreases with cooling (Figure 5a, green line). To explain this unexpected behavior, we have explored the conformational space using GMMX to identify the possible conformers within the energy range of up to 5 kcal/mol, followed by a higher-level calculation (DFT/B3LYP/6-311G­(d)-D3) for the 15 lowest-energy conformers (see the Supporting Information for details). We found that for both **2-C8** and **1-C8**, the lowest-energy conformer is nearly planar (2.5° torsion). However, for **1-C8**, other conformers are not thermally accessible (>3 kcal/mol (Figure 5b, blue)) and, therefore, do not contribute significantly even at room temperature (population of <1%). Thus, the significant contribution is the restricted phenyl rotation in this specific conformer, resulting in the increase in |Δε|. In contrast, **2-C8** consists of twisted conformers, which are only 1 kcal/mol higher in energy than the lowest-energy conformer (Figure 5b, green line). These twisted conformers, which are expected to exhibit stronger |Δε| compared to the planar conformer, are abundant at room temperature but not at lower temperatures. For example, in a two-level system with a 1 kcal/mol difference, the population increases from 6% at 190 K to 15% at room temperature.^20^ This can explain the opposite trends observed for compounds **1-C8** and **2-C8**.

**Figure 5:**
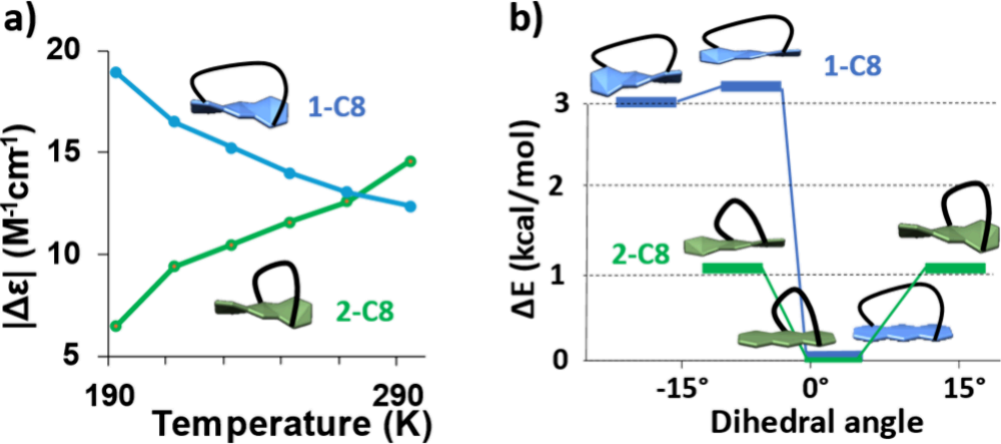
(a) VT-ECD spectra of **1-C8** (blue) and **2-C8** (green), following the maximal molar ellipticity of the lowest-energy transition (403 nm for **1-C8** and 412 nm for **2-C8**) in dichloromethane, showing opposite temperature dependence. (b) Three calculated lowest-energy conformers (B3LYP/6-311G­(d)-GD3) selected from a GMMX scan.

In conclusion, this work demonstrates that not only the tether length but also its position directly affects the optical and chiroptical properties in acenes. While long diagonal tethering (**1-C*n***) and short diagonal tethering (**2-C*n***) result in similar degrees of backbone twisting, their conformational stability varies significantly, with short diagonal tethering being more flexible. This flexibility is reflected in the stronger dependency on the tether length of the fluorescence quantum efficiencies, lifetimes, and molar ellipticities. In addition, anthracenes with long and short octyl tethers exhibit opposite temperature dependency of their CD spectra, which can be attributed to the contribution of twisted conformers for the **2-C*n*** at room temperature. In a broader perspective, these findings underscore the significance of the tether position as a factor in controlling the properties of curved aromatics.

## Supplementary Material



## Data Availability

The data underlying this study are available in the published article and its Supporting Information.

## Notes

Experimental procedures, spectral data, X-ray structural data, details of electrochemical measurements, and computational details (PDF)
