# Supplementary material for: The Right Strap: The Role of Tether Position and Length in Acene Distortion
Source: Org Lett. 2025 Sep 11;27(38):10642–6. doi: 10.1021/acs.orglett.5c02906 (PMC12481544; doi:10.1021/acs.orglett.5c02906)
Supplement: Supplementary file 1 [file ol5c02906_si_001.pdf]

Supporting Information

The Right Strap: The Role of Tether Position and Length in Acene Distortion

Israa Shioukhi, Abhijeet Agrawal, Yinon Deree, Benny Bogoslavsky, and Ori Gidron*

Institute of Chemistry and Center for Nanoscience and Nanotechnology, The Hebrew University, Jerusalem 9190401, Israel

Table of Contents

S1 General 4

S2 Synthesis 5

S2.1 Synthesis of **1-Cn** 5

S2.1.1 Synthesis of **1-C0** 5

S2.1.2 Synthesis of **6** 6

S2.1.3 Synthesis of **1-C4** 7

S2.1.4 Synthesis of **1-C8** 8

S2.2.1 Synthesis of **4-C0** 9

S2.2.2 Synthesis of **2-C0** 10

S2.2.3 Synthesis of **4-C4** 11

S2.2.4 Synthesis of **2-C4** 12

S2.2.5 Synthesis of **4-C8** 13

S2.2.6 Synthesis of **2-C8** 14

S3 Characterization........ 15

S3.1 NMR........ 15

S4 HPLC chiral separation........ 53

S4.1 **1-Cn** 53

S4.2 **2-Cn** 53

S5 Photophysical Properties 55

S5.1 UV-vis absorption spectra........ 55

S5.1.1 **1-Cn** 55

S5.1.2 **2-Cn** 56

S5.2 ECD spectra 57

S5.2.1 **1-Cn** 57

S5.2.2 **2-Cn** 58

S5.2.3 Dissymmetry factor (g_{abs}) of 1-Cn 58

S5.2.4 Dissymmetry factor (g_{abs}) of 2-Cn 59

S5.3 Fluorescence spectra 59

S5.4 Excitation spectra 60

S5.4.1 **1-Cn** 60

S5.4.2 **2-Cn** 62

S5.5 Fluorescence lifetime 64

S5.5.1 **1-Cn** 64

S5.5.2 **2-Cn** 64

S5.6 Fluorescence quantum yields 65

S6 Single-crystal X-ray diffraction crystallography (SCXRD). 66

S6.1 X-ray structures 66

S6.2 Crystals data 72

S6.2.1 **2-C4** 72
S6.2.2 **1-C4** 73
S6.2.3 **2-C8** 74
S6.2.4 **1-C8** 75
S6.2.5 **4-C8** 76
S6.2.6 **2-C0** 77
S7 Computational Details 78
S8 References 82

S1 General

Commercially available reagents and chemicals were used without further purification unless otherwise stated. Compounds **5** and **3-C0, 3-C4, 3-C8** were synthesized according to a previous reports.[1,2]

Flash chromatography (FC) was performed using CombiFlash SiO_2 columns. Chiral HPLC separations were performed with a Chiralpak® IG semi-preparative column and CHIRALPAK® IB-N (250 × 4.6 mm / 5μm) preparative columns, with hexane/dichloromethane as eluent.

^{1}H and ^{13}C NMR spectra were recorded in solution on a Bruker-AVIII 400 MHz and 500 MHz spectrometers using tetramethyl silane (TMS) as the external standard. The spectra were recorded using chloroform-*d*. Chemical shifts are expressed in δ units.

UV-vis absorption spectra were recorded with an Agilent Cary-5000 spectrophotometer. The spectra were measured using a quartz cuvette (1 cm) at 25 °C. The absorption wavelengths are reported in nm with the extinction coefficient ε ($M^{-1}cm^{-1}$) in brackets.

Steady state fluorescence measurements were performed on a HORIBA JOBIN YVON Fluoromax-4 spectrofluorometer with the excitation/emission geometry at right angles. Fluorescence quantum yields were determined using a standard procedure under a HORIBA F integrating sphere. The lifetimes of the excited species were measured using an NL-C2 Pulsed Diode Controller NanoLED light source with time-correlated single proton counting (TSCPC) Controller DeltaHub (HORIBA), referenced against colloidal Ludox solution (50 wt. % solution in water) obtained from Aldrich.

Electronic Circular Dichroism (ECD) spectra were recorded on a MOS-500 spectrophotometer from BioLogic Science Instruments.

High resolution mass spectra were measured on a HR Q-TOF LCMS and Waters Micromass GCT_Premier Mass Spectrometer using ESI.

A suitable crystal was selected and mounted on a suitable support on an XtaLAB Synergy, Single source at offset/far, HyPix diffractometer. The crystal was kept at a steady T = 200.0(1) K during data collection. The structure was solved with the ShelXT (Sheldrick, 2015) structure solution program using the Intrinsic Phasing solution method and by using Olex2 (Dolomanov et al., 2009) as the graphical interface. The model was refined with version 2018/3 of ShelXL 2018/3 (Sheldrick, 2015) using Least Squares minimization.

S2 Synthesis

S2.1 Synthesis of **1-Cn**

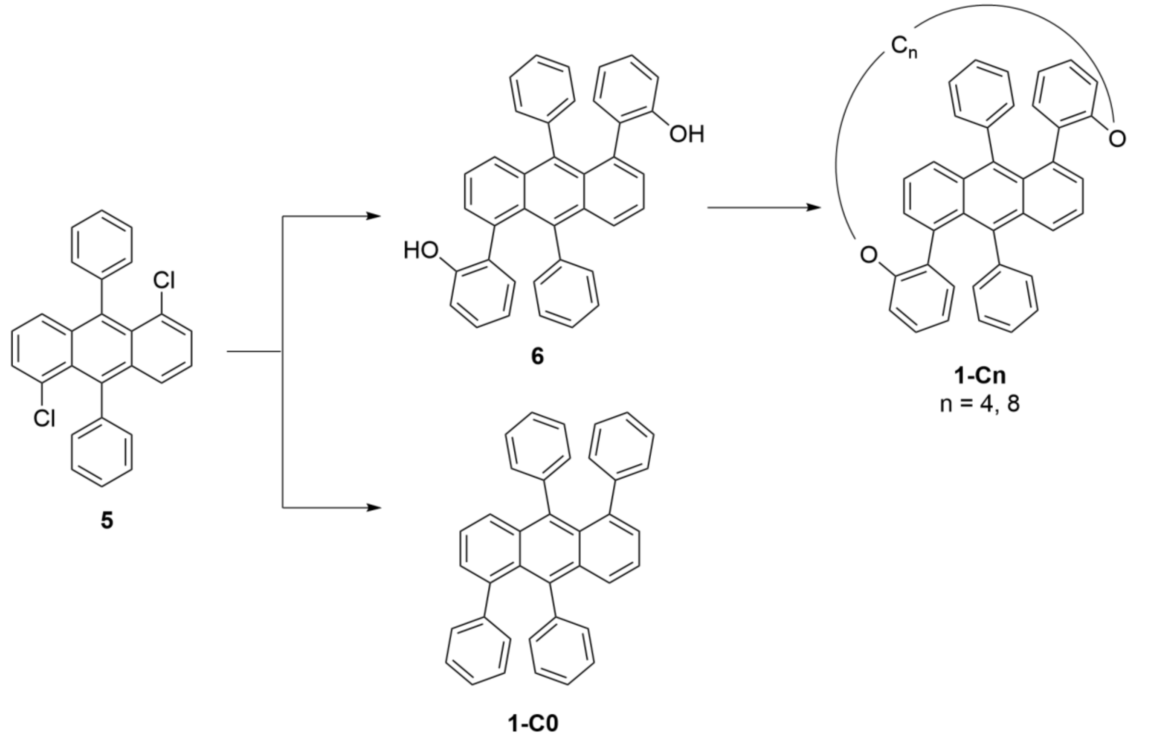

S2.1.1 Synthesis of **1-C0**

Compound **5** (100 mg, 0.625 mmol), phenylboronic acid (75 mg, 0.25 mmol), Na_2CO_3 (200 mg, 1.875 mmol) and Pd $(PPh_3)_4$ (10 mg, 0.0312 mmol) were dissolved in a mixture of 1,4-Dioxane (80 mL) and water (20 mL), followed by purging with argon for 20 min. The reaction mixture was stirred at 100 °C in an oil bath for 24 h. The mixture was evaporated and extracted with ethyl acetate (3 X 90 mL), dried over $MgSO_4$ and concentrated to obtain brown solid, which was purified of by silica gel column chromatography using hexane to afford **1-C0** as a yellow solid (27 mg, 29% yield).

^{1}H NMR (500 MHz, CDCl3) δ 7.67 (dd, J = 8.9, 1.2 Hz, 1H), 7.65 – 7.60 (m, 1H), 7.57 – 7.51 (m, 4H), 7.51 – 7.47 (m, 3H), 7.46 (dd, J = 7.1, 1.2 Hz, 1H), 7.31 (dd, J = 8.9, 6.6 Hz, 2H), 7.20 (dd, J = 6.6, 1.3 Hz, 1H), 7.09 (dd, J = 9.0, 7.1 Hz, 1H), 7.07 – 7.01 (m, 4H), 6.96 (ddd, J = 5.6, 2.7, 1.8 Hz, 6H), 6.89 – 6.84 (m, 2H).

^{13}C NMR (126 MHz, CDCl3) δ 144.4, 141.3 (d, J = 3.1 Hz), 141.1, 140.5 (d, J = 4.8 Hz), 138.7, 136.4, 133.1, 133, 132.6, 131.9, 131.6, 131.5, 130.4, 130.2, 130.1, 129.8, 129.5, 129, 128.6, 127.8, 127.5, 127.4, 127.2, 127.1, 127.1, 126.9, 126.7, 126.4, 125.6, 125.4, 125.2, 125.2, 124.7, 124.2, 124.

HR-ESI-MS m/z (%): 482.2018 for (100, $[M]^+$) calcd. for $C_{38}H_{26}^+$: 482.2035.

S2.1.2 Synthesis of **6**

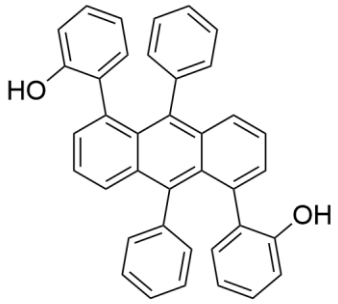

Compound **5** (1 g, 2.50 mmol), 2-(hydoxy) phenylboronic acid (0.863 g, 6.26 mmol), Na_2CO_3 (0.796 g, 7.51 mmol) and $Pd(PPh_3)_4$ (145 mg, 0.125 mmol) were dissolved in a mixture of 1,4-Dioxane (80 mL) and water (20 mL), followed by purging with argon for 20 min. The reaction mixture was stirred at 100 °C in an oil bath for 24 h. The mixture was evaporated and extracted with ethyl acetate (3 X 90 mL). dried over $MgSO_4$ and concentrated to obtain brown solid, which was purified by silica gel column chromatography using ethyl acetate/hexane (4:6) to afford compound **6** as a yellow solid (450 mg, 35% yield). the final product was obtained as *syn*-6 and *anti*-6 atropisomers.

^{1}H NMR (400 MHz, $CDCl_3$) δ 7.56 (ddd, *J* = 7.3, 3.5, 2.8 Hz, 2H), 7.31 – 7.26 (m, 4H), 7.24 – 7.05 (m, 9H), 7.03 – 6.96 (m, 2H), 6.93 (ddt, *J* = 8.2, 7.3, 1.6 Hz, 2H), 6.82 – 6.74 (m, 2H), 6.64 (tdd, *J* = 7.4, 2.7, 1.2 Hz, 2H), 6.53 – 6.48 (m, 2H), 4.70 (s, 1H), 4.61 (s, 1H).

^{13}C NMR (101 MHz, $CDCl_3$) δ 151.8, 151.7, 139.9, 139.7, 138.5, 134.6, 133, 132.9, 132.5, 132.4, 131.7, 131.6, 131.5, 131.4, 130.8, 130.7, 130.5, 129.6, 129.4, 128.6, 128.5, 128, 127.9, 127.6, 127.5, 127.3, 127.2, 124.9, 124.8, 120.2, 120.1, 115.4, 115.3.

HR-ESI-MS m/z (%): 514.1936 for (100, $[M]^+$) calcd. for $C_{38}H_{26}O_2^+$: 514.1934.

S2.1.3 Synthesis of **1-C4**

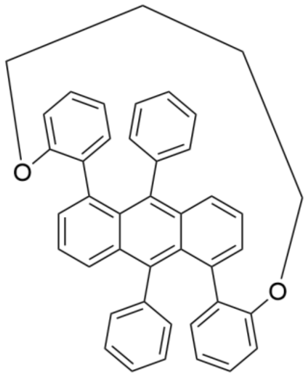

Compound **6** (200 mg, 0.388 mmol) and K_2CO_3 (161 mg, 1.17 mmol) were purged with argon, 20 mL of anhydrous N,N-DMF was added to the mixture. After stirring the solution for 10 min at rt, the formation of the dipotassium salt of **6** was confirmed by a change from clear to curdy solution. 1,4-dibromobutane (42 mg, 0.194 mmol) was added and reaction mixture heated to 60 °C in an oil bath for 48 h. The mixture was evaporated, and the crude was purified using silica gel column chromatography by ethyl acetate/hexane mixture (2:8) to afford **1-C4** as a yellow solid (60 mg, 28% yield).

^{1}H NMR (400 MHz, $CDCl_3$) δ 7.54 (dd, *J* = 8.8, 1.2 Hz, 2H), 7.25 – 7.23 (m, 1H), 7.17 (dd, *J* = 7.6, 1.5 Hz, 2H), 7.13 (ddd, *J* = 6.9, 4.9, 1.4 Hz, 4H), 7.10 – 7.03 (m, 6H), 7.00 (dd, *J* = 7.5, 1.4 Hz, 2H), 6.94 (td, *J* = 7.8, 1.7 Hz, 2H), 6.63 (td, *J* = 7.5, 1.0 Hz, 2H), 6.33 (dd, *J* = 8.1, 1.1 Hz, 2H), 3.73 (d, *J* = 8.8 Hz, 2H), 3.34 (t, *J* = 9.4 Hz, 2H), 1.00 – 0.86 (m, 4H).

^{13}C NMR (101 MHz, $CDCl_3$) δ 155.2, 140.5, 137.6, 136.9, 133.2, 133.1, 133, 131, 1302, 129.5, 128.8, 128, 127.6, 127.1, 127, 126, 124.3, 120.3, 109.2, 66.2, 26.8.

HR-ESI-MS m/z (%): 568.2392 for (100, $[M]^+$) calcd. for $C_{42}H_{32}O_2^+$: 568.2397.

S2.1.4 Synthesis of **1-C8**

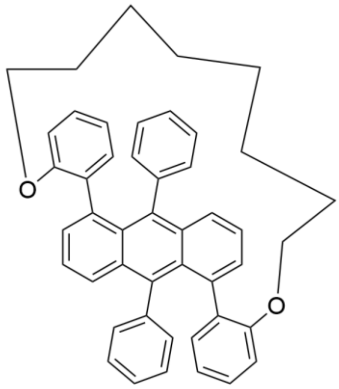

Compound **6** (200 mg, 0.388 mmol) and K_2CO_3 (161 mg, 1.17 mmol) were purged with argon, 20 mL of anhydrous N,N-DMF was added to the mixture. After stirring the solution for 10 min at rt, the formation of the dipotassium salt of **6** was confirmed by a change from clear to curdy solution. 1,8-dibromooctane (53 mg, 0.194 mmol) was added and reaction mixture heated to 60 °C in an oil bath for 48 h. The mixture was evaporated, and the crude was purified by silica gel column chromatography using ethyl acetate/hexane (2:8) to afford yellow solid of **1-C8**, (80 mg, 33% yield).

^{1}H NMR (400 MHz, $CDCl_3$) δ 7.37 (dd, *J* = 9.0, 1.4 Hz, 2H), 7.29 – 7.26 (m, 2H), 7.17 (dd, *J* = 8.9, 6.6 Hz, 2H), 7.09 – 7.04 (m, 4H), 7.04 – 6.98 (m, 4H), 6.96 (dd, *J* = 7.4, 1.8 Hz, 2H), 6.94 – 6.88 (m, 4H), 6.66 (td, *J* = 7.4, 1.0 Hz, 2H), 6.30 (dd, *J* = 8.2, 1.1 Hz, 2H), 3.71 (q, *J* = 4.4 Hz, 2H), 3.64 (td, *J* = 9.1, 3.1 Hz, 2H), 1.39 (d, *J* = 19.5 Hz, 4H), 0.93 – 0.83 (m, 8H).

^{13}C NMR (101 MHz, $CDCl_3$) δ 155.9, 140.7, 138.4, 138, 134.3, 133.2, 132.5, 131.7, 131.6, 130.1, 128.6, 128, 127.9, 127.2, 126.9, 126.4, 124, 119.6, 110, 67.8, 30.2, 29.8, 27.1.

HR-ESI-MS m/z (%): 625.3081 for $[M+H]^+$ calcd. for $C_{46}H_{40}O_2^+$: 625.3101.

S2.2 Synthesis of **2-Cn**

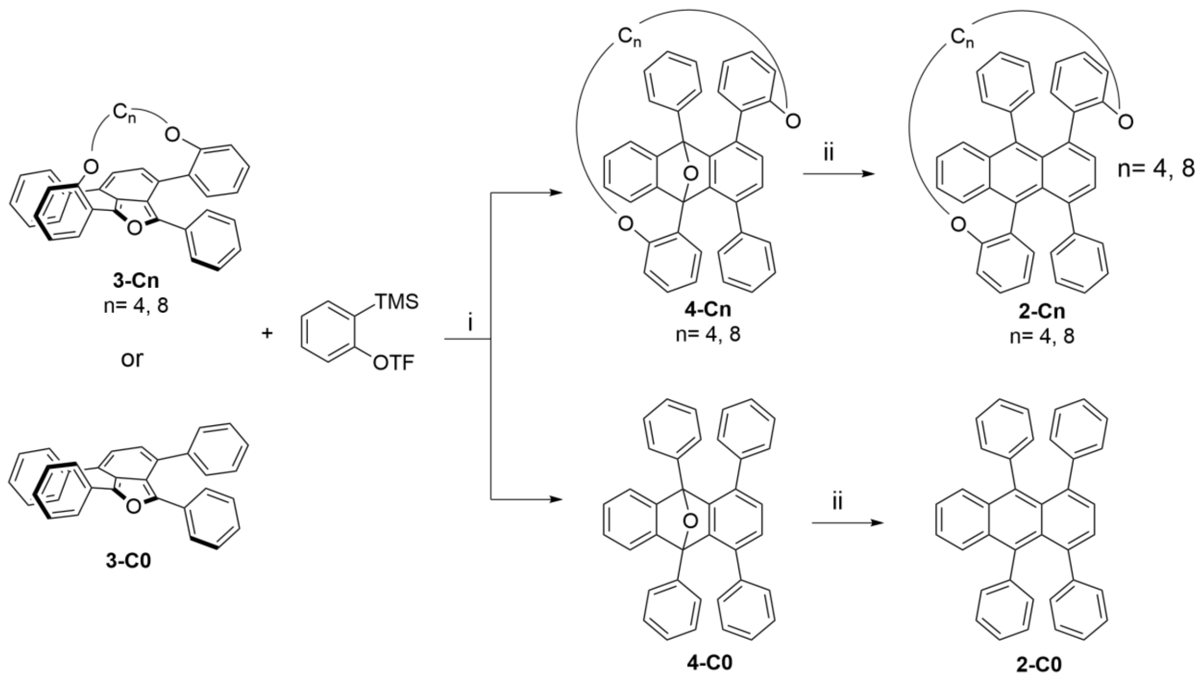

S2.2.1 Synthesis of **4-C0**

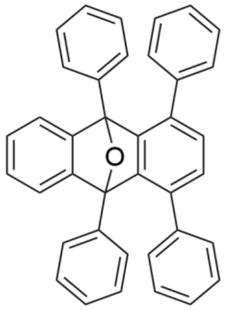

Finely powdered anhydrous CsF (143 mg, 0.946 mmol) was added to a solution of 2-(trimethylsilyl)phenyl trifluoromethanesulfonate (0.070 mL, 0.284 mmol) and **3-C0** (80 mg, 0.189 mmol) in a mixture of acetonitrile (2 mL) and dichloromethane (8 mL), and the mixture was stirred at rt for 12 h. The reaction mixture was filtered, and the solvent was removed under reduced pressure. The residue was purified by silica gel column chromatography with ethyl acetate/hexane (1:9), affording **4-C0** as a white crystalline solid (40 mg, 42 %yield).

^{1}H NMR (500 MHz, $CDCl_3$) δ 7.87 (dd, *J* = 5.3, 3.1 Hz, 1H), 7.56 – 7.52 (m, 2H), 7.34 (dd, *J* = 5.3, 3.0 Hz, 1H), 7.21 (s, 1H), 7.17 (dt, *J* = 7.1, 1.5 Hz, 2H), 7.13 (ddd, *J* = 7.7, 6.8, 1.3 Hz, 2H), 7.10 – 7.02 (m, 4H).

^{13}C NMR (126 MHz, $CDCl_3$) δ 151.2, 149.2, 139.8, 135.6, 133.8, 129.8, 129.2, 128.6, 128.4, 127.9, 127.9, 127.1, 126.2, 123.4, 92.3.

HR-ESI-MS m/z (%): 500.2134 for (100, [M]) calcd. for $C_{38}H_{26}O$: 500.2100.

S2.2.2 Synthesis of **2-C0**

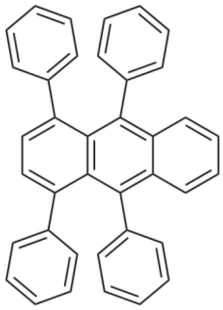

To a solution of **4-C0** (40 mg 0.079 mmol) and anhydrous sodium iodide (59 mg, 0.399 mmol) in 10 mL of dry MeCN was added trimethylsilyl chloride (0.05 mL, 0.399 mmol) at 0 °C under argon and stirred for 30 min. The mixture was quenched with the addition of 1 mL of 5% aqueous $Na_2S_2O_3$ and extracted with diethyl ether (50 mL). The organic layer was washed with 5% aqueous $Na_2S_2O_3$ (2 mL) and brine (5 mL), then dried over anhydrous $MgSO_4$. The solvent was evaporated under reduced pressure. The residue thus obtained was purified by silica gel column chromatography using hexane, to yield **2-C0** as a pale-yellow solid (18 mg, 68% yield).

^{1}H NMR (400 MHz, $CDCl_3$) δ 7.63 – 7.58 (m, 2H), 7.29 – 7.24 (m, 2H), 7.14 (s, 2H), 7.06 (d, *J* = 0.8 Hz, 10H), 6.99 – 6.95 (m, 6H), 6.93 – 6.89 (m, 4H).

^{13}C NMR (101 MHz, $CDCl_3$) δ 144.9, 140.9, 140.9, 137.6, 133.3, 131, 130.8, 130.2, 128.7, 127.5, 127.4, 126.9, 126.6, 125.7, 125.4.

HR-ESI-MS m/z (%) 482.2029 for (100, [M]) calcd. for $C_{38}H_{26}$: 482.2018.

S2.2.3 Synthesis of **4-C4**

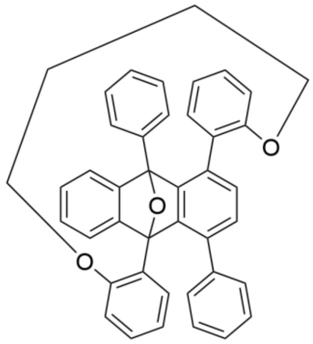

Finely powdered anhydrous CsF (89 mg, 0.589 mmol) was added to a solution of 2-(trimethylsilyl)phenyl trifluoromethanesulfonate (0.043 mL, 0.176 mmol) and **3-C4** (60 mg, 0.117 mmol) in acetonitrile (5 mL) and dichloromethane (5mL), and the mixture was stirred at rt for 12 h. The reaction mixture was filtered, and the solvent was removed under reduced pressure. The residue was purified by silica gel column chromatography using ethyl acetate/hexane (1:9), affording **4-C4** as a white crystalline solid (30 mg, 43% yield).

^{1}H NMR (400 MHz, $CDCl_3$) δ 8.03 (dd, *J* = 7.7, 1.7 Hz, 1H), 7.94 (d, *J* = 7.3 Hz, 1H), 7.40 (dd, *J* = 7.2, 0.8 Hz, 1H), 7.37 (dd, *J* = 3.3, 1.4 Hz, 1H), 7.36 – 7.32 (m, 2H), 7.25 – 7.21 (m, 2H), 7.08 – 7.06 (m, 5H), 7.05 – 7.00 (m, 4H), 6.99 – 6.96 (m, 3H), 6.94 (dd, *J* = 7.4, 1.1 Hz, 1H), 6.90 (d, *J* = 7.8 Hz, 1H), 6.80 – 6.74 (m, 2H), 6.39 (dd, *J* = 8.2, 1.1 Hz, 1H), 6.00 (dd, *J* = 8.1, 1.1 Hz, 1H), 3.75 (dt, *J* = 8.3, 2.8 Hz, 1H), 3.53 (dd, *J* = 11.5, 8.4 Hz, 1H), 3.31 – 3.20 (m, 2H), 2.28 – 2.18 (m, 1H), 1.49 – 1.36 (m, 3H).

^{13}C NMR (101 MHz, $CDCl_3$) δ 158.6, 156.1, 154.5, 149.7, 149, 147.6, 139.7, 135.4, 134.8, 131, 130.9, 130.3, 129.2, 128.8, 128.6, 128.5, 127.9, 127.4, 126.8, 126.7, 126.6, 126.4, 126.4, 126.1, 125.3, 123.9, 122.8, 121.1, 119.8, 119, 111.1, 110.1, 90.3, 89.8, 66.8, 65.4, 26.8, 25.2.

HR-ESI-MS m/z (%): 585.2442 for $[M+H]^+$ calcd. for $C_{42}H_{33}O_3^+$: 585.2424.

S2.2.4 Synthesis of **2-C4**

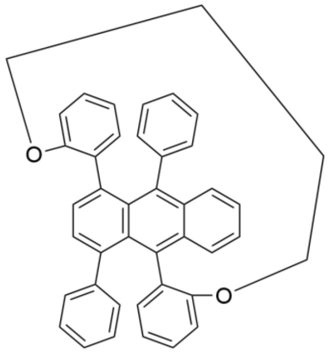

To a solution of **4-C4** (30 mg. 0.051 mmol) and anhydrous sodium iodide (38 mg, 0.255 mmol) in 10 mL of dry MeCN:DCM (1:1) was added trimethylsilyl chloride (0.032 mL, 0.255 mmol) at 0 ºC under argon and stirred for 30 min. The mixture was quenched with the addition of 1 mL of 5% aqueous $Na_2S_2O_3$ and extracted with diethyl ether (30 mL). The organic layer was washed with 5% aqueous $Na_2S_2O_3$ (2 mL) and brine (5 mL) and dried over anhydrous $MgSO_4$ and the solvent was evaporated under reduced pressure. The residue thus obtained was purified by silica gel column chromatography using hexane as eluent to give **2-C4** as a pale-yellow solid (10 mg, 34% yield).

^{1}H NMR (500 MHz, CDCl3) δ 7.75 – 7.61 (m, 1H), 7.53 – 7.43 (m, 1H), 7.25 – 7.18 (m, 3H), 7.16 – 7.13 (m, 1H), 7.10 (tt, J = 7.3, 1.4 Hz, 1H), 7.07 – 7.04 (m, 2H), 7.03 (d, J = 1.7 Hz, 3H), 7.01 (m, 1H), 7.00 – 6.97 (m, 1H), 6.97 – 6.91 (m, 4H), 6.89 (dd, J = 7.4, 1.7 Hz, 2H), 6.61 – 6.52 (m, 3H), 6.36 (dd, J = 8.1, 1.1 Hz, 1H), 3.67 (dt, J = 8.4, 3.3 Hz, 1H), 3.61 (dt, J = 8.2, 3.1 Hz, 1H), 3.30 – 3.21 (m, 1H), 3.23 – 3.09 (m, 1H), 1.23 – 1.06 (m, 2H), 1.04 – 0.93 (m, 1H), 0.81 – 0.60 (m, 1H).

^{13}C NMR (126 MHz, $CDCl_3$) δ 156.7, 155.7, 144.7, 140.7, 140.3, 137.5, 136.7, 134.3, 133.8, 132.54, 132.1, 132, 132, 131.8, 130.5 (d, *J* = 9.7 Hz), 130.2, 129.6, 129.2, 128.2 (d, *J* = 10.1 Hz), 127.8, 127.7, 127.3 (d, *J* = 2.1 Hz), 127, 126.9, 126.8, 126.7, 126.5, 126.4, 125.8, 125.6, 124.5, 124.4, 120, 119.3, 111, 109.6, 66.5, 66.2, 25.3.
HR-ESI-MS m/z (%): 568.2392 for (100, $[M]^+$) calcd. for $C_{42}H_{32}O_2^+$: 568.2397.

S2.2.5 Synthesis of **4-C8**

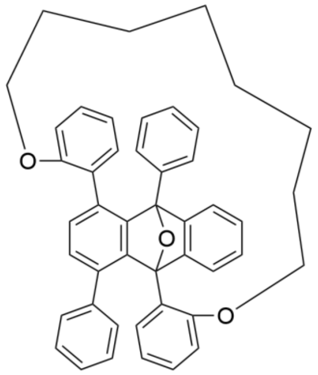

Finely powdered anhydrous CsF (94 mg, 0.619 mmol) was added to a solution of 2-(trimethylsilyl)phenyl trifluoromethanesulfonate (0.045 mL, 0.186 mmol) and **3-C8** (70 mg, 0.123 mmol) in a mixture acetonitrile (5 mL) and dichloromethane (5 mL), and the mixture was stirred at rt for 12 h. The reaction mixture was filtered, and the solvent was removed under reduced pressure. The residue was purified using silica gel column chromatography with ethyl acetate/hexane, affording **4-C8** as a white crystalline solid (40 mg, 50% yield).

^{1}H NMR (400 MHz, $CDCl_3$) δ 8.09 (dd, *J* = 7.7, 1.7 Hz, 1H), 7.96 – 7.92 (m, 1H), 7.80 – 7.76 (m, 1H), 7.71 – 7.66 (m, 2H), 7.35 – 7.27 (m, 2H), 7.22 – 7.19 (m, 2H), 7.18 (d, *J* = 8.0 Hz, 1H), 7.10 – 6.99 (m, 9H), 6.84 (td, *J* = 7.5, 1.1 Hz, 1H), 6.73 (dd, *J* = 8.3, 1.1 Hz, 1H), 6.69 (dd, *J* = 7.4, 1.8 Hz, 1H), 6.63 – 6.58 (m, 1H), 6.26 (dd, *J* = 8.3, 1.1 Hz, 1H), 4.18 (ddd, *J* = 8.8, 4.9, 2.5 Hz, 1H), 3.93 (ddd, *J* = 10.3, 8.7, 1.8 Hz, 1H), 3.63 – 3.53 (m, 1H), 3.40 (dd, *J* = 8.2, 4.2 Hz, 1H), 1.96 (d, *J* = 9.5 Hz, 1H), 1.86 – 1.76 (m, 1H), 1.66 (dt, *J* = 8.1, 6.0 Hz, 1H), 1.61 – 1.47 (m, 3H), 1.46 – 1.32 (m, 3H), 1.32 – 1.20 (m, 2H), 1.06 (dd, *J* = 9.0, 4.0 Hz, 1H).

^{13}C NMR (101 MHz, CDCl3) δ 158.5, 156, 152.5, 151.2, 149.5, 148.5, 139.2, 135, 134.5, 132.1, 131.7, 130.4, 130.1, 129.8, 129, 128.7, 128.7, 128.3, 127.7, 127.1, 126.6, 126, 125.9, 125.8, 124.3, 123.2, 121.7, 119.4, 119.2, 110.9, 110.8, 91.8, 90.7, 69.7, 68.1, 31.9, 30.1, 29.6, 28.8, 28.5, 28, 27.2, 23, 14.4.

HR-ESI-MS m/z (%): 641.3060 for $[M+H]^+$ calcd. for $C_{46}H_{41}O_3^+$: 641.3050.

S2.2.6 Synthesis of **2-C8**

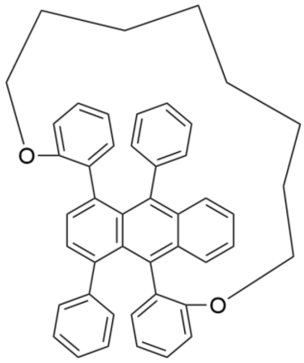

To a solution of **4-C8** (40 mg. 0.062mmol) and anhydrous sodium iodide (47 mg, 0.311 mmol) in 10 mL of dry MeCN:DCM (1:1) was added trimethylsilyl chloride (0.039 mL, 0.311 mmol) at 0 ºC under argon and stirred for 30 min. The mixture was quenched with the addition of 1 mL of 5% aqueous $Na_2S_2O_3$ and extracted with diethyl ether (30 mL). The organic layer was washed with 5% aqueous $Na_2S_2O_3$ (2 mL) and brine (5 mL) then dried over anhydrous $MgSO_4$. The solvent was evaporated under reduced pressure. The residue thus obtained was purified by silica gel column chromatography using hexane as the eluent to give **2-C8** as a pale-yellow solid (16 mg, 41% yield).

^{1}H NMR (500 MHz, $CDCl_3$) δ 7.35 (d, *J* = 14.8 Hz, 2H), 7.24 – 7.21 (m, 1H), 7.18 – 7.11 (m, 4H), 7.06 – 7.01 (m, 4H), 7.00 – 6.93 (m, 6H), 6.93 – 6.87 (m, 3H), 6.73 (td, *J* = 7.4, 1.0 Hz, 1H), 6.65 (td, *J* = 7.4, 1.1 Hz, 1H), 6.39 (dd, *J* = 8.4, 1.1 Hz, 1H), 6.34 (dd, *J* = 8.3, 1.1 Hz, 1H), 3.81 (ddd, *J* = 9.1, 5.8, 3.6 Hz, 1H), 3.77 – 3.71 (m, 2H), 3.66 (td, *J* = 8.6, 3.3 Hz, 1H), 1.57 – 1.53 (m, 1H), 1.43 – 1.24 (m, 6H), 1.07 (h, *J* = 7.0 Hz, 2H), 1.00 – 0.92 (m, 2H), 0.77 (dt, *J* = 12.3, 6.4 Hz, 1H).

^{13}C NMR (126 MHz, CDCl3) δ 156.2, 155.4, 144.8, 140.4, 134.2, 133.3, 132.6, 130. 9, 130.8, 129.7, 129.4, 129.1, 128.5, 127.3, 127.1, 127, 126.8, 126.8, 126.3, 126.2, 126.1, 125.7, 125, 124.6, 124.3, 119.5, 119.4, 109.8, 109.5, 67.6, 66.8, 31.5, 29.3, 29.2, 28.8, 28.6, 26.7, 25.9, 22.6, 14.1.

HR-ESI-MS m/z (%): 624.3022 for (100, $[M]^+$) calcd. for $C_{46}H_{40}O_2^+$: 624.3028.

S3 Characterization

S3.1 NMR

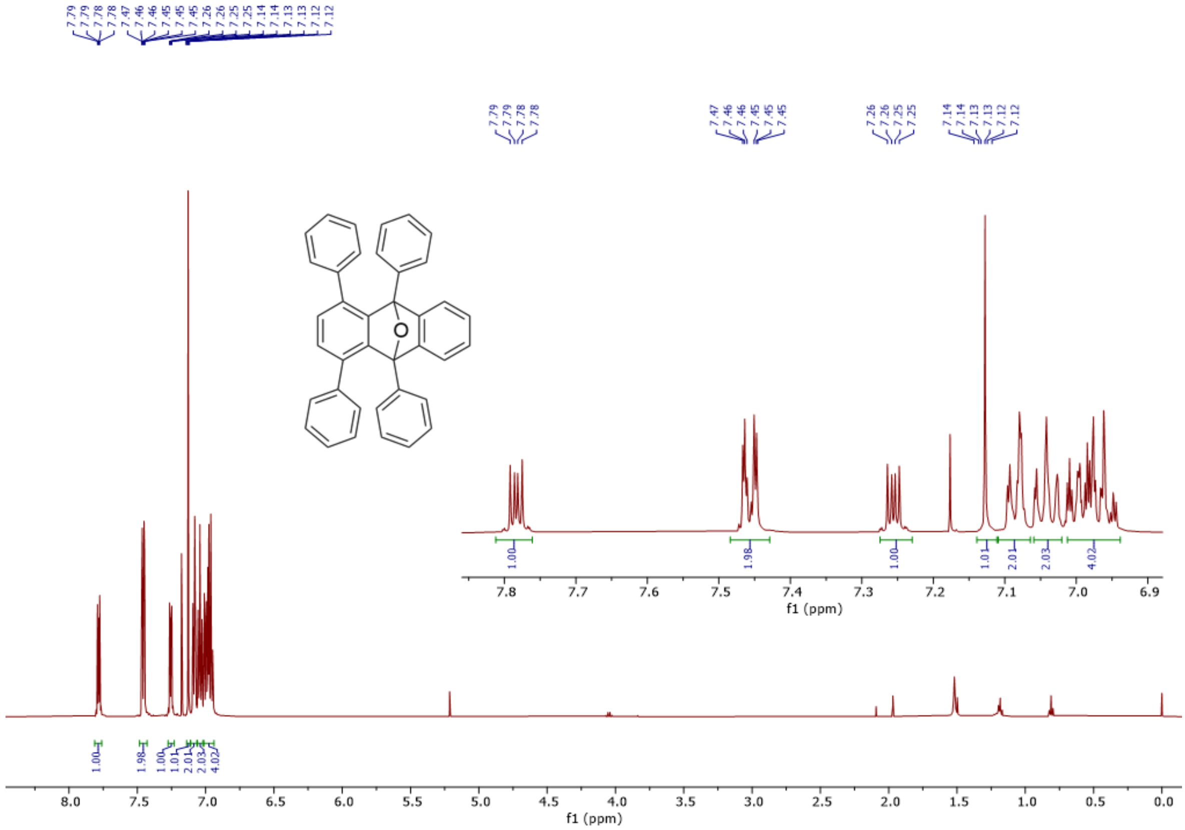

Figure S1. ^{1}H NMR (500 MHz) of **4-C0** in $CDCl_3$, measured at 298 K.

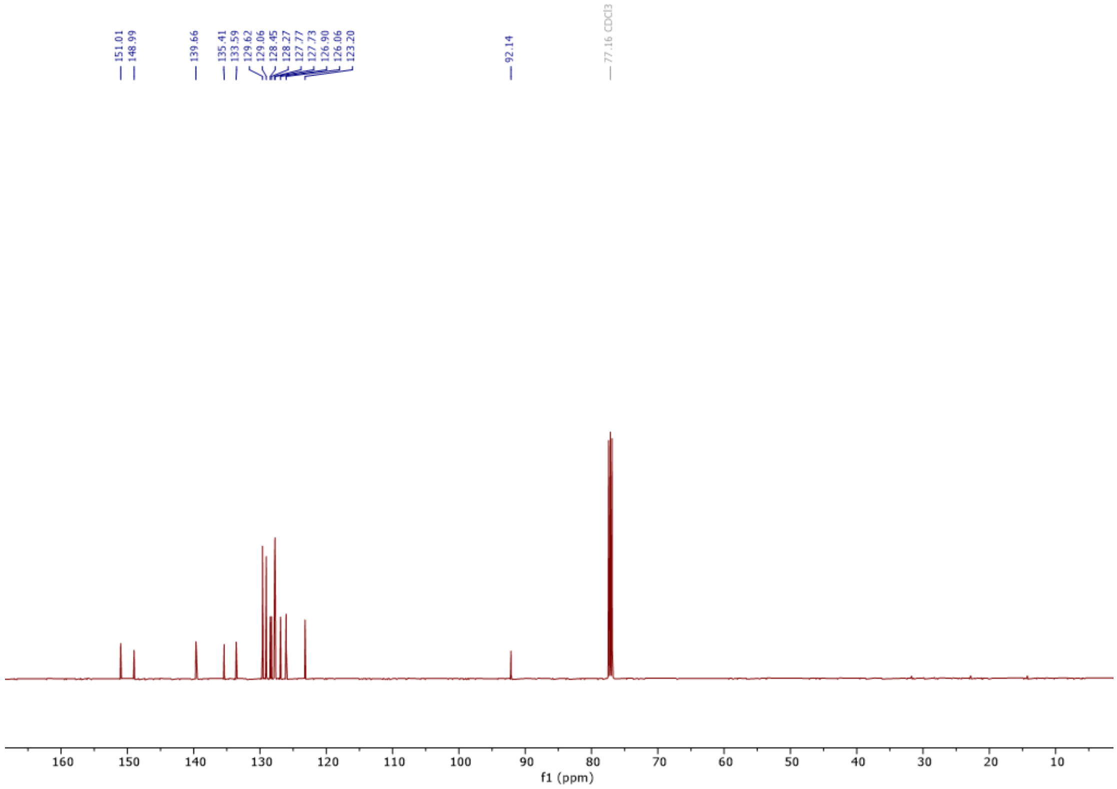

Figure S2. ^{13}C NMR (126 MHz) of **4-C0** in $CDCl_3$, measured at 298 K.

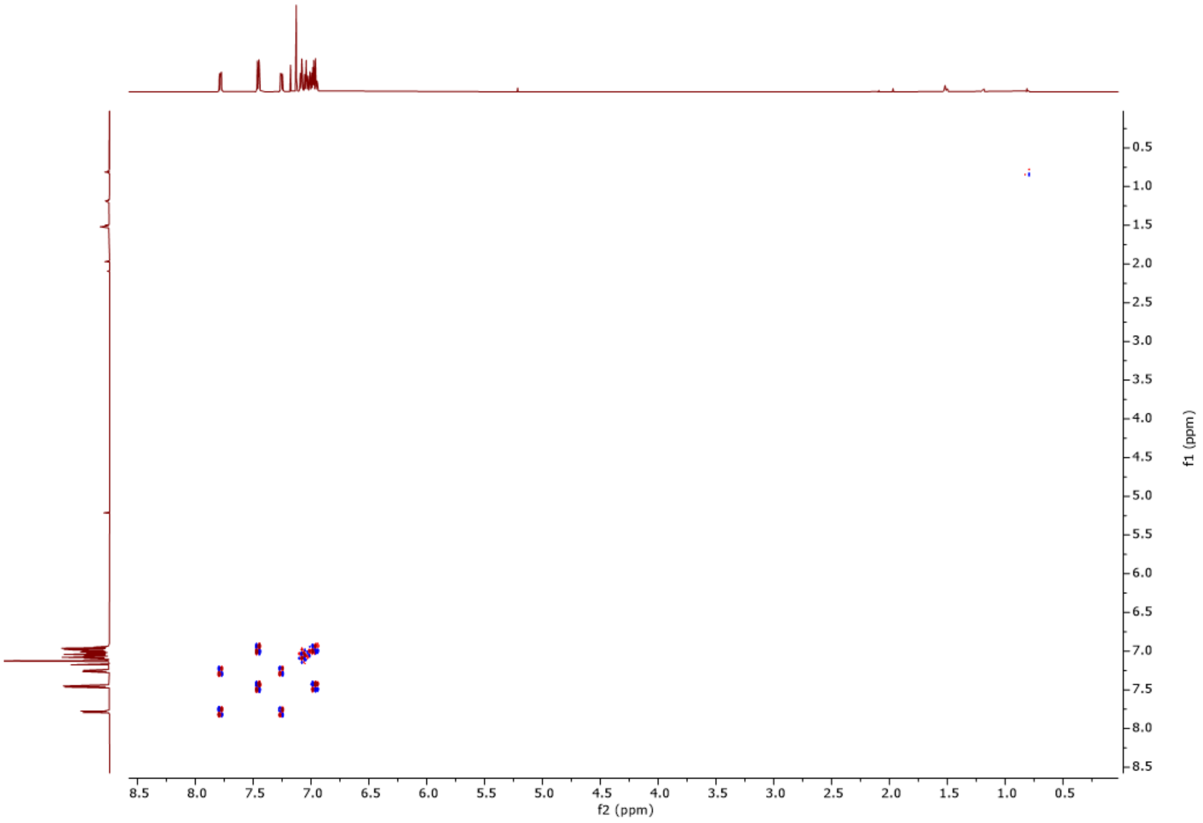

Figure S3. COSY NMR (400 MHz) of **4-C0** in $CDCl_3$, measured at 298 K.

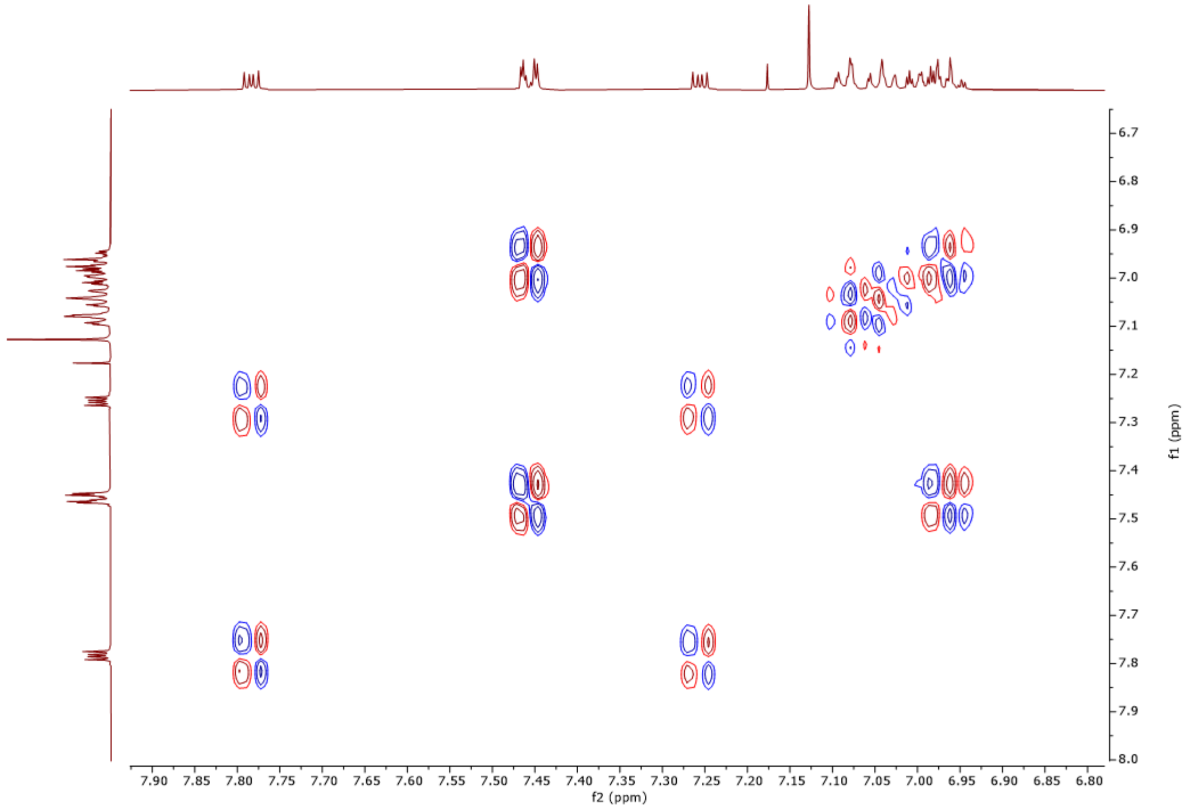

Figure S4. COSY NMR (400 MHz) of **4-C0** in $CDCl_3$, measured at 298 K (expansion in aromatic region).

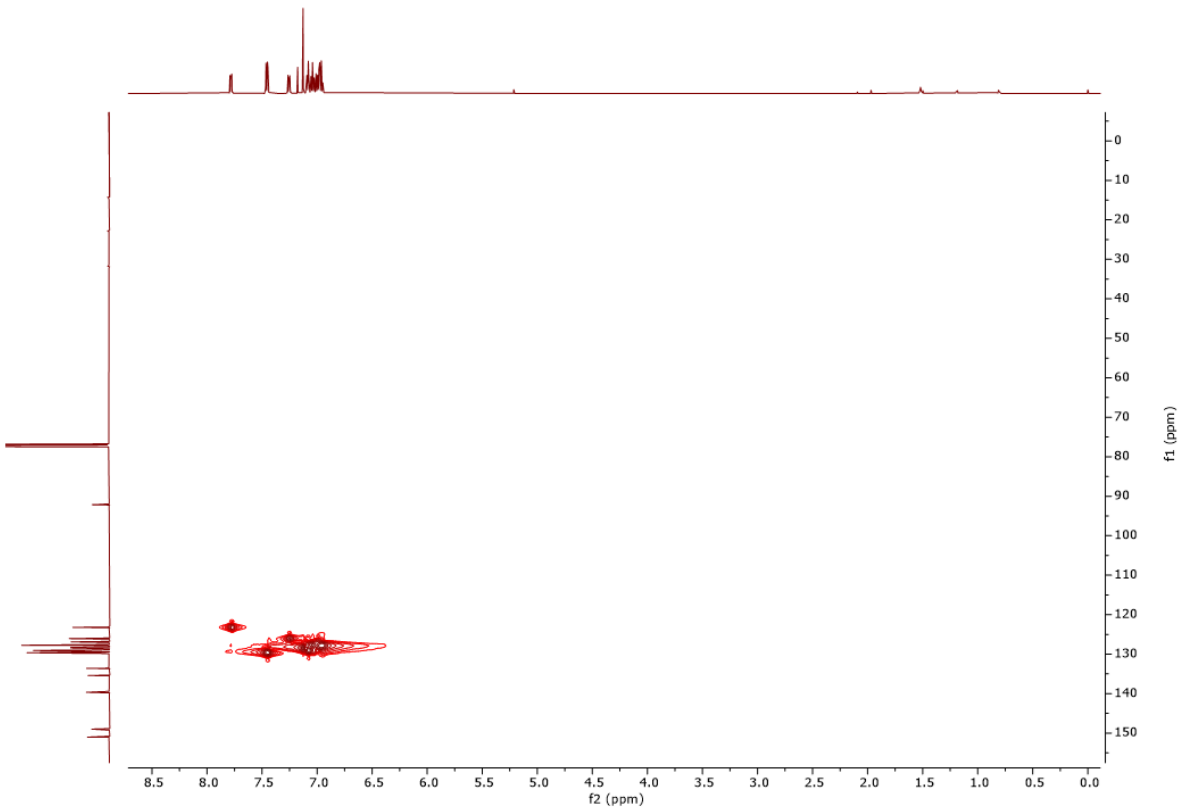

Figure S5. HSQC NMR (400 MHz) of **4-C0** in $CDCl_3$, measured at 298 K.

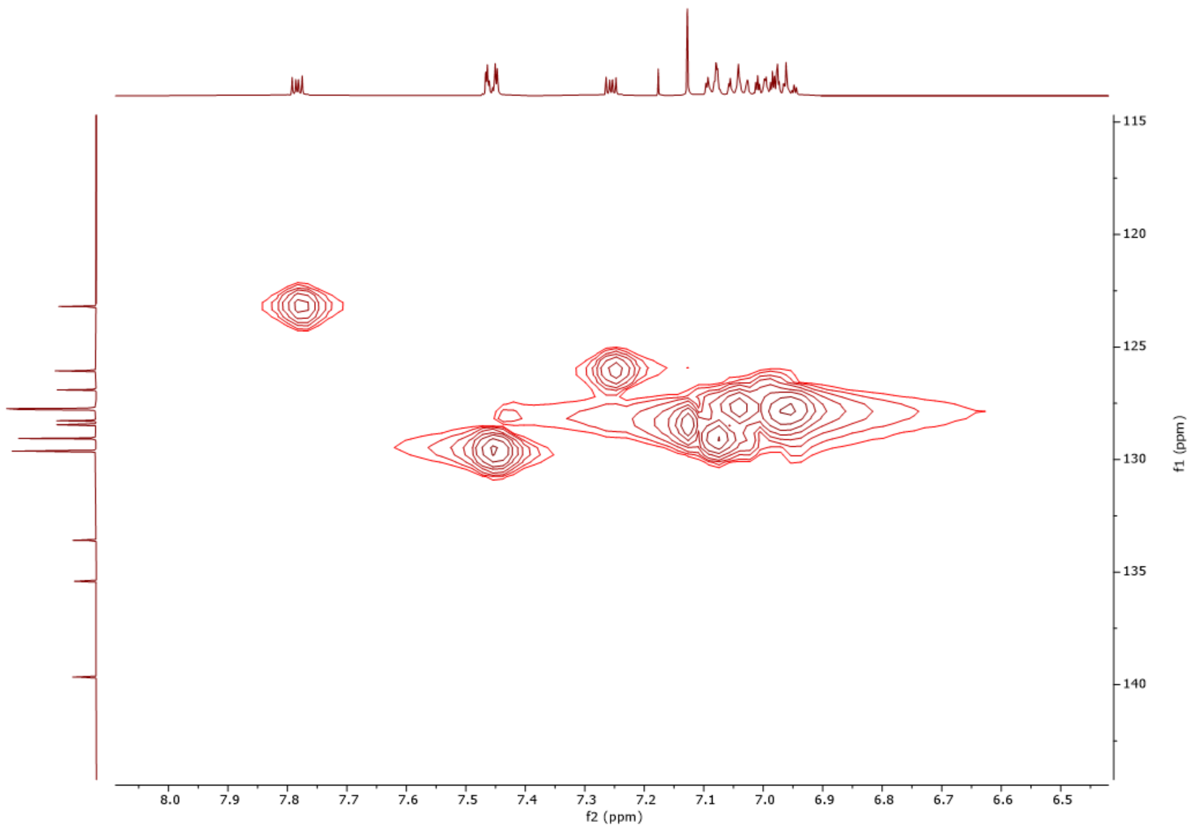

Figure S6. HSQC NMR (400 MHz) of **4-C0** in $CDCl_3$, measured at 298 K (expansion in aromatic region).

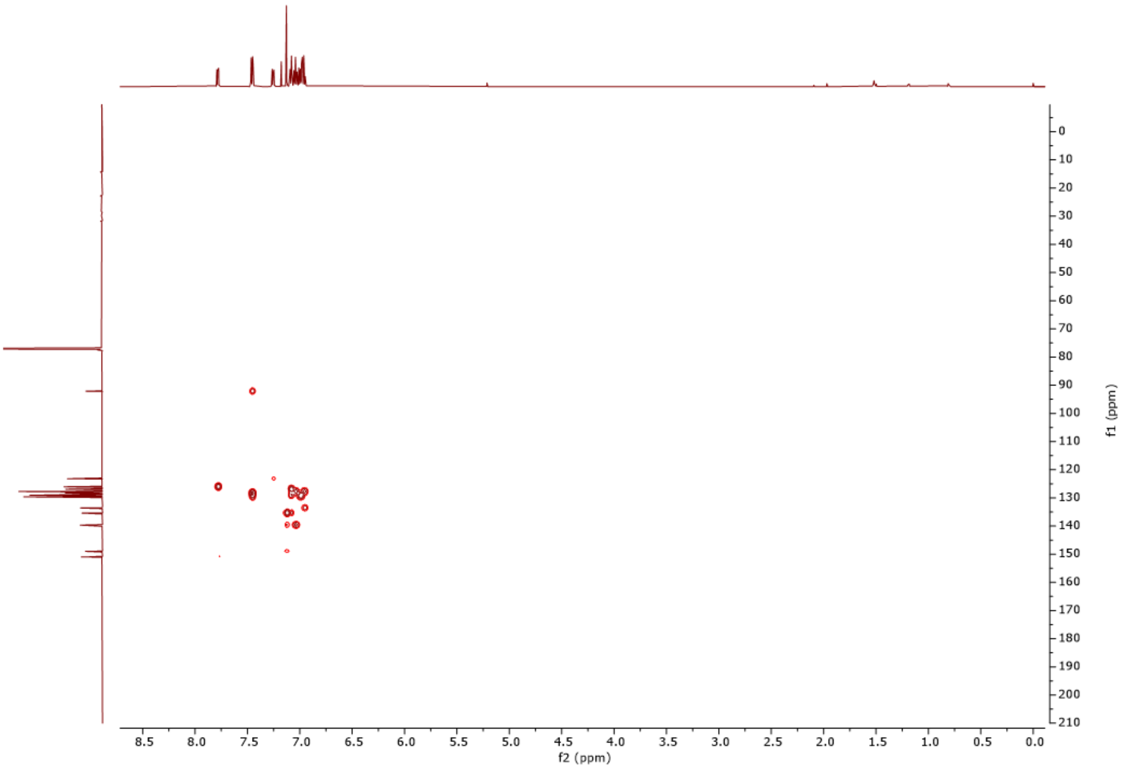

Figure S7. HMBC NMR (400 MHz) of **4-C0** in $CDCl_3$, measured at 298 K.

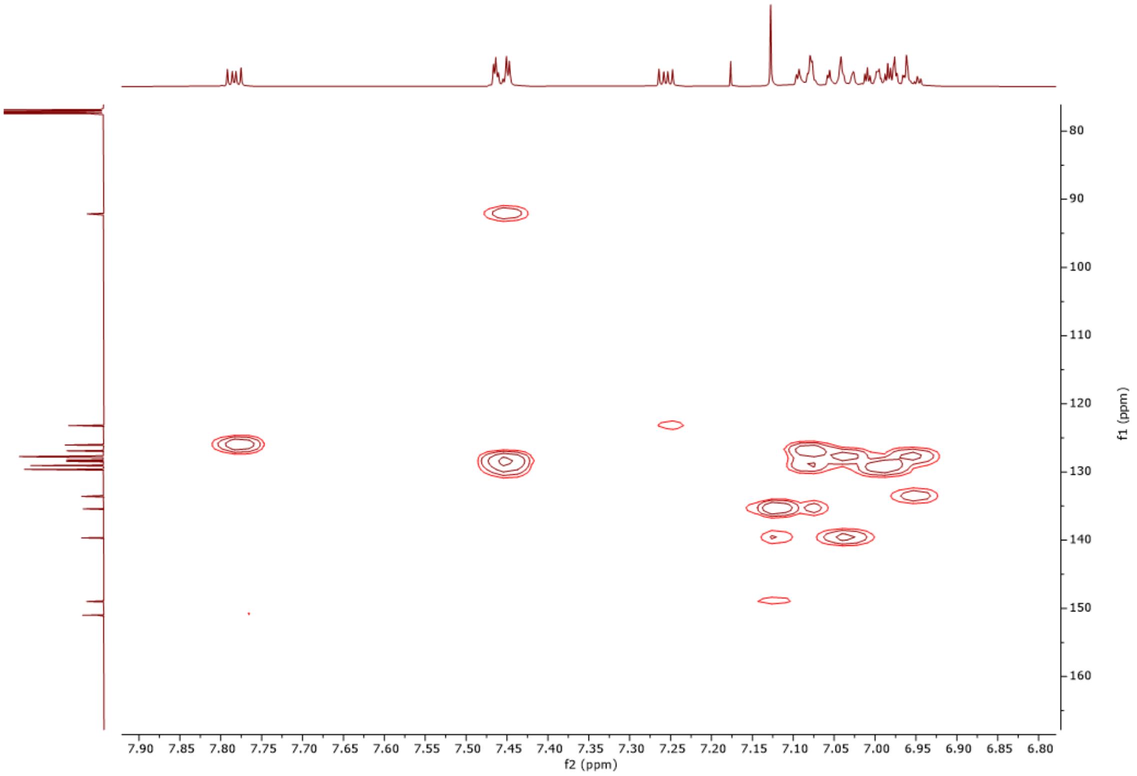

Figure S8. HMBC NMR (400 MHz) of **4-C0** in $CDCl_3$, measured at 298 K (expansion in aromatic region).

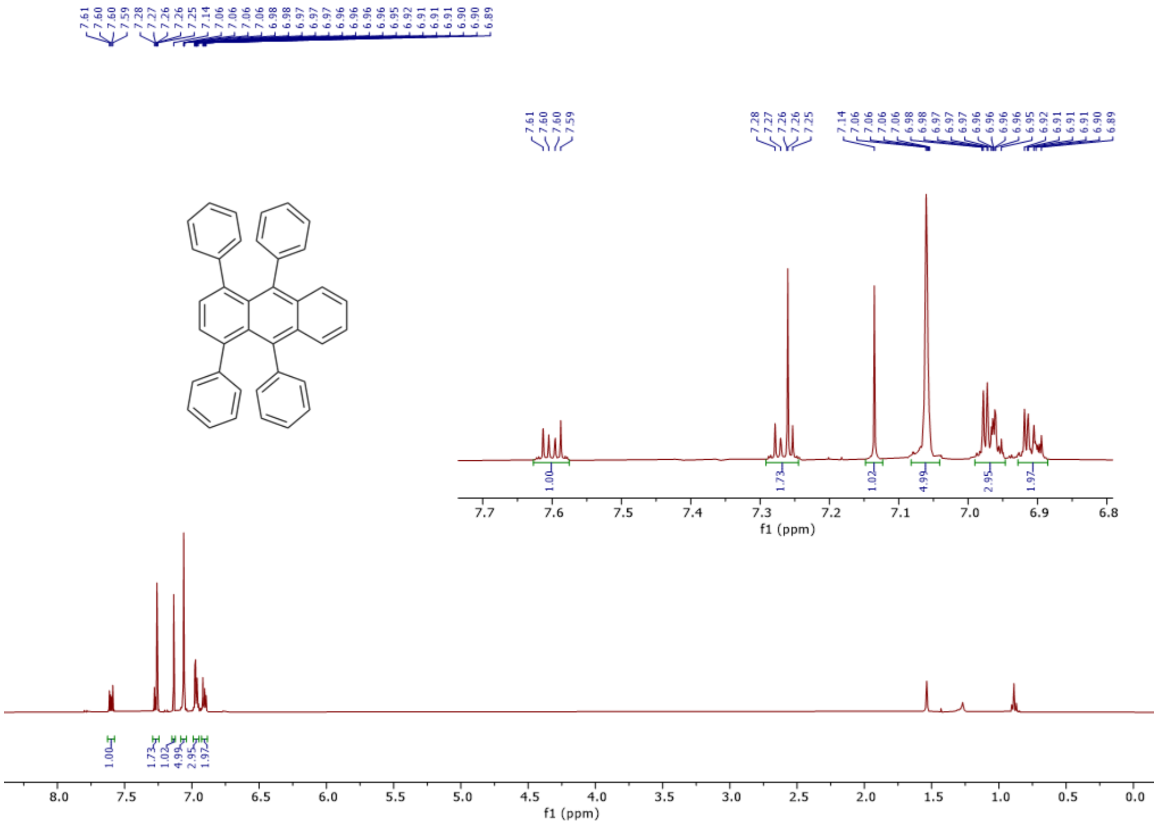

Figure S9. ^{1}H NMR (500 MHz) of **2-C0** in $CDCl_3$, measured at 298 K.

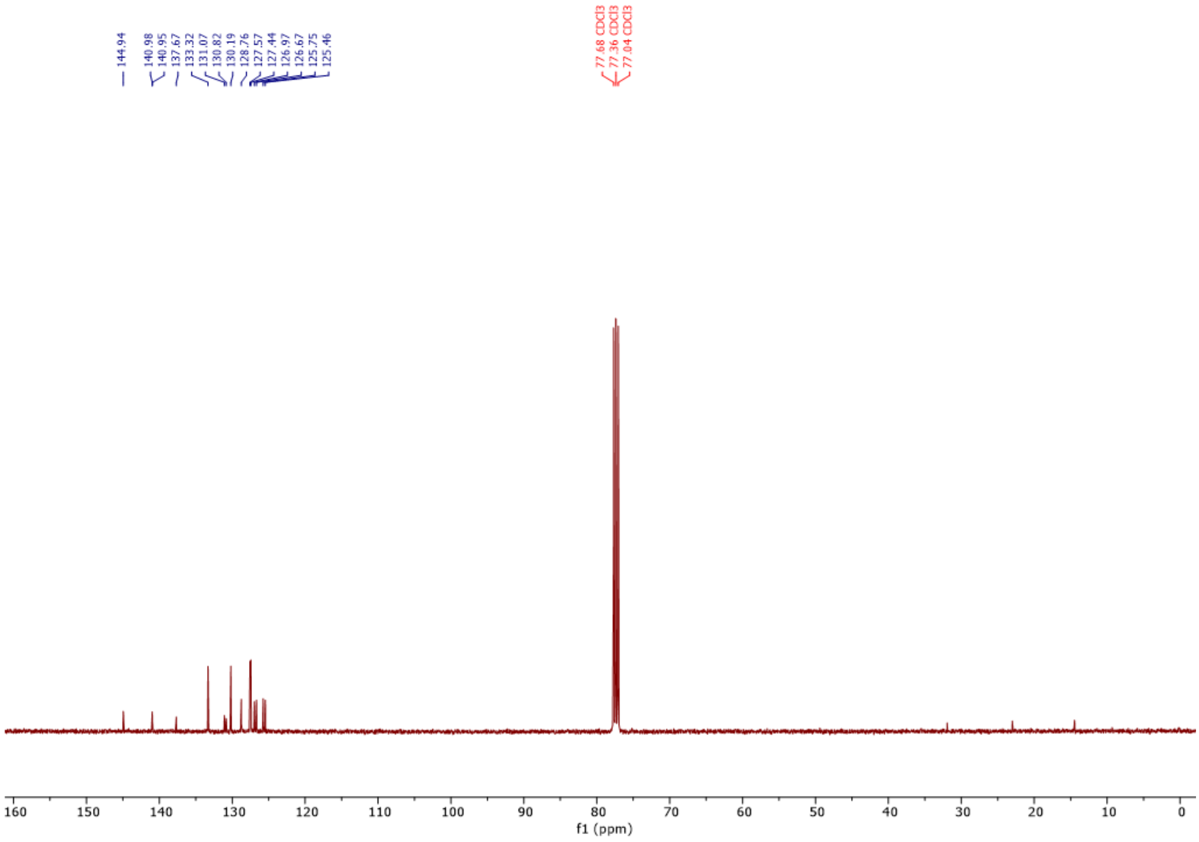

Figure S10. ^{13}C NMR (126 MHz) of **2-C0** in $CDCl_3$, measured at 298 K.

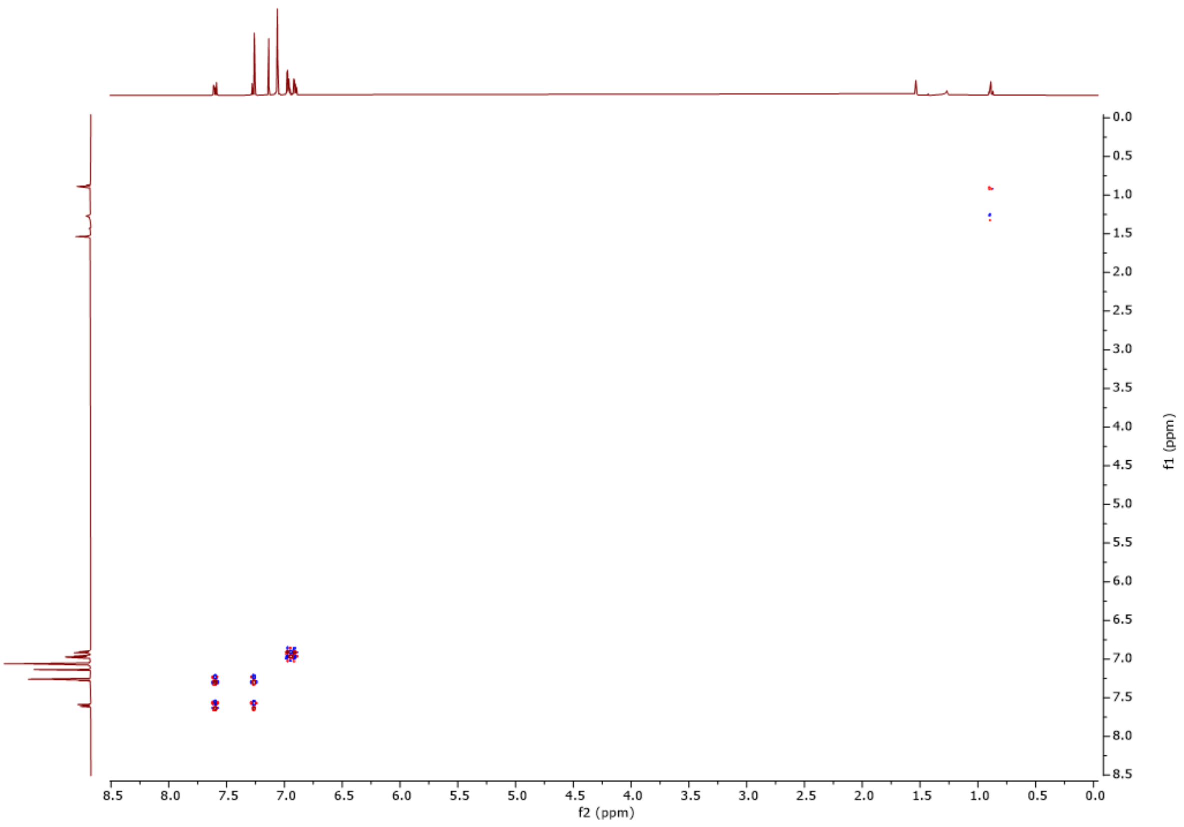

Figure S11. COSY NMR (400 MHz) of **2-C0** in $CDCl_3$, measured at 298 K.

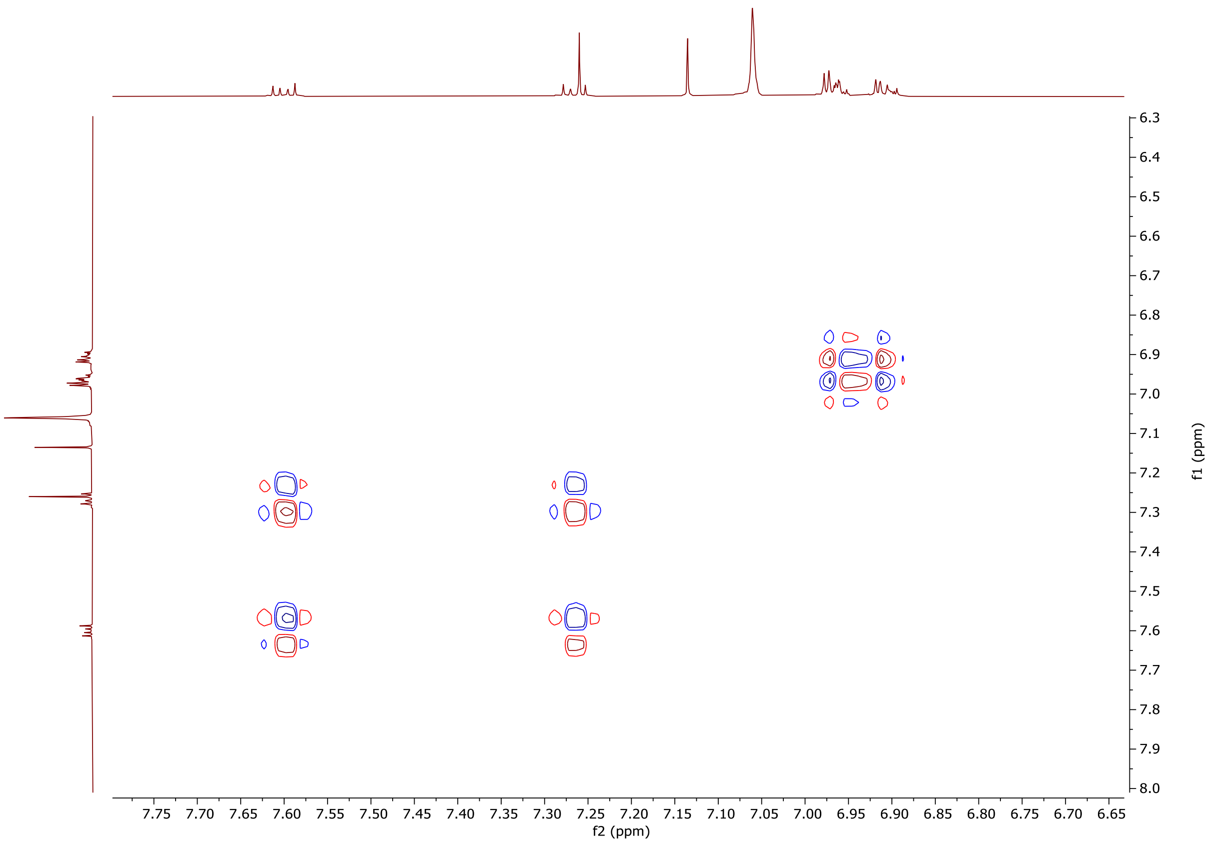

Figure S12. COSY NMR (400 MHz) of **2-C0** in $CDCl_3$, measured at 298 K (expansion in aromatic region).

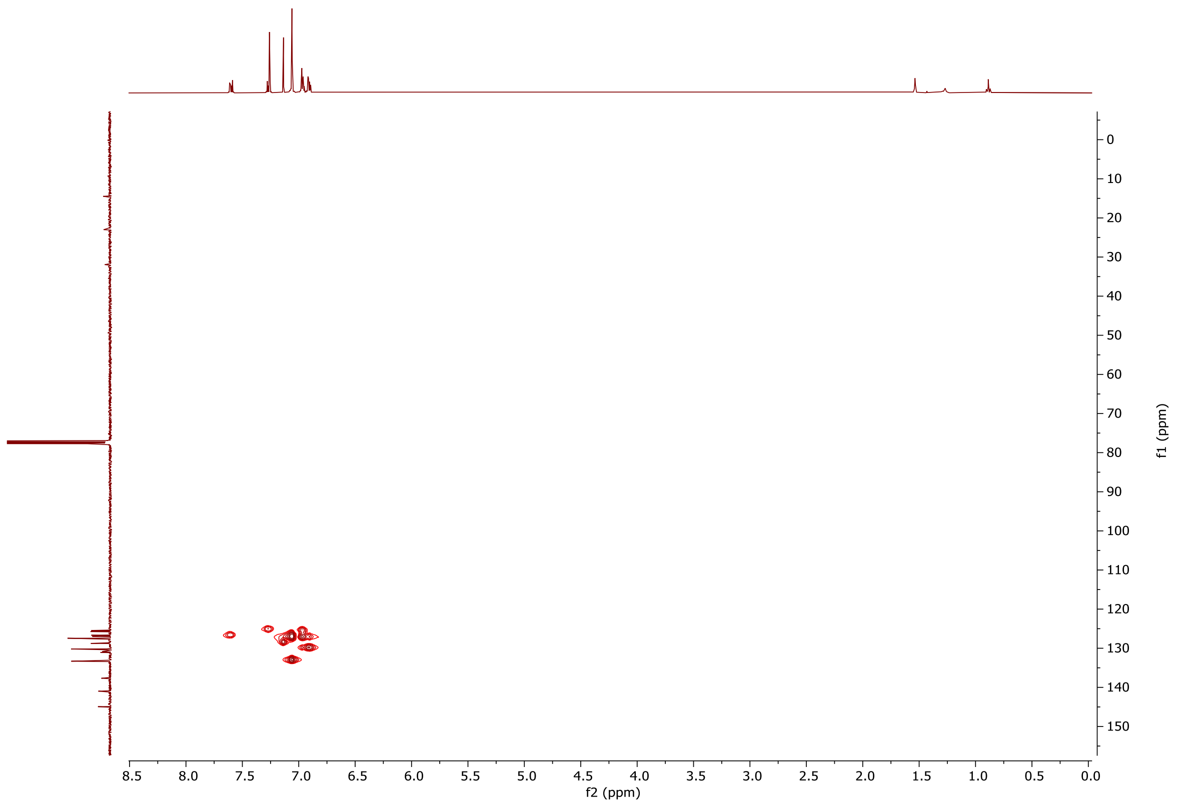

Figure S13. HSQC NMR (400 MHz) of **2-C0** in $CDCl_3$, measured at 298 K.

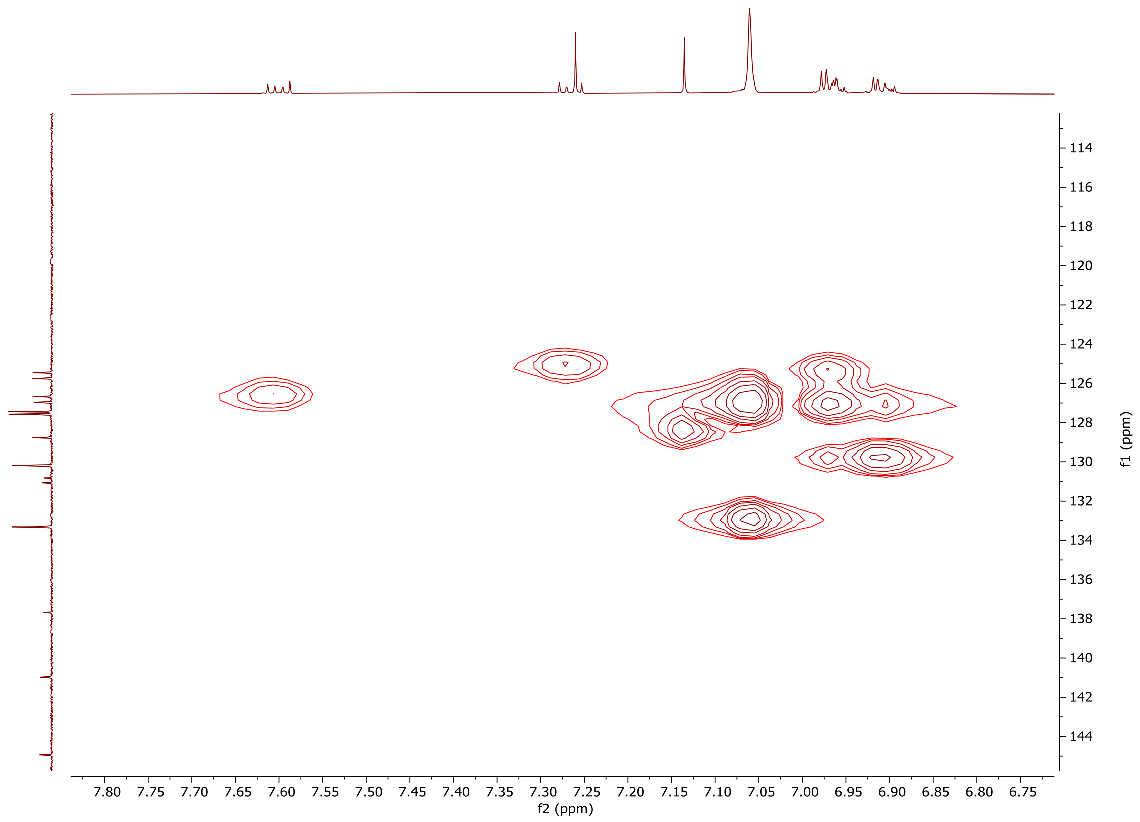

Figure S14. HSQC NMR (400 MHz) of **2-C0** in $CDCl_3$, measured at 298 K (expansion in aromatic region).

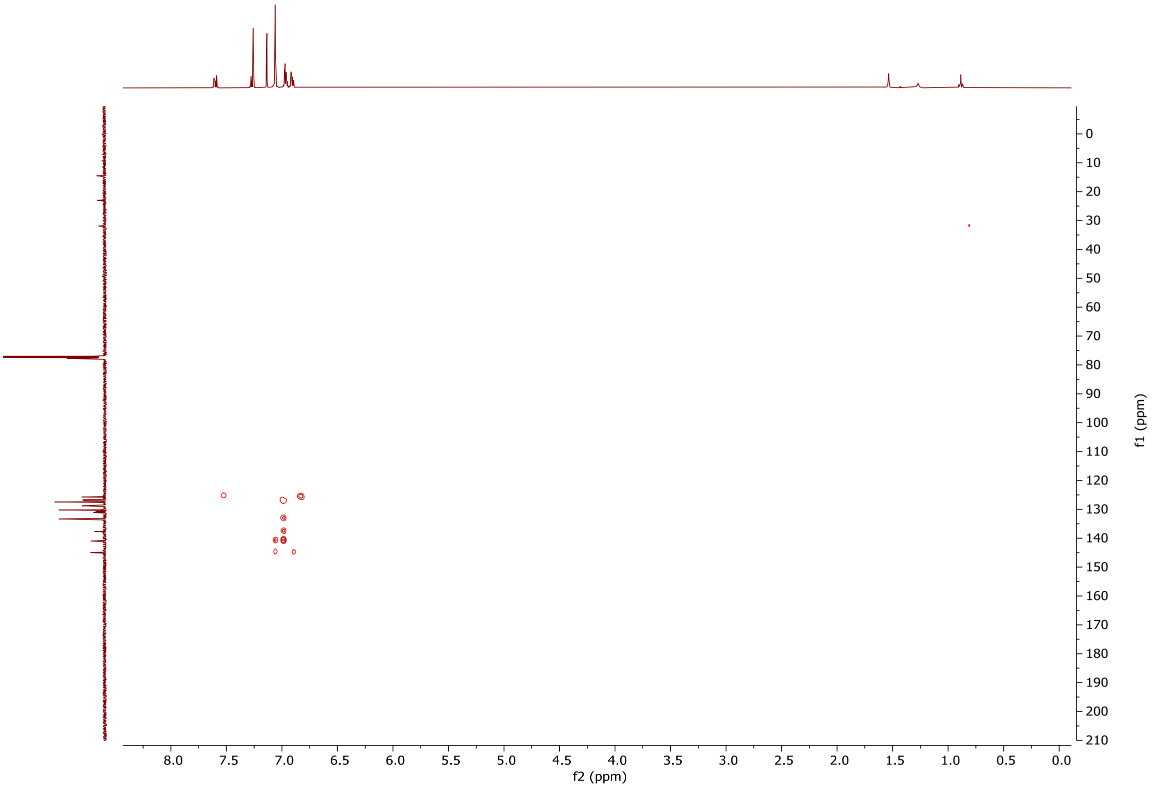

Figure S15. HMBC NMR (400 MHz) of **2-C0** in $CDCl_3$, measured at 298 K.

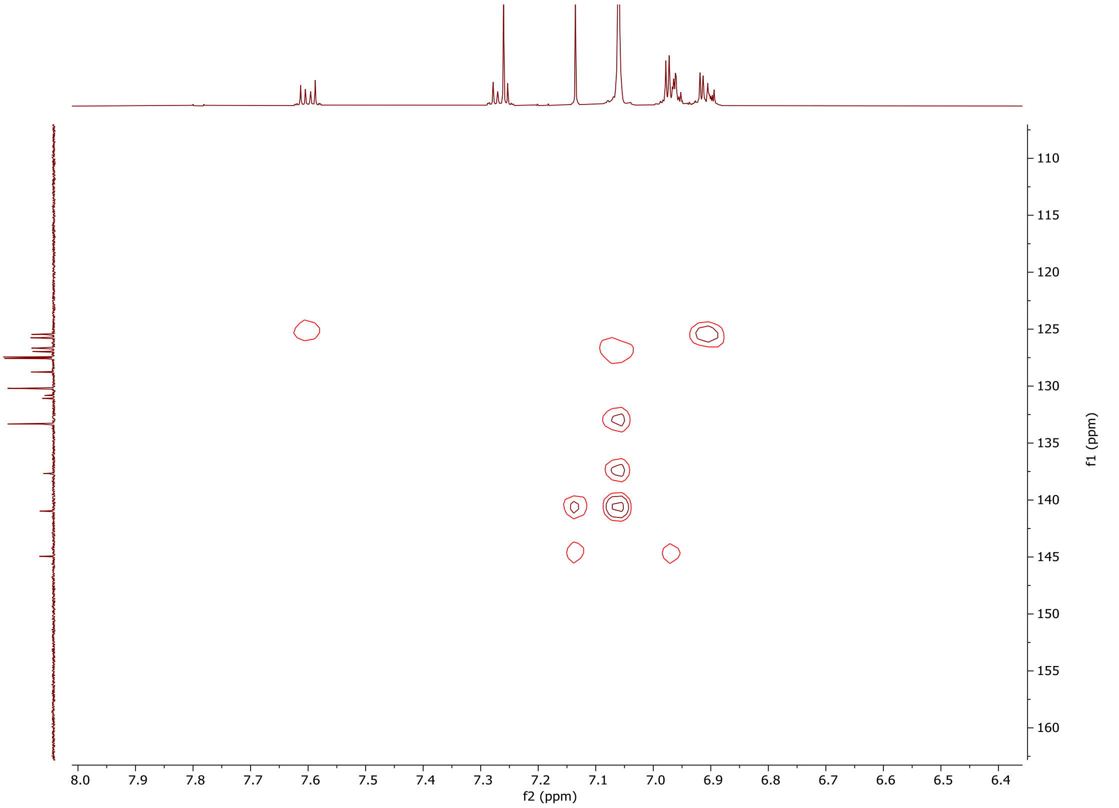

Figure S16. HMBC NMR (400 MHz) of **2-C0** in $CDCl_3$, measured at 298 K (expansion in aromatic region).

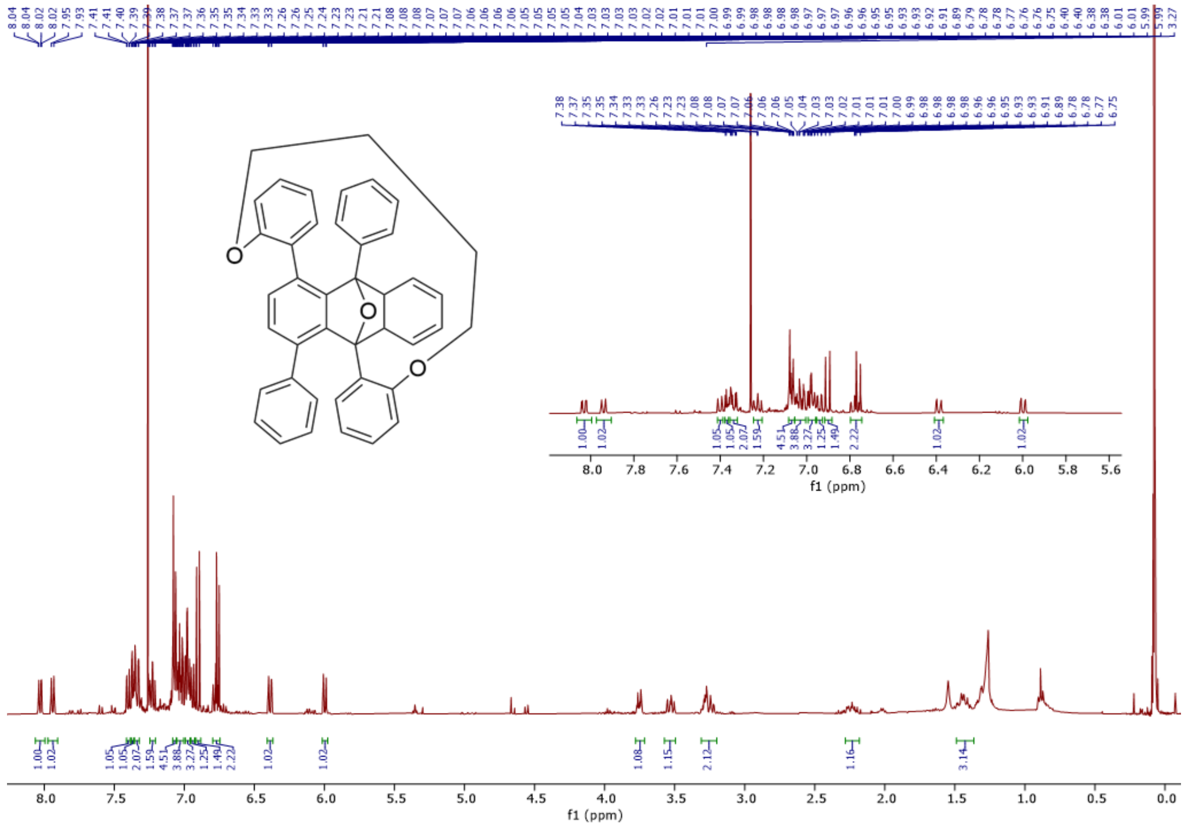

Figure S17. ^{1}H NMR (400 MHz) of **4-C4** in $CDCl_3$, measured at 298 K.

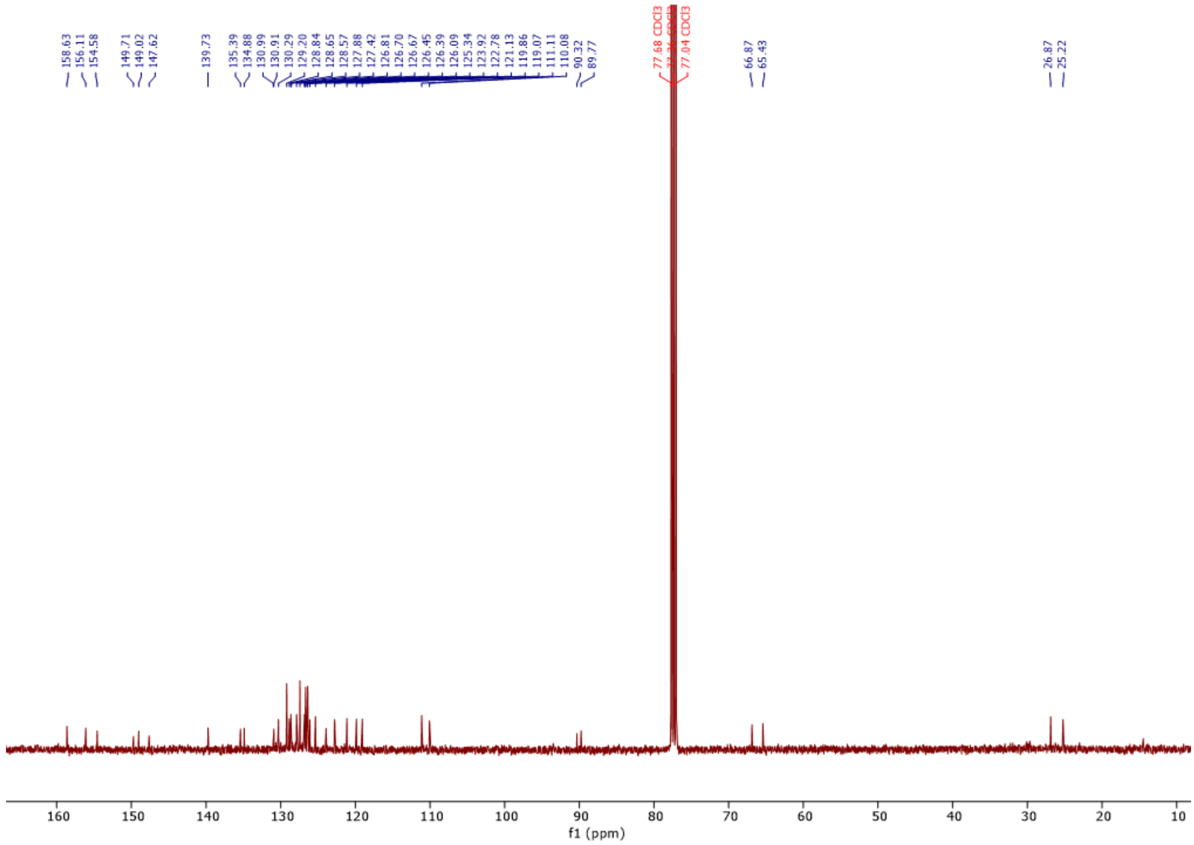

Figure S18. ^{13}C NMR (101 MHz) of **4-C4** in $CDCl_3$, measured at 298 K.

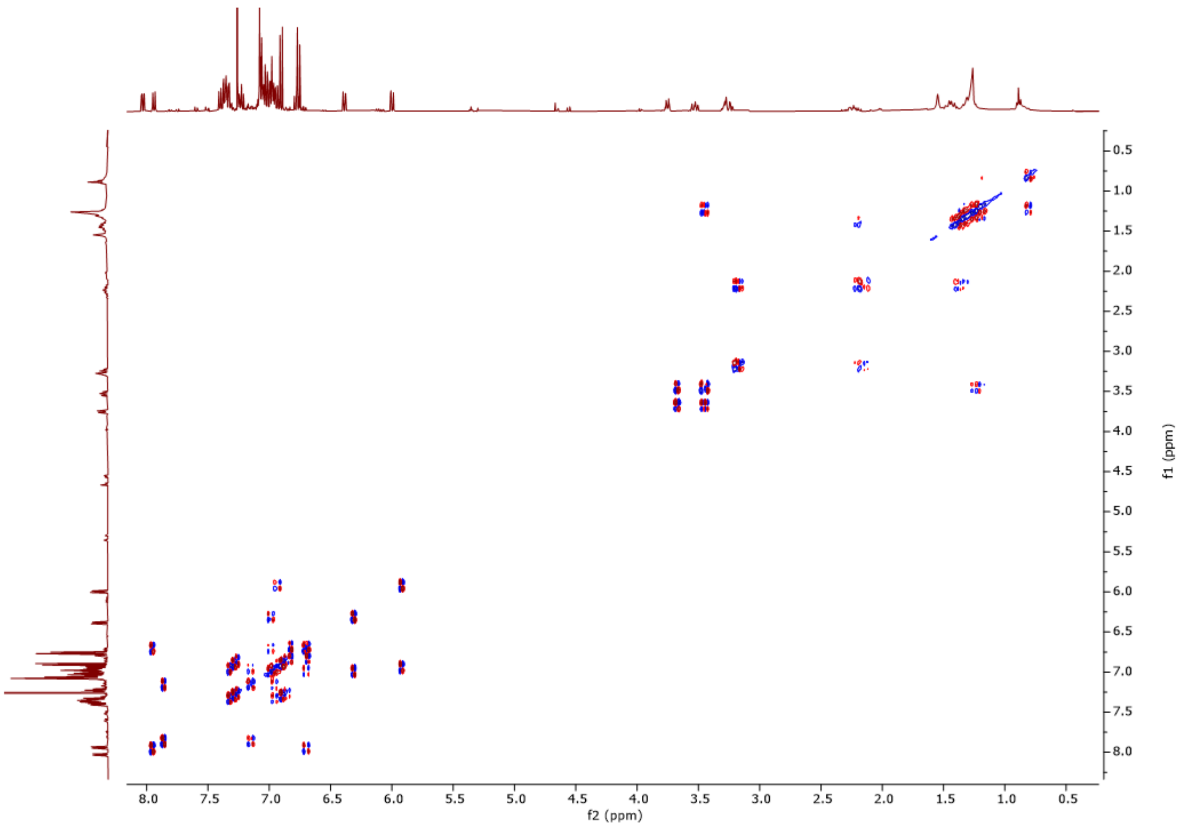

Figure S19. COSY NMR (400 MHz) of **4-C4** in $CDCl_3$, measured at 298 K.

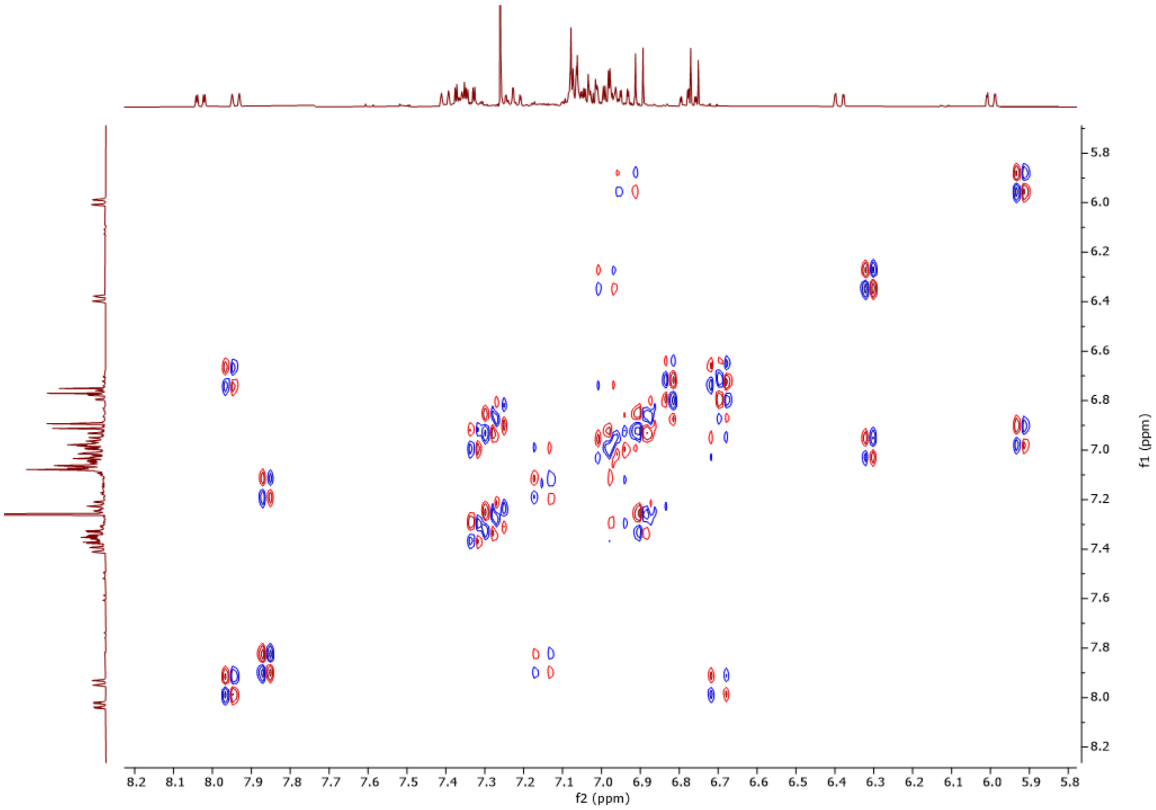

Figure S20. COSY NMR (400 MHz) of **4-C4** in $CDCl_3$, measured at 298 K (expansion in aromatic region).

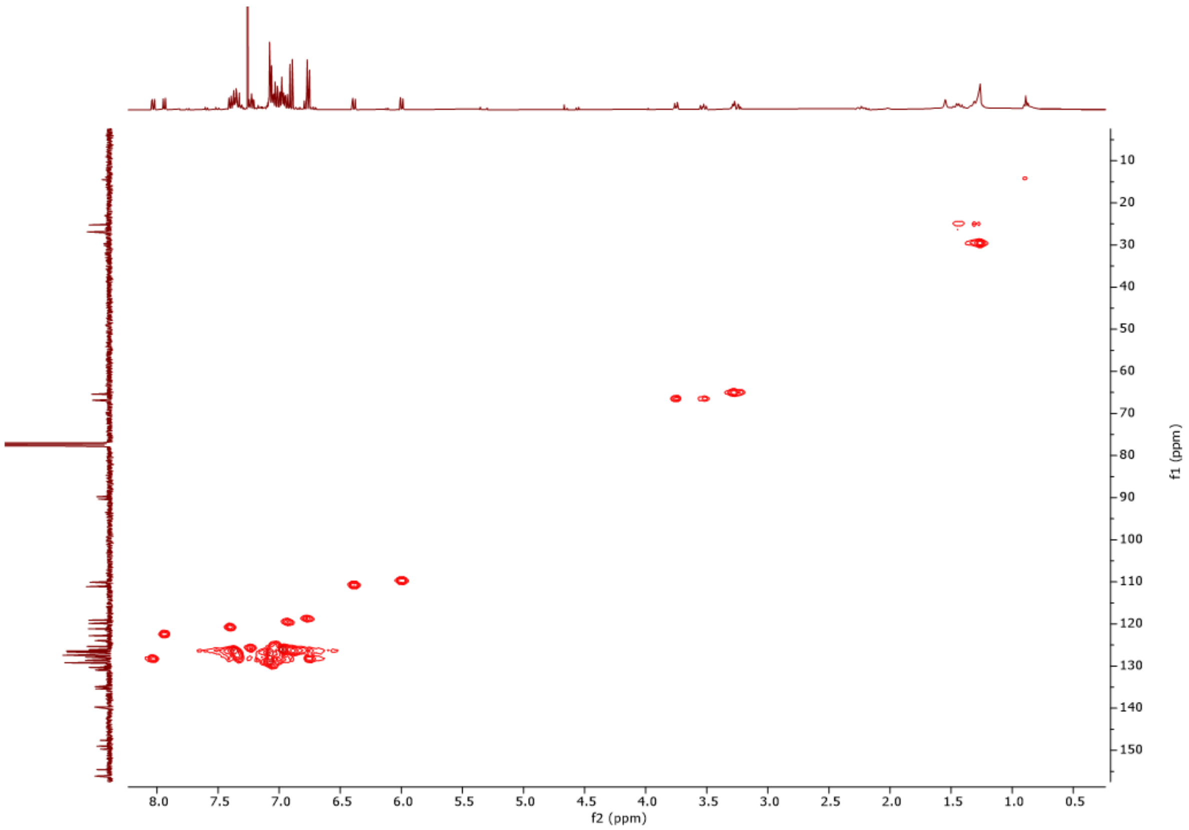

Figure S21. HSQC NMR (400 MHz) of **4-C4** in $CDCl_3$, measured at 298 K.

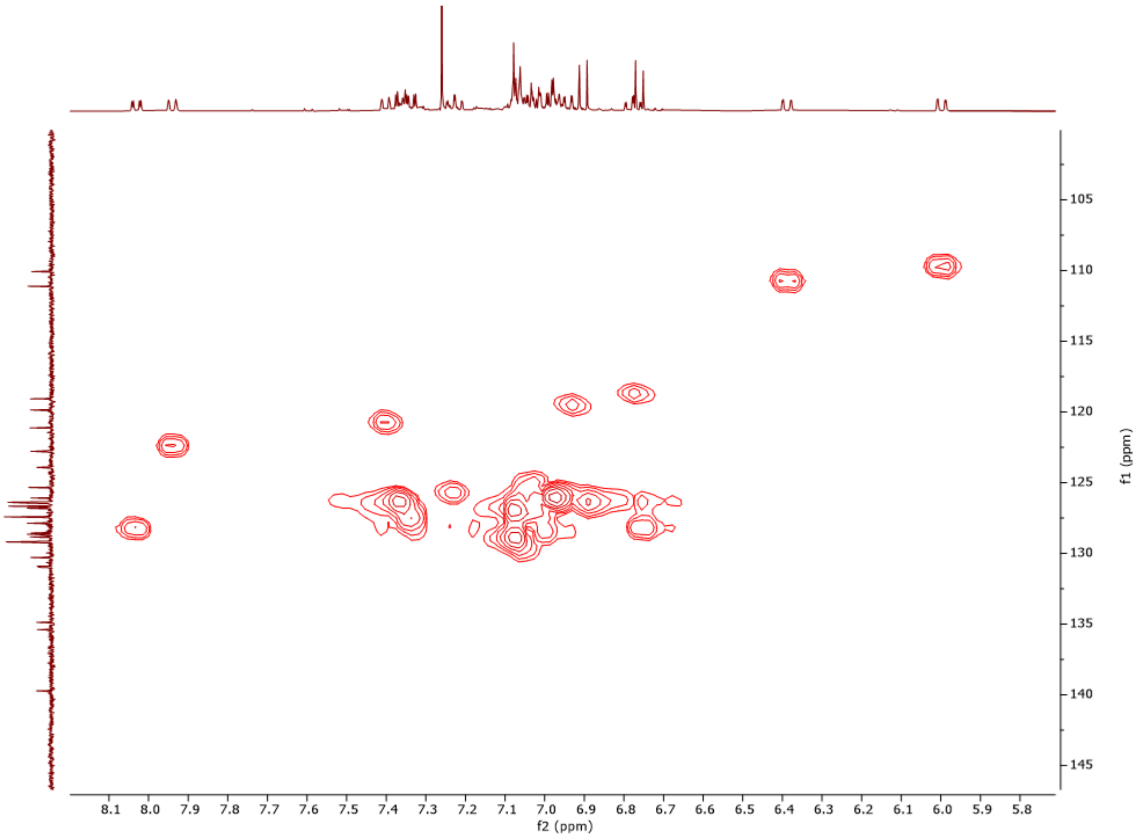

Figure S22. HSQC NMR (400 MHz) of **4-C4** in $CDCl_3$, measured at 298 K (expansion in aromatic region).

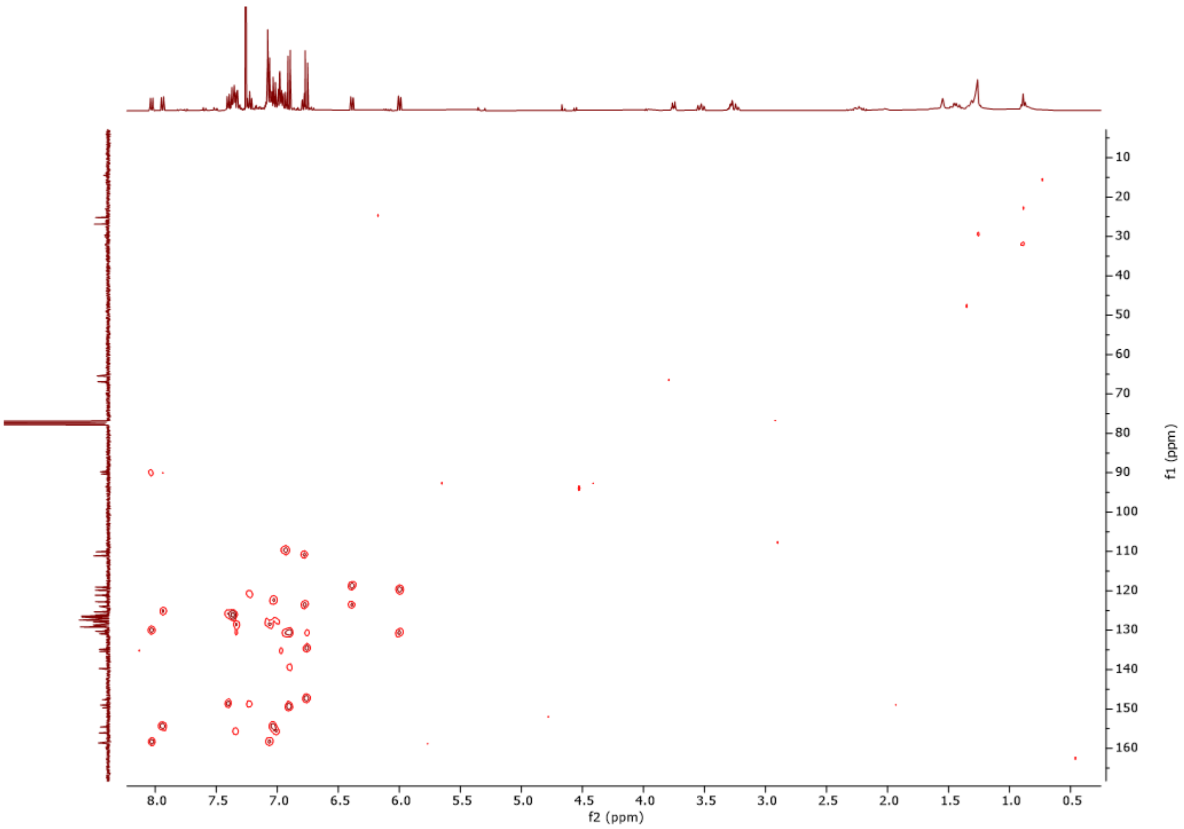

Figure S23. HMBC NMR (400 MHz) of **4-C4** in $CDCl_3$, measured at 298 K.

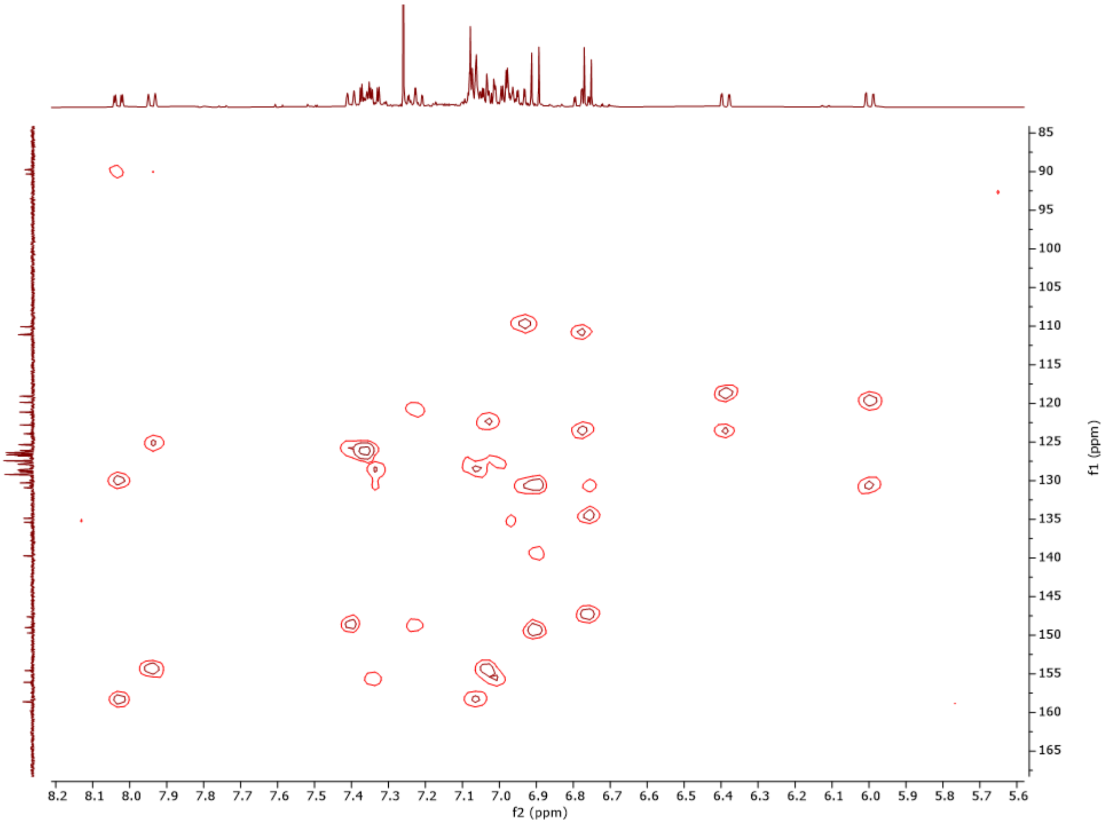

Figure S24. HMBC NMR (400 MHz) of **4-C4** in $CDCl_3$, measured at 298 K (expansion in aromatic region).

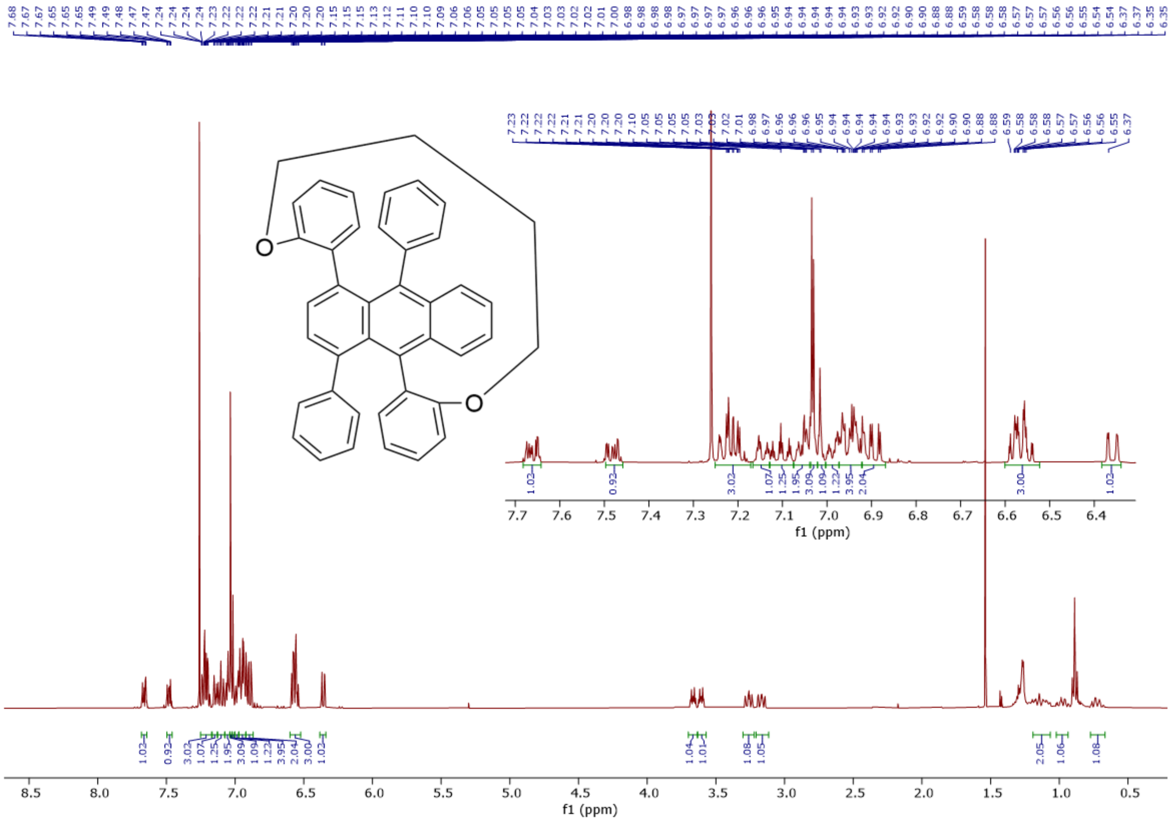

Figure S25. ^{1}H NMR (500 MHz) of **2-C4** in $CDCl_3$, measured at 298 K.

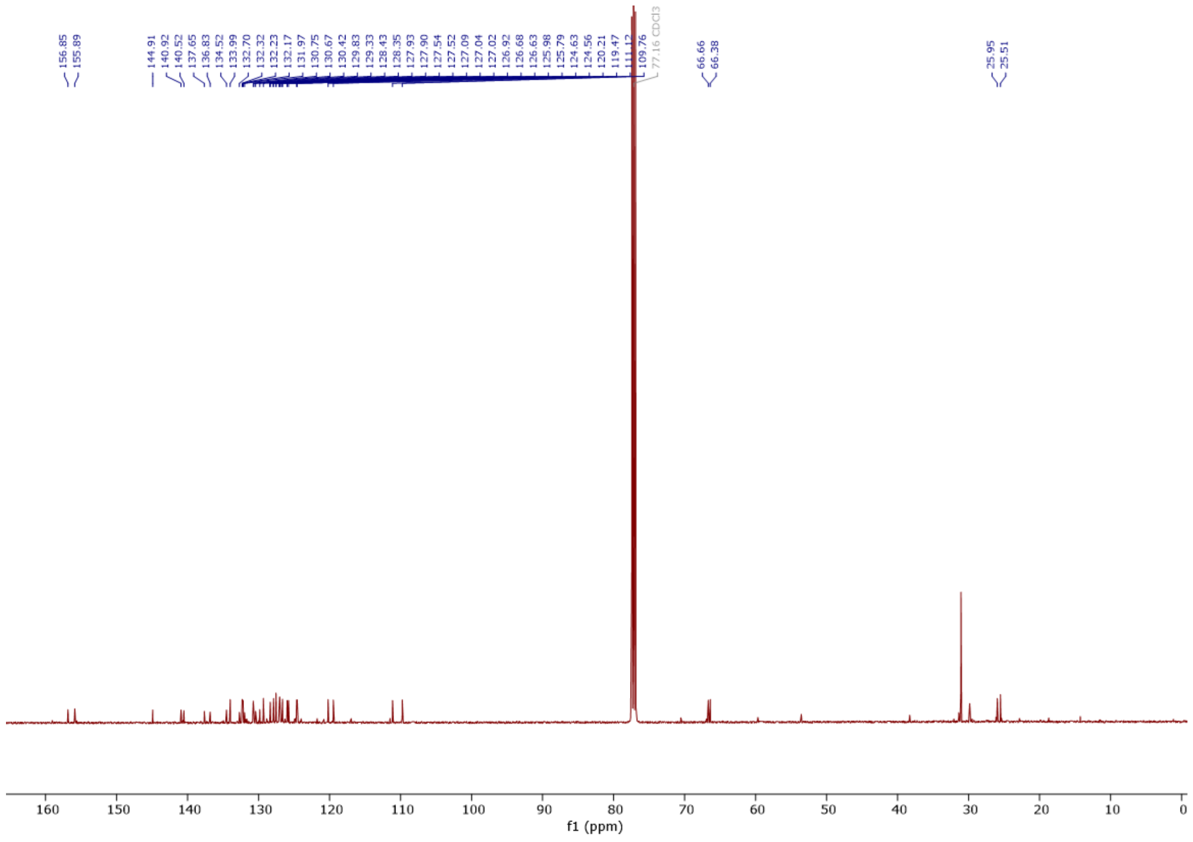

Figure S26. ^{13}C NMR (126 MHz) of **2-C4** in $CDCl_3$, measured at 298 K.

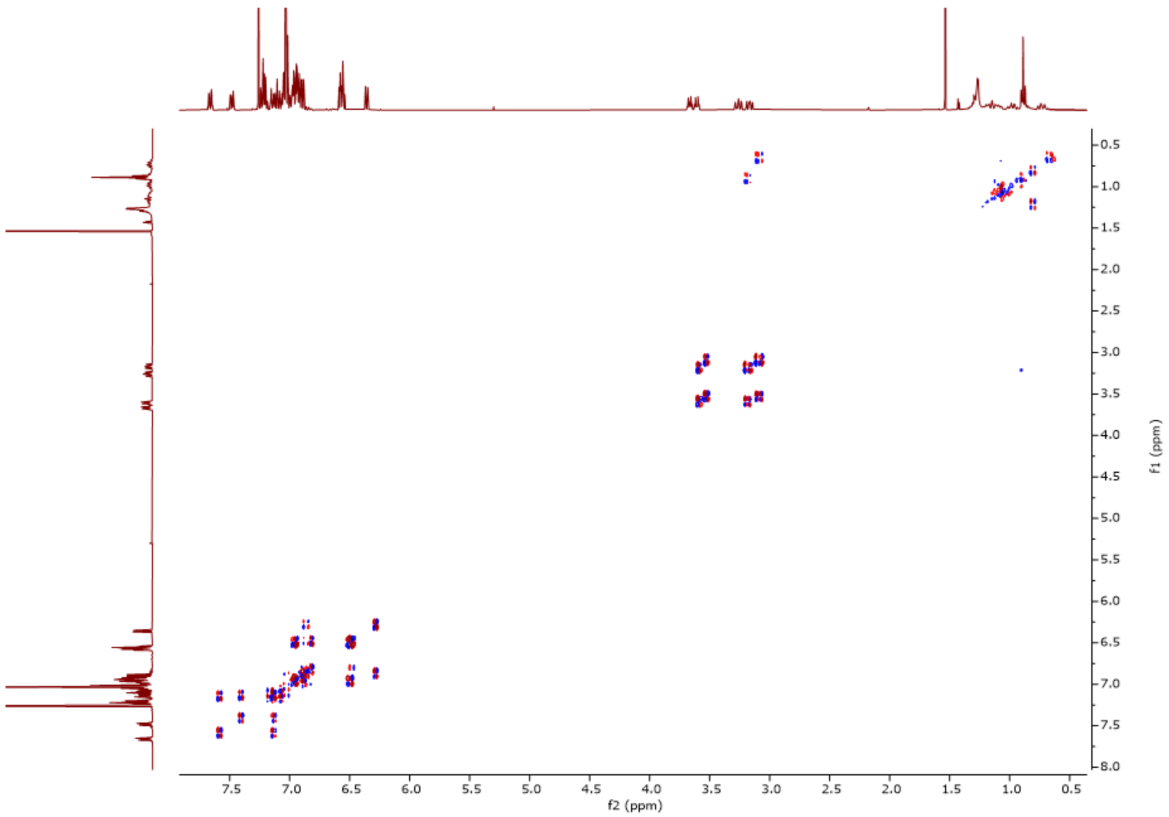

Figure S27. COSY NMR (500 MHz) of **2-C4** in $CDCl_3$, measured at 298 K.

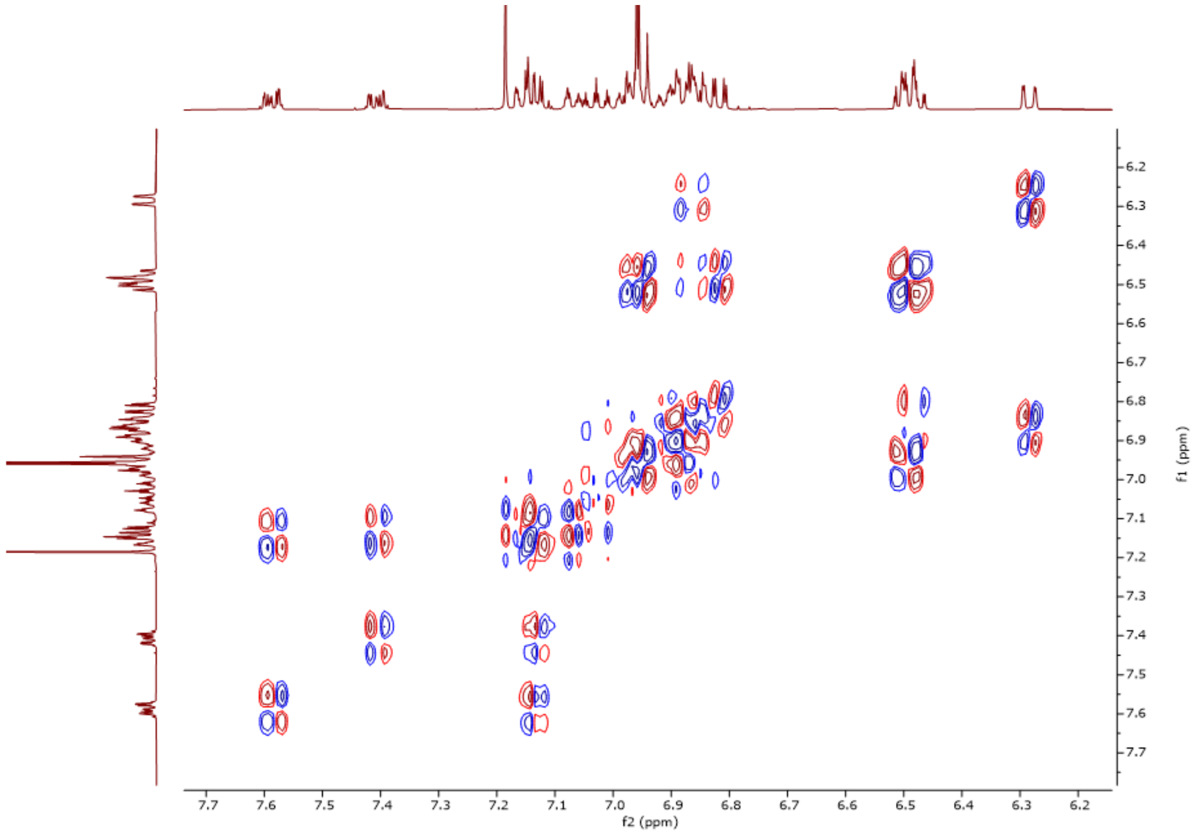

Figure S28. COSY NMR (500 MHz) of **2-C4** in $CDCl_3$, measured at 298 K (expansion in aromatic region).

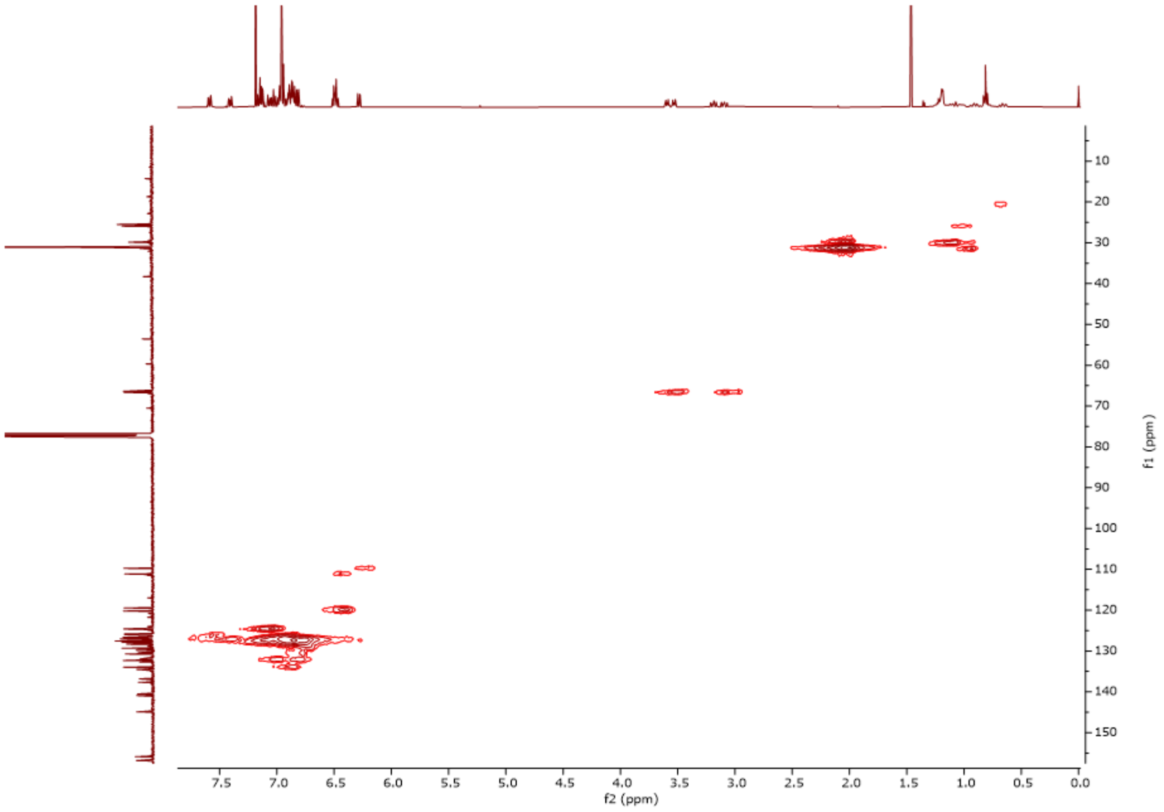

Figure S29. HSQC NMR (400 MHz) of **2-C4** in $CDCl_3$, measured at 298 K.

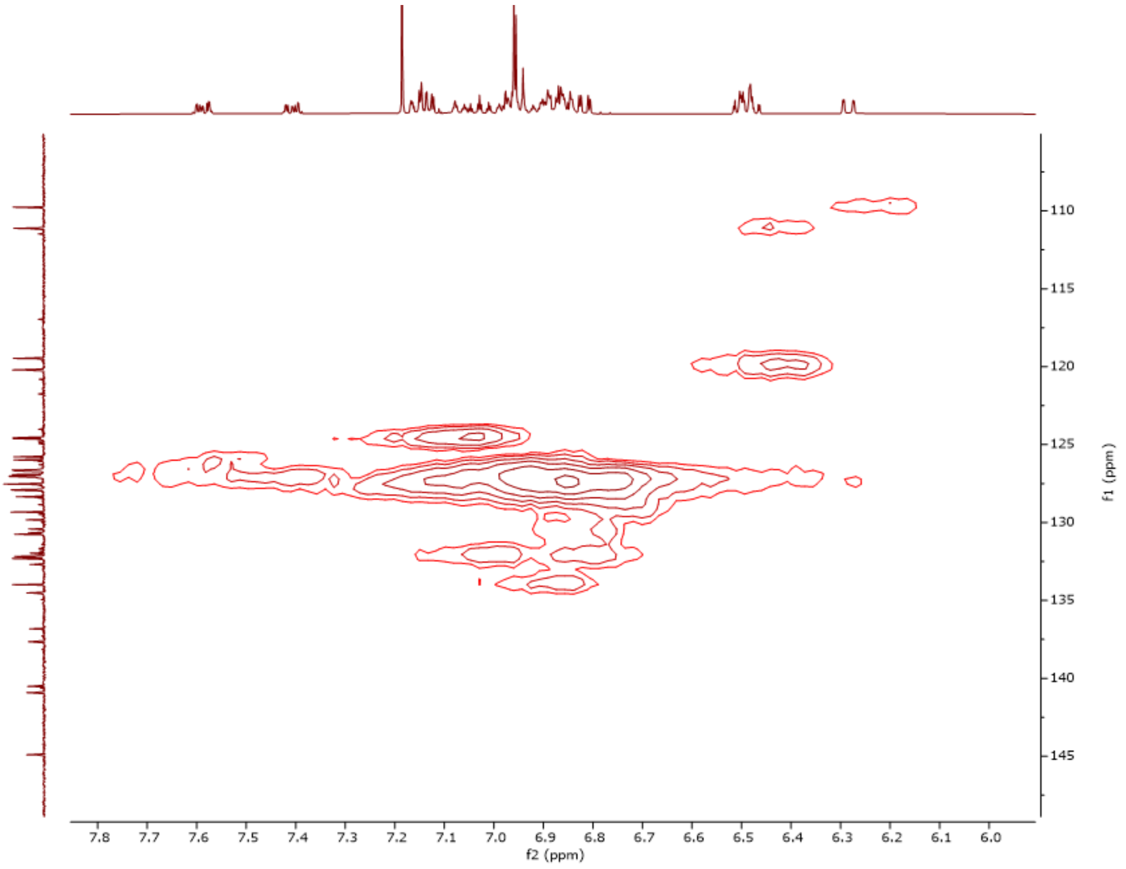

Figure S30. HSQC NMR (400 MHz) of **2-C4** in $CDCl_3$, measured at 298 K (expansion in aromatic region).

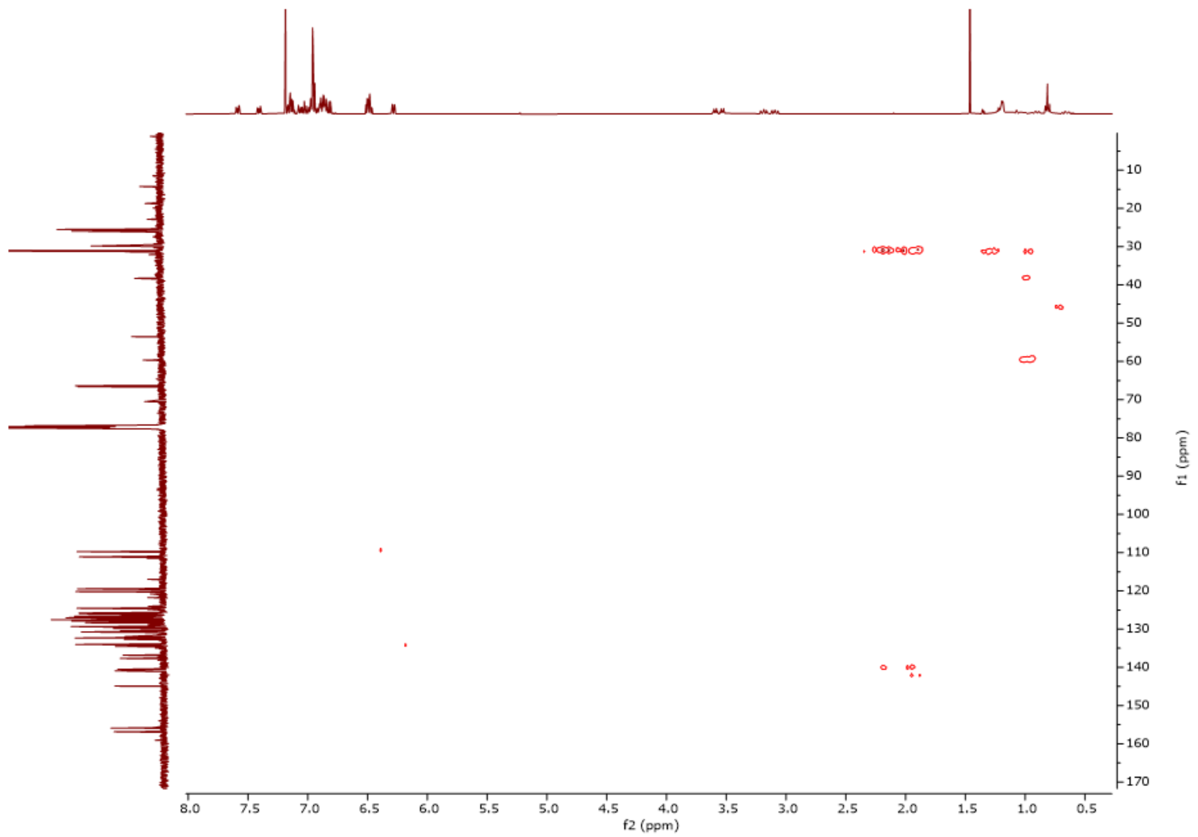

Figure S31. HMBC NMR (400 MHz) of **2-C4** in $CDCl_3$, measured at 298 K.

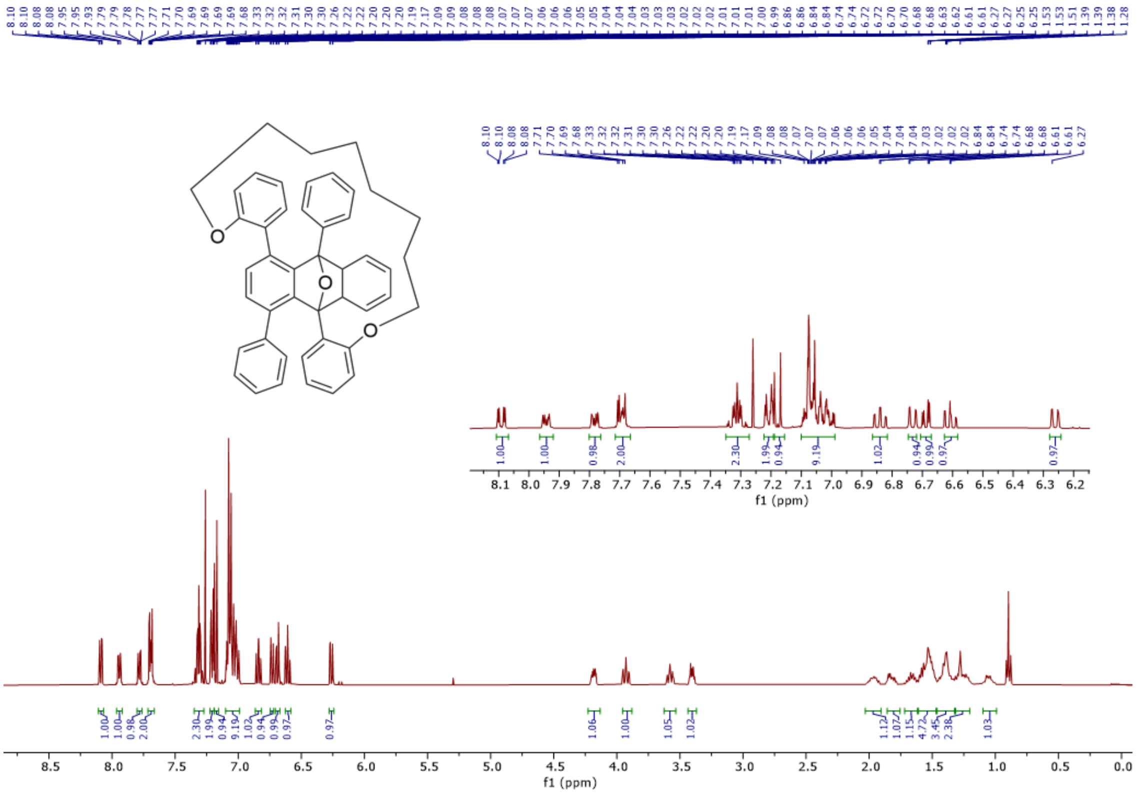

Figure S32. ^{1}H NMR (400 MHz) of **4-C8** in $CDCl_3$, measured at 298 K.

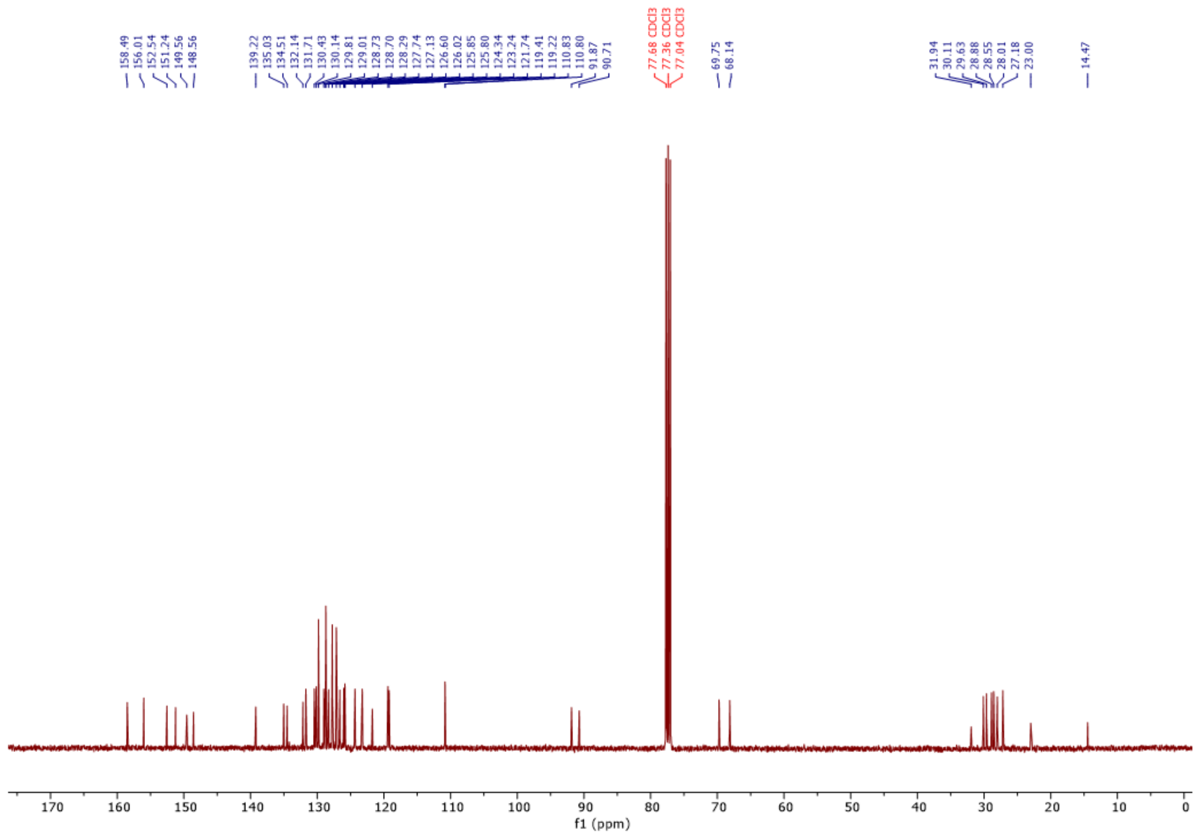

Figure S33. ^{13}C NMR (101 MHz) of **4-C8** in $CDCl_3$, measured at 298 K.

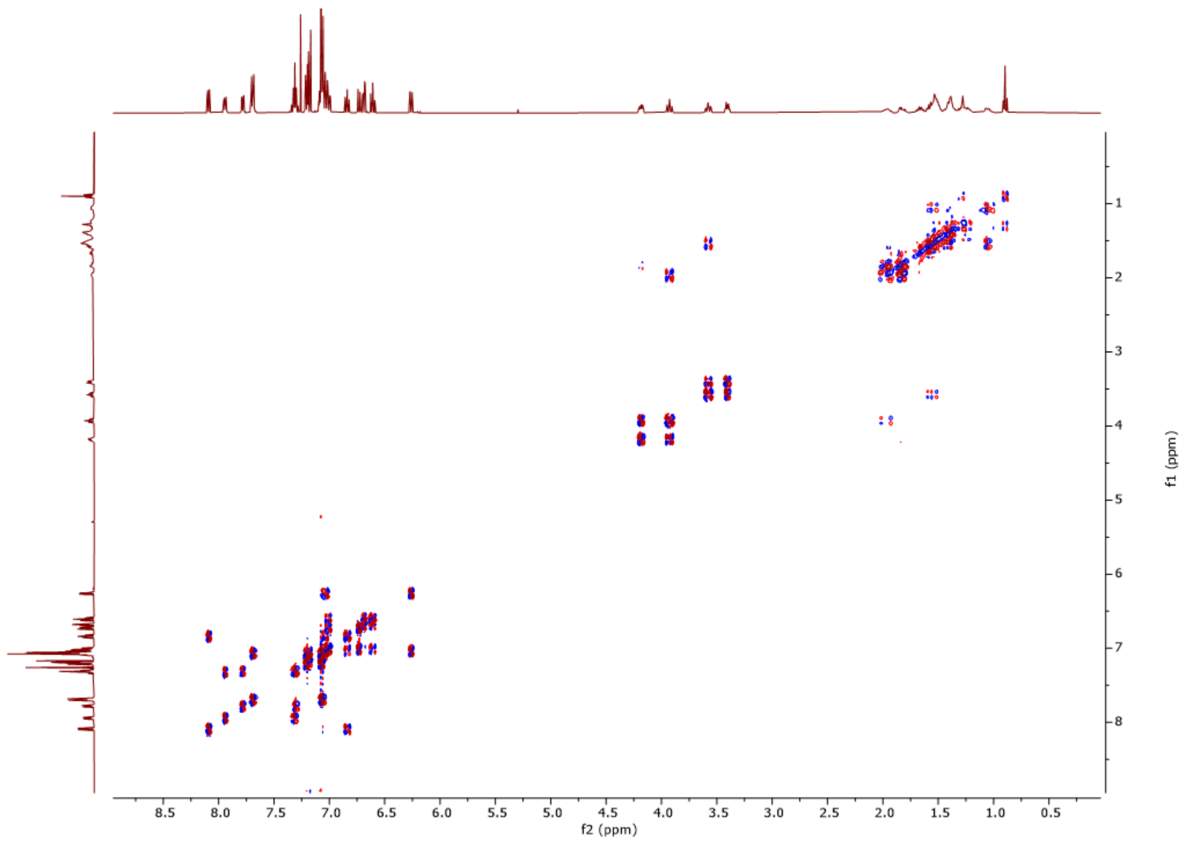

Figure S34. COSY NMR (400 MHz) of **4-C8** in $CDCl_3$, measured at 298 K.

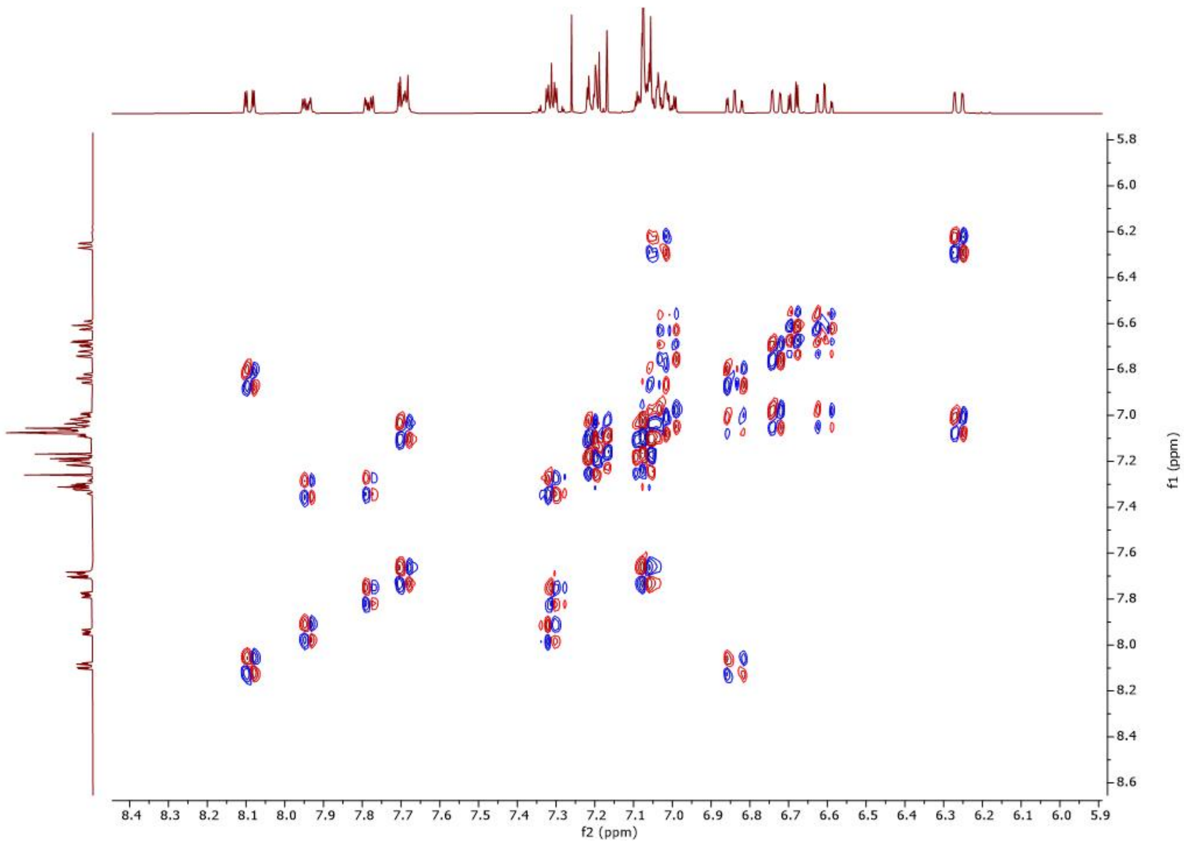

Figure S35. COSY NMR (400 MHz) of **4-C8** in $CDCl_3$, measured at 298 K (expansion in aromatic region).

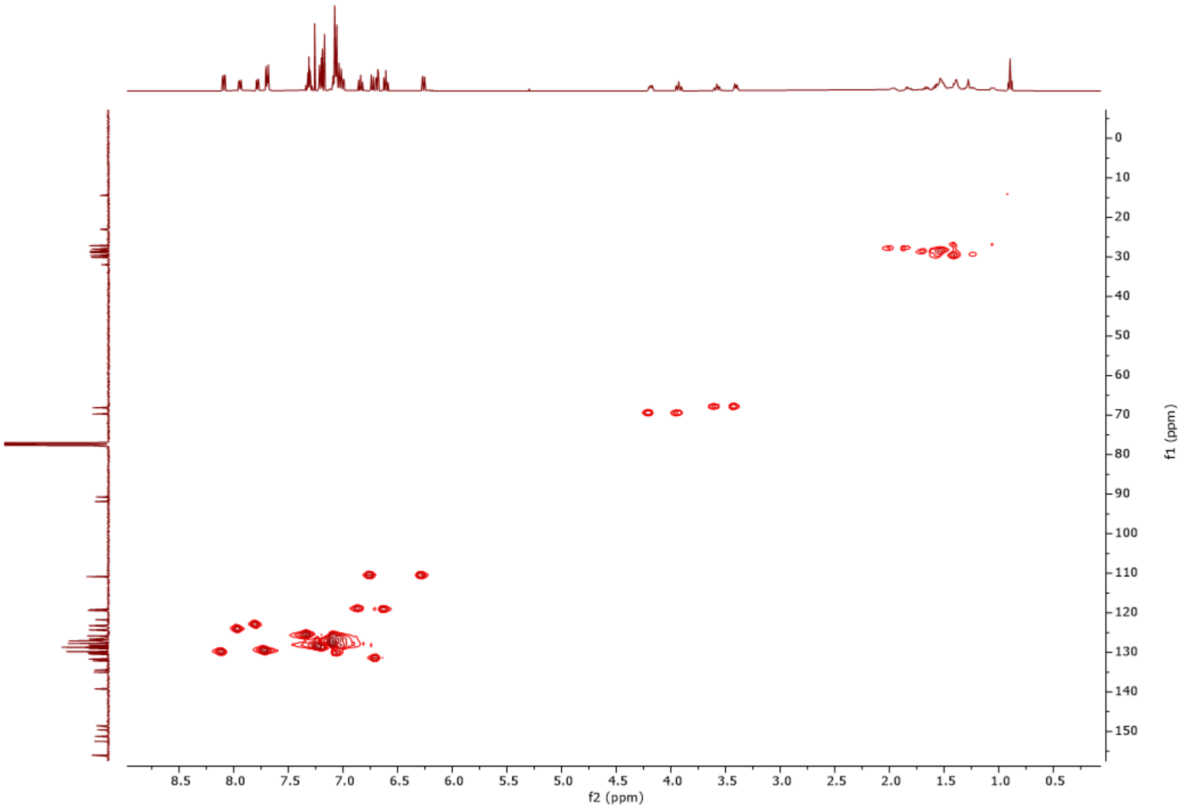

Figure S36. HSQC NMR (400 MHz) of **4-C8** in $CDCl_3$, measured at 298 K.

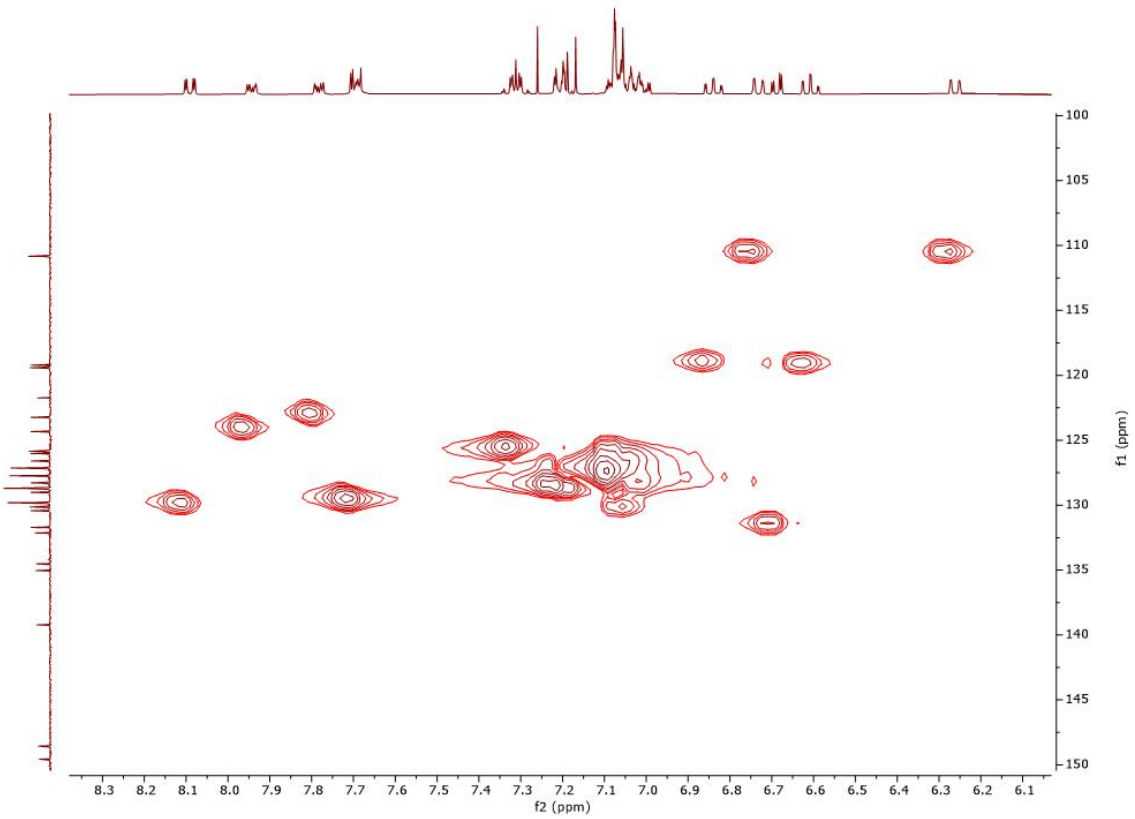

Figure S37. HSQC NMR (400 MHz) of **4-C8** in $CDCl_3$, measured at 298 K (expansion in aromatic region).

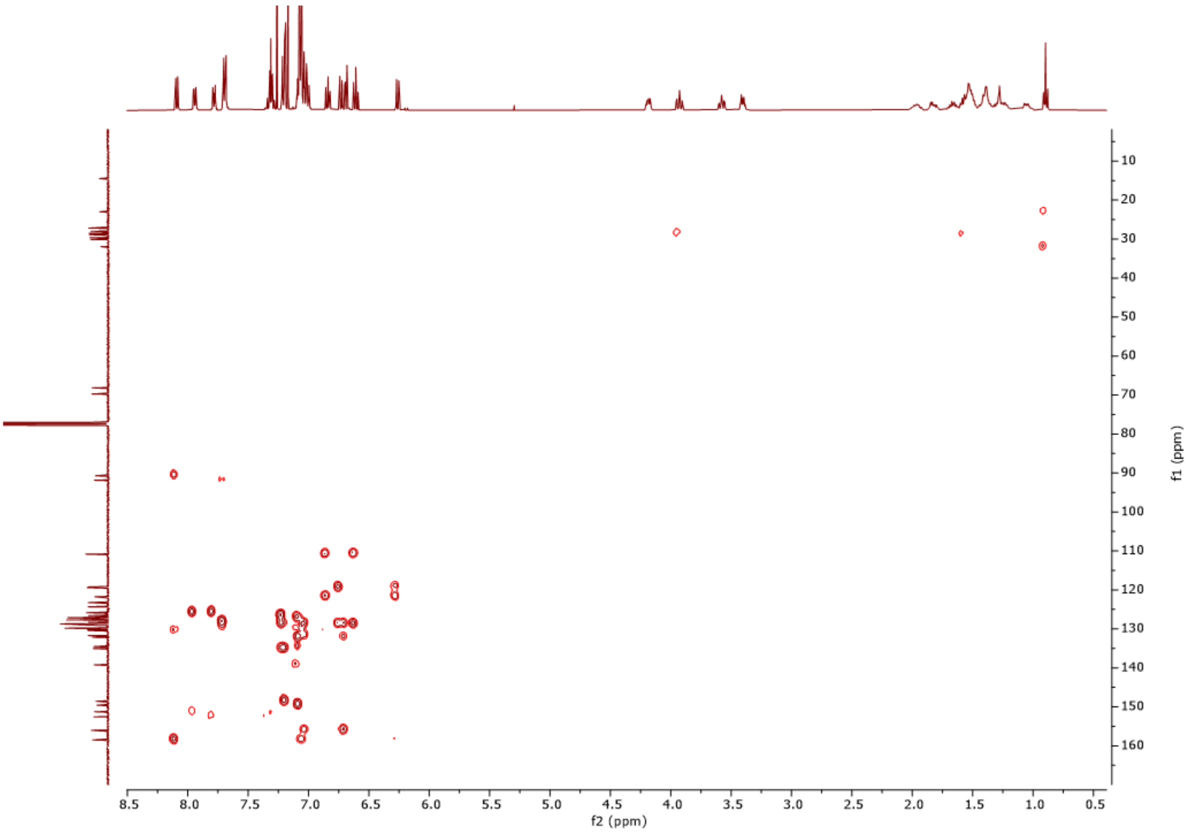

Figure S38. HMBC NMR (400 MHz) of **4-C8** in $CDCl_3$, measured at 298 K.

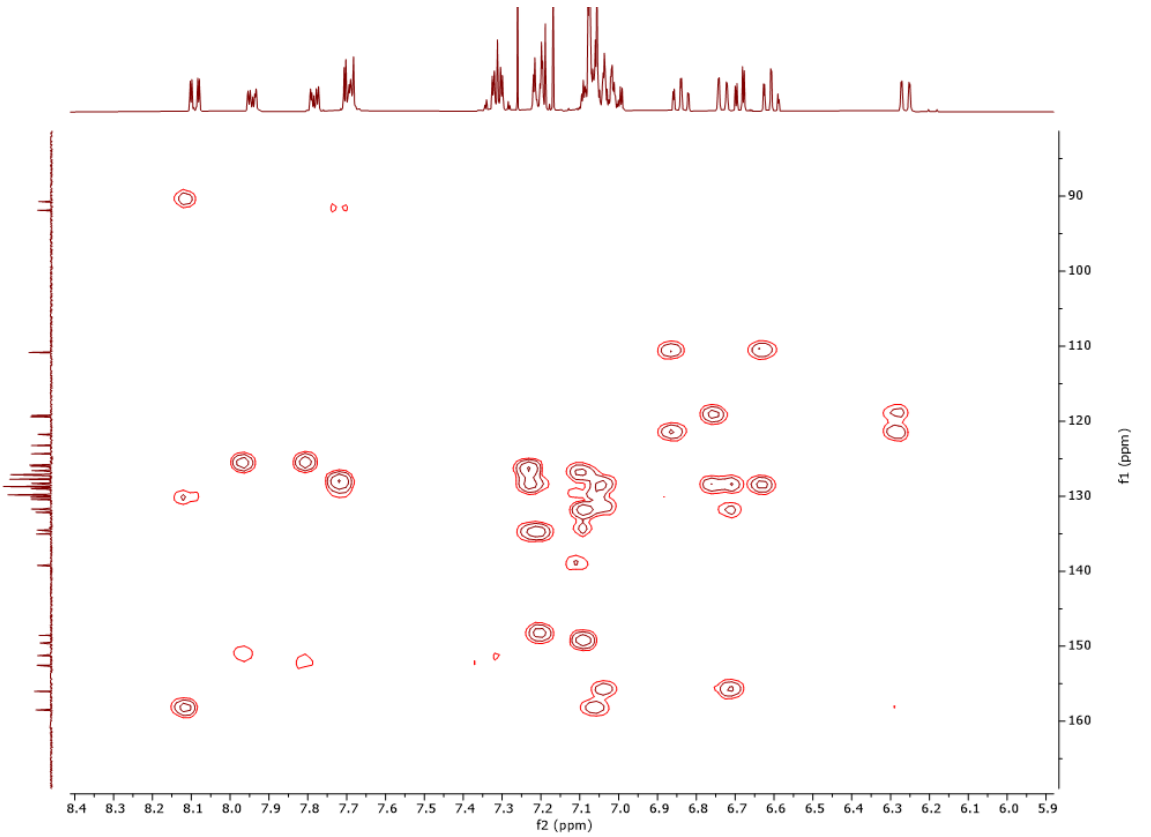

Figure S39. HMBC NMR (400 MHz) of **4-C8** in $CDCl_3$, measured at 298 K (expansion in aromatic region).

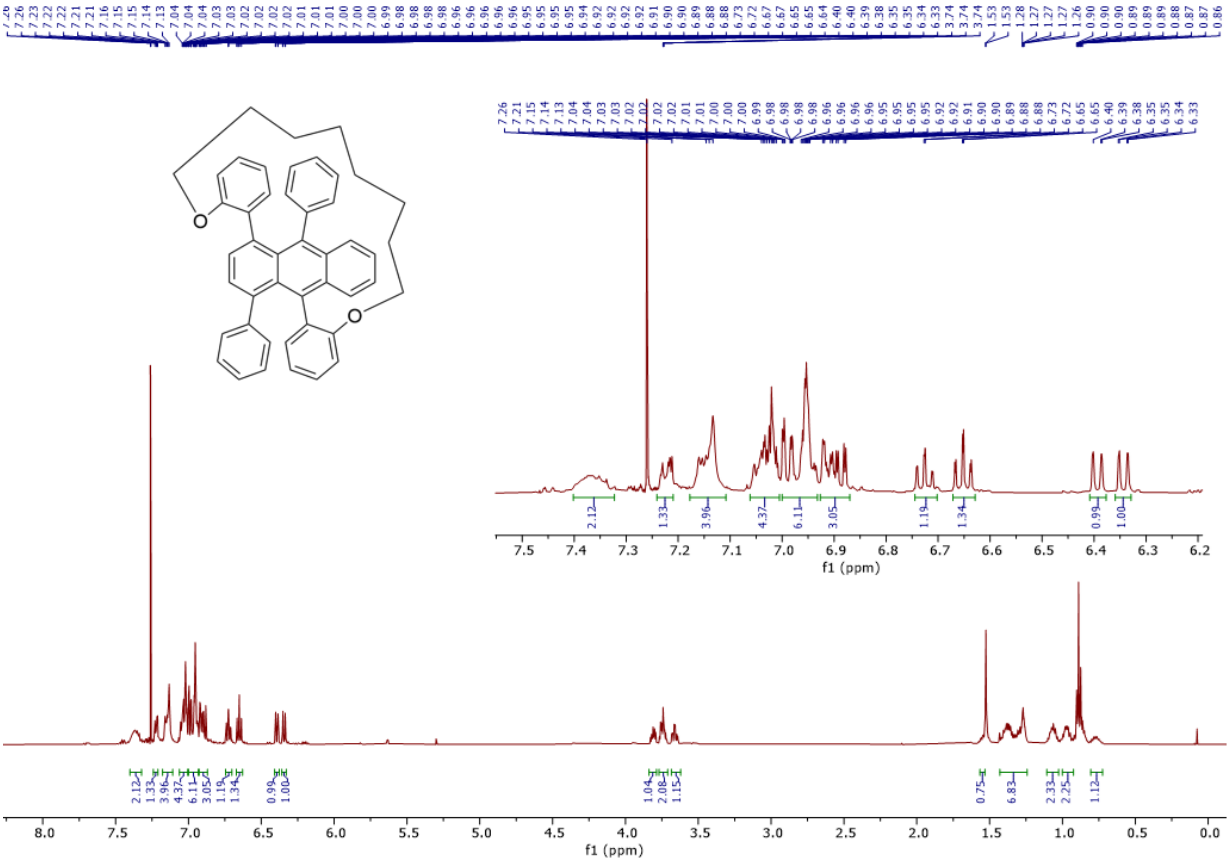

Figure S40. ^{1}H NMR (400 MHz) of **2-C8** in $CDCl_3$, measured at 298 K.

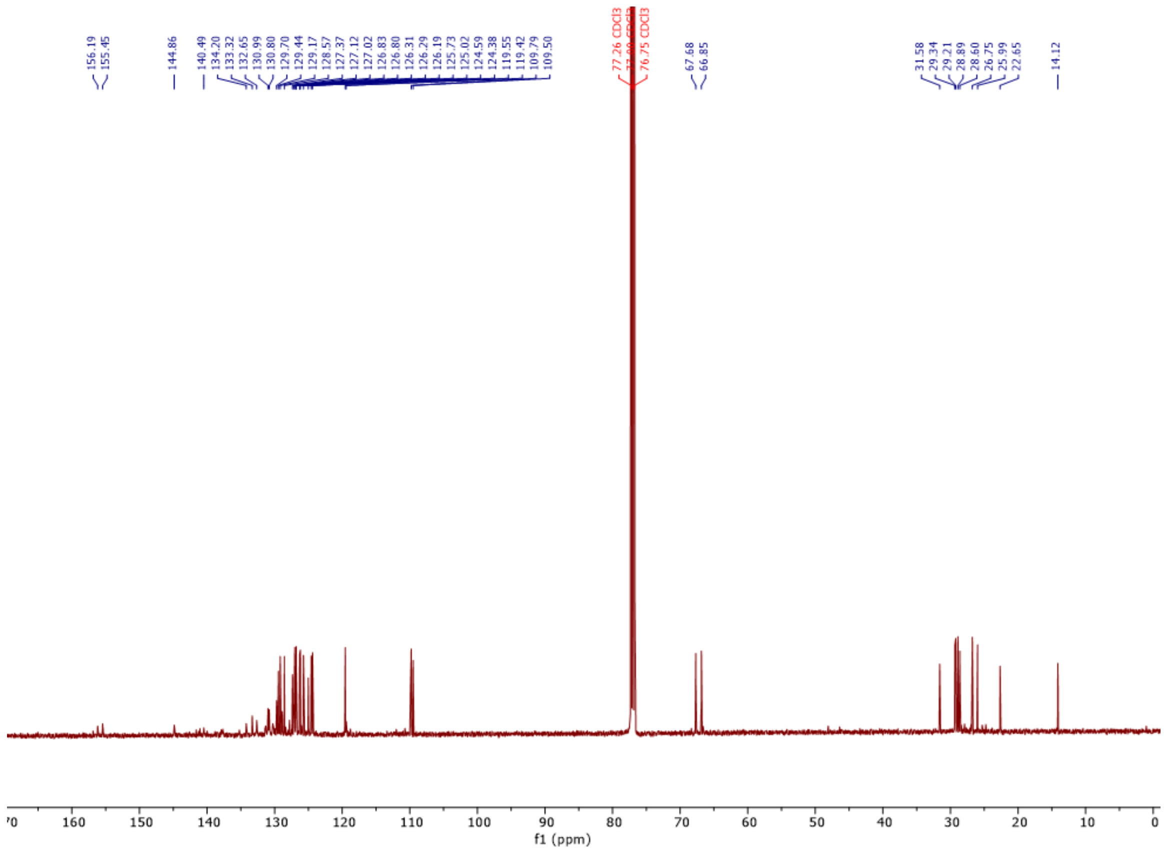

Figure S41. ^{13}C NMR (101 MHz) of **2-C8** in $CDCl_3$, measured at 298 K.

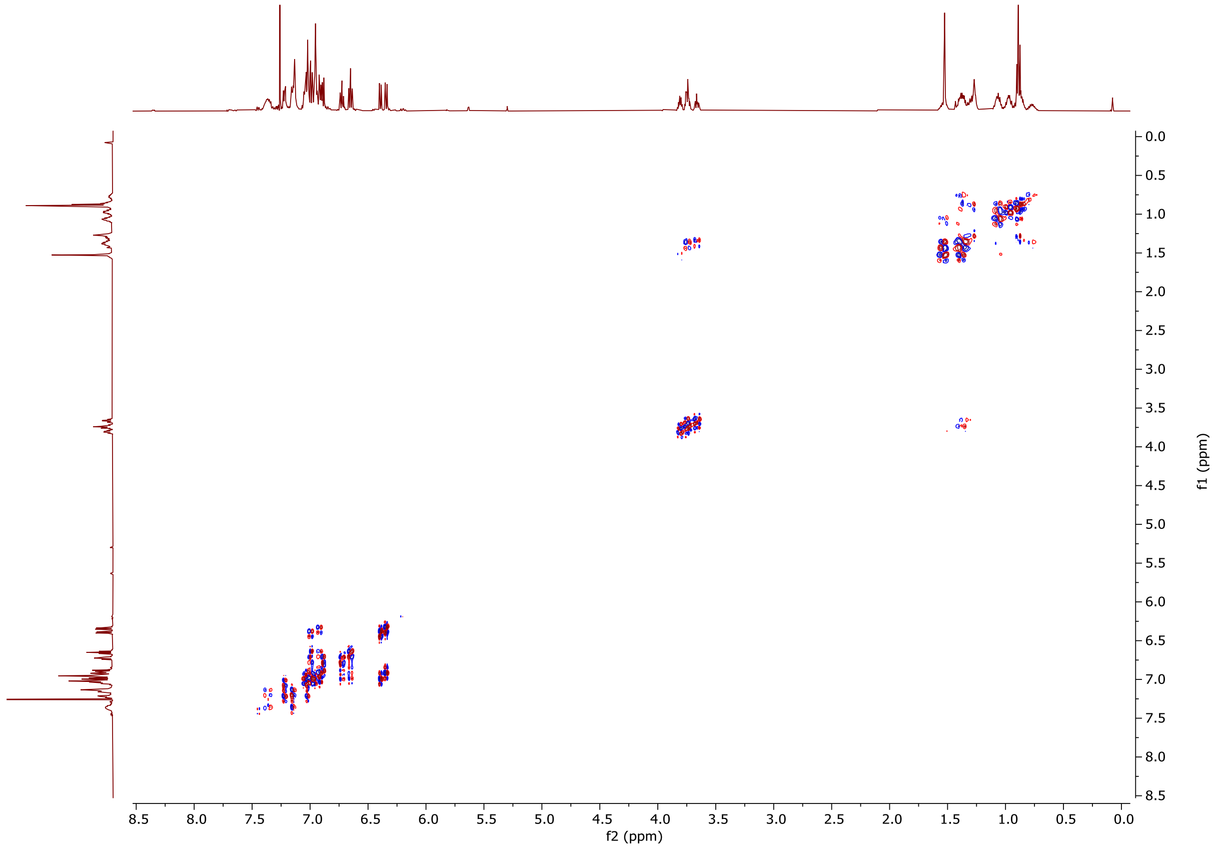

Figure S42. COSY NMR (400 MHz) of **2-C8** in $CDCl_3$, measured at 298 K.

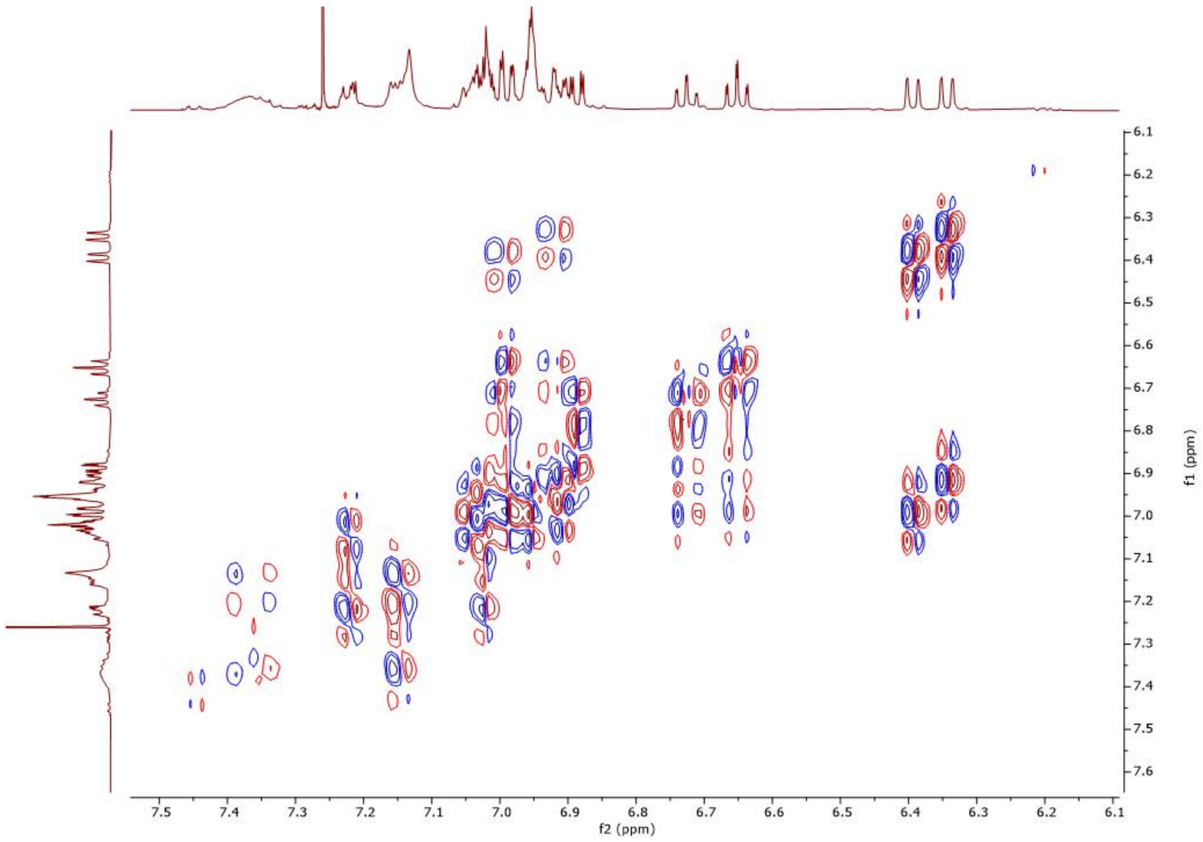

Figure S43. COSY NMR (400 MHz) of **2-C8** in $CDCl_3$, measured at 298 K (expansion in aromatic region).

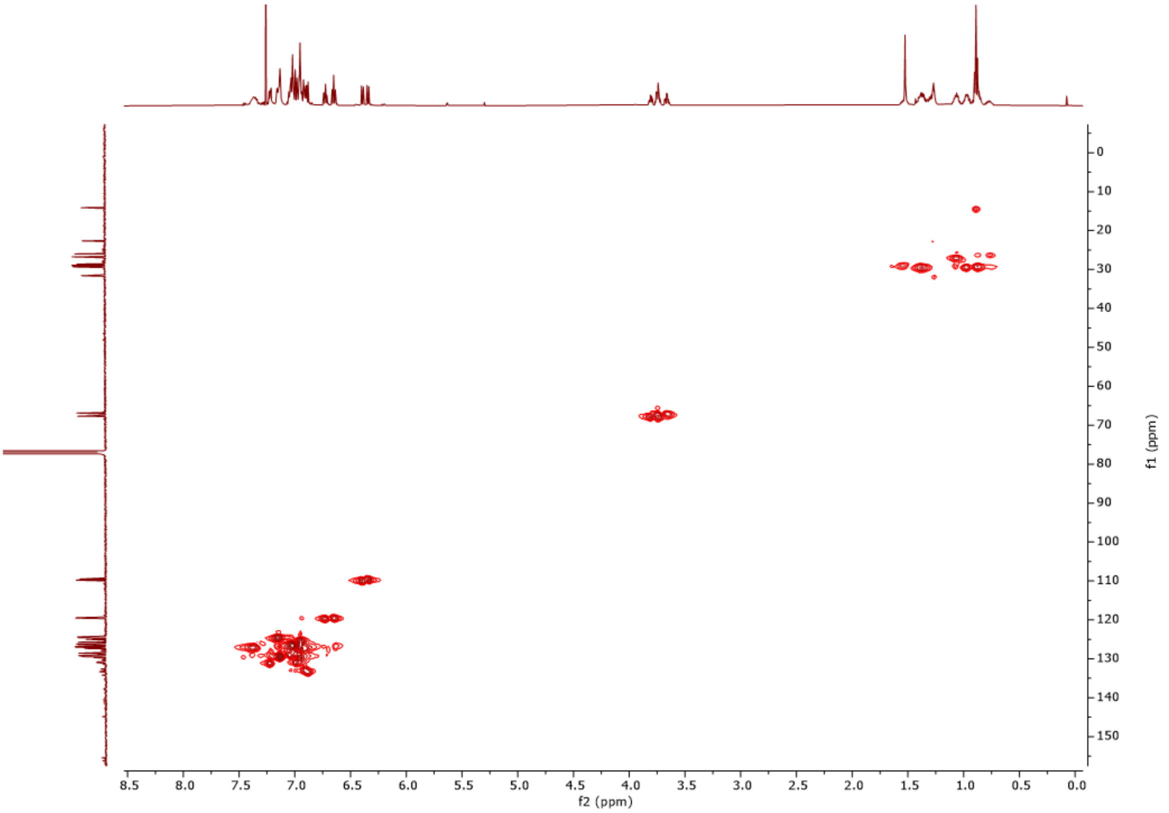

Figure S44. HSQC NMR (400 MHz) of **2-C8** in $CDCl_3$, measured at 298 K.

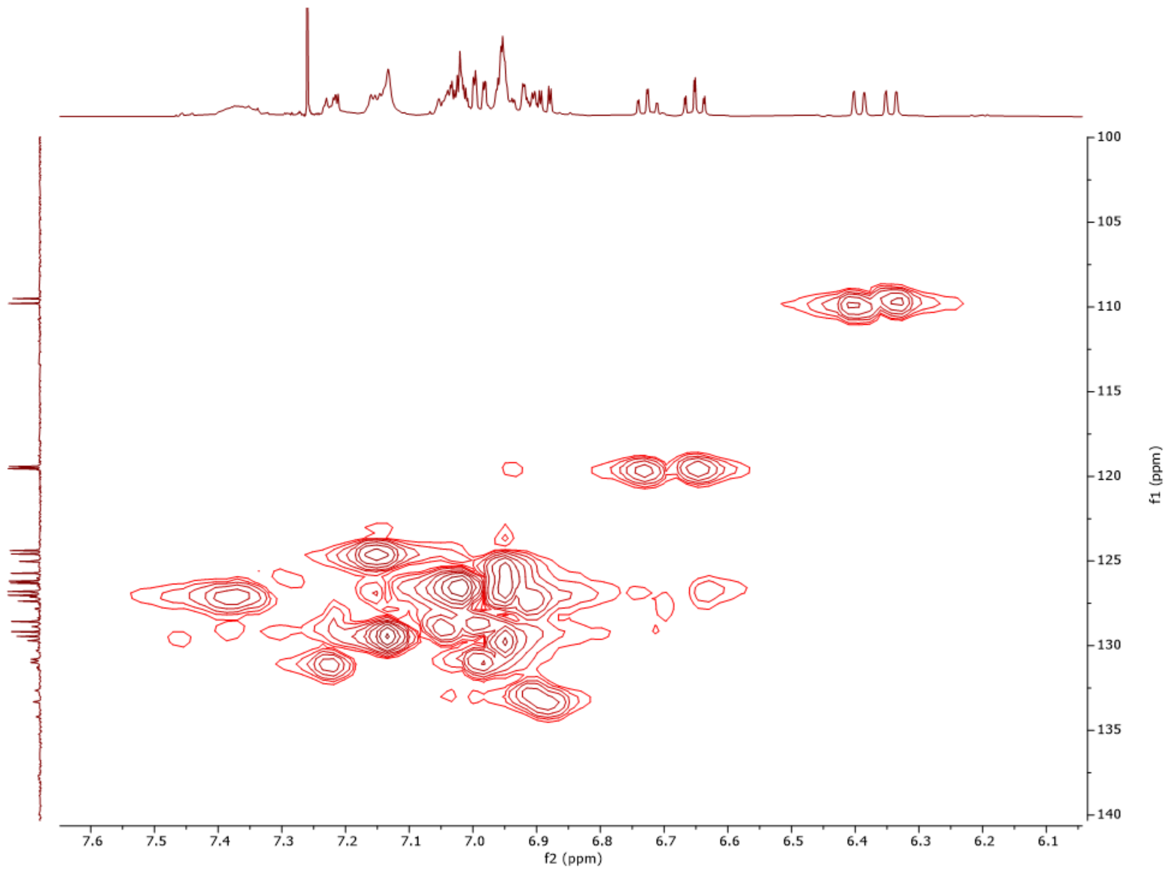

Figure S45. HSQC NMR (400 MHz) of **2-C8** in $CDCl_3$, measured at 298 K (expansion in aromatic region).

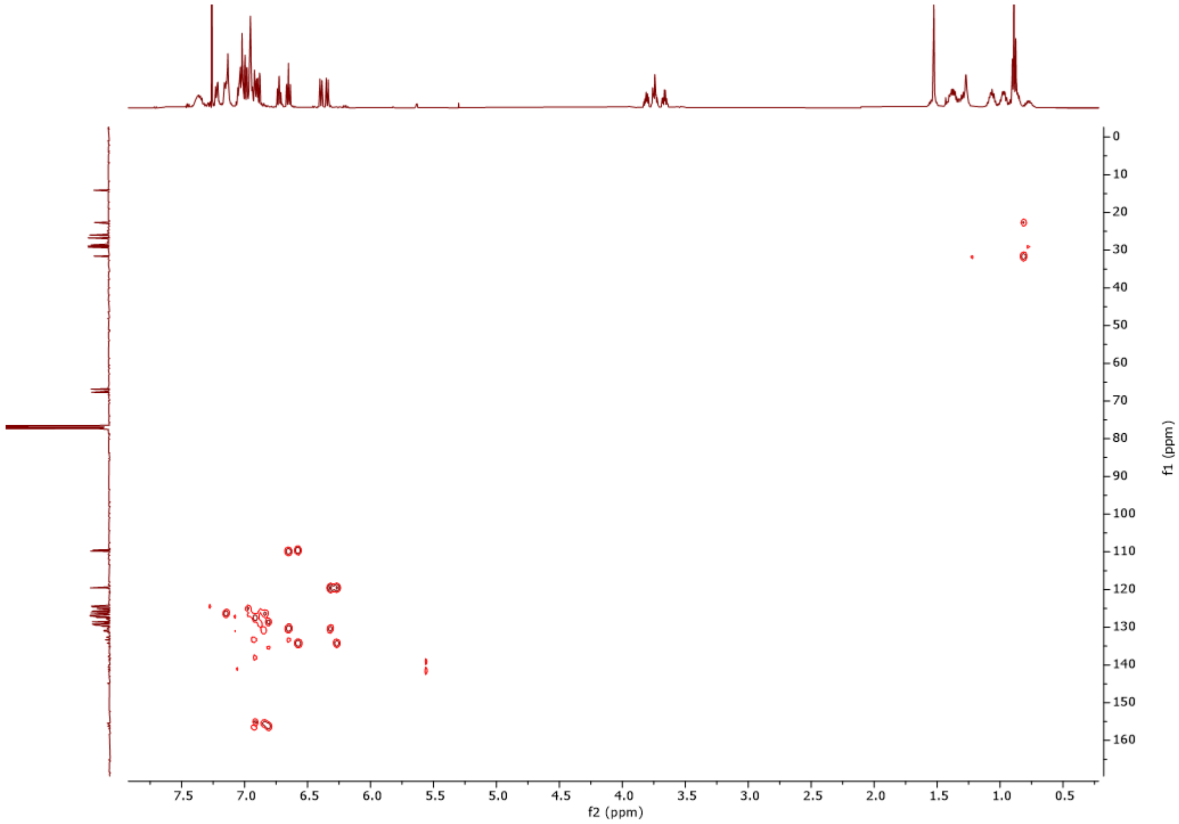

Figure S46. HMBC NMR (400 MHz) of **2-C8** in $CDCl_3$, measured at 298 K.

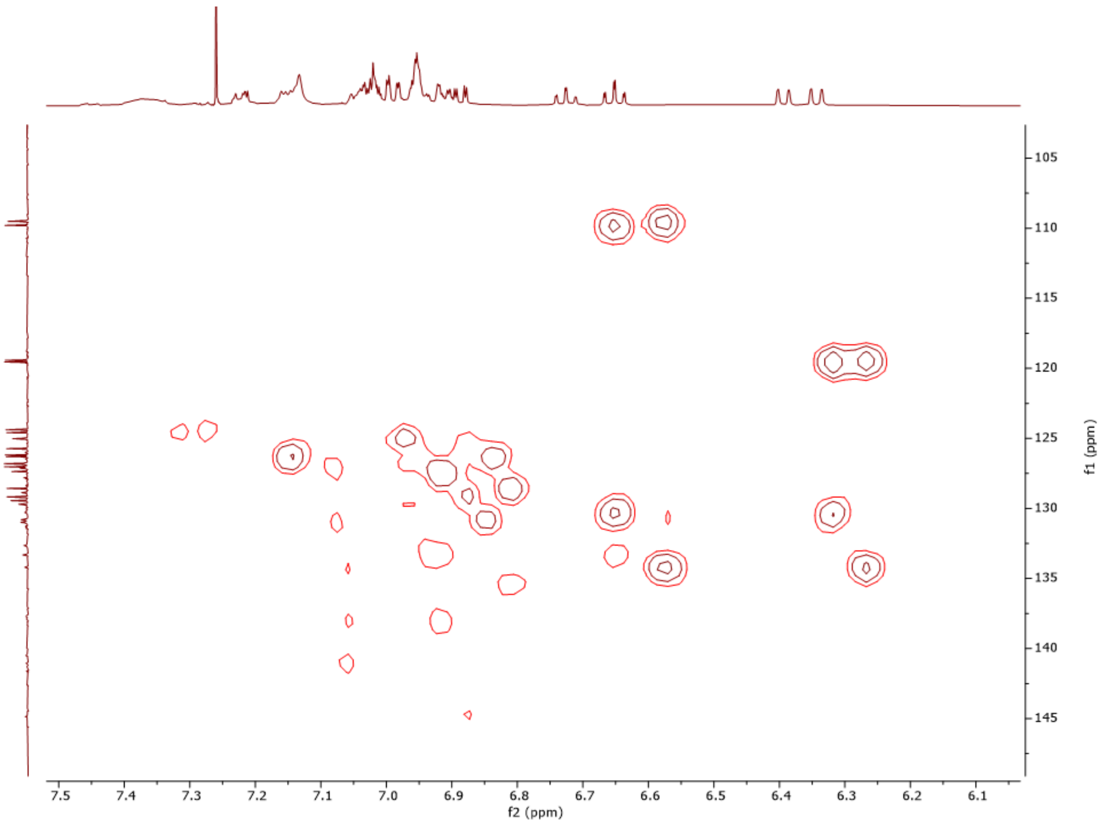

Figure S47. HMBC (400 MHz) of **2-C8** in $CDCl_3$, measured at 298 K (expansion in aromatic region).

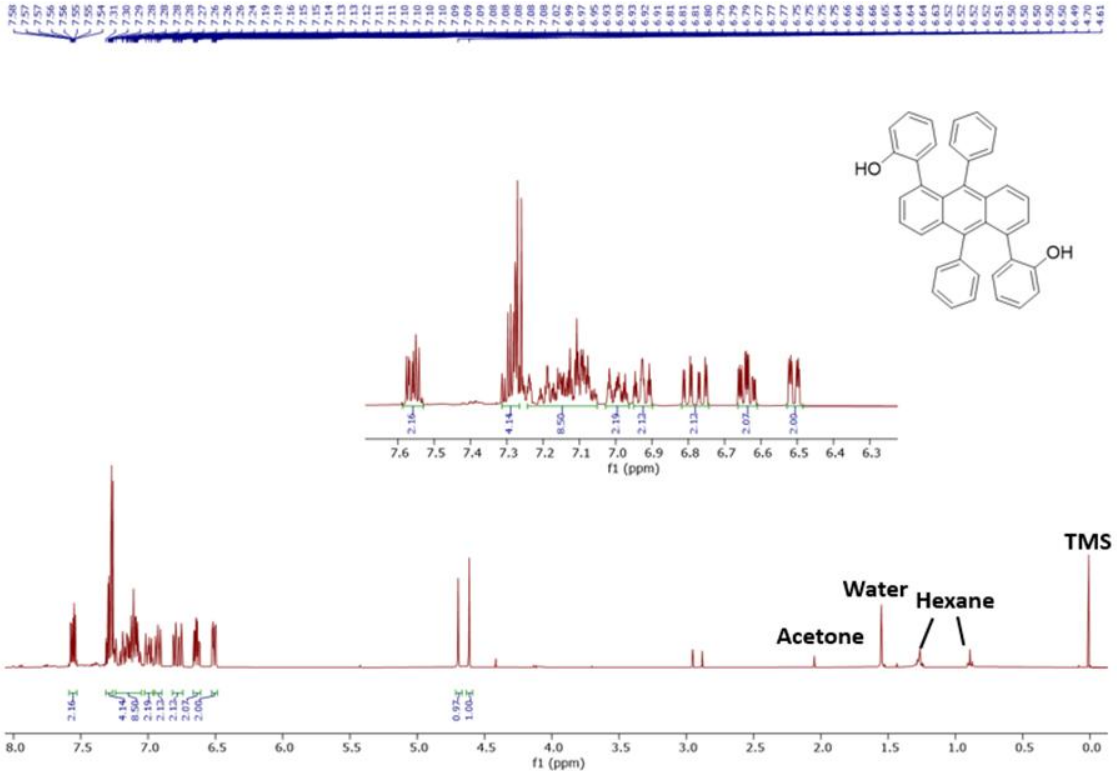

Figure S48. ^{1}H NMR (400 MHz) of *syn*-**6** and *anti*-**6** atropisomers in $CDCl_3$, measured at 298 K.

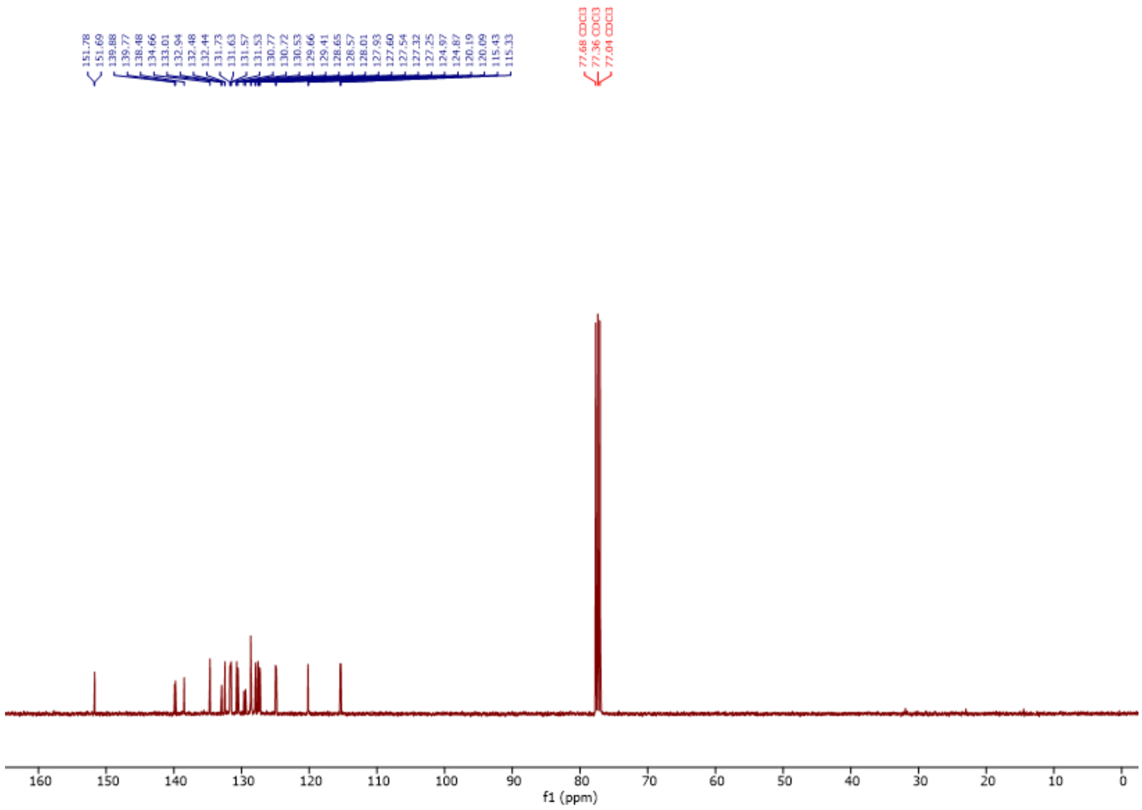

Figure S49. ^{13}C NMR (400 MHz) of **6** in $CDCl_3$, measured at 298 K.

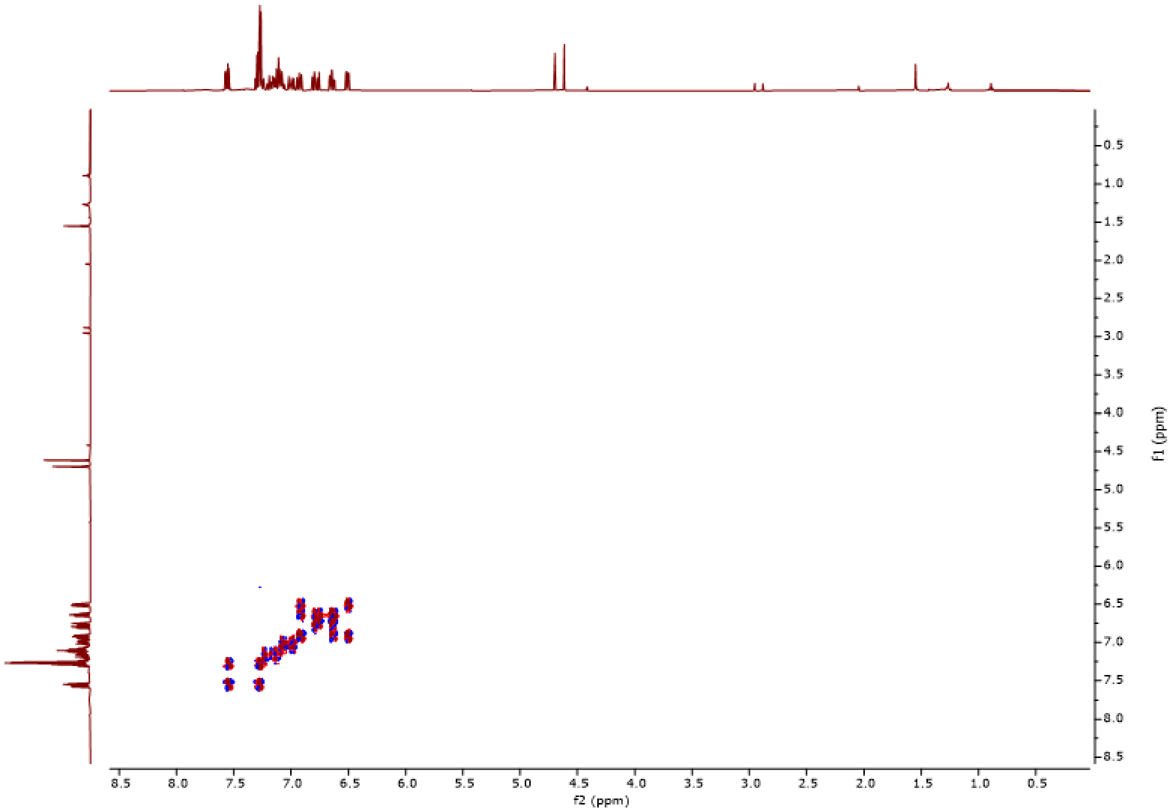

Figure S50. COSY NMR (400 MHz) of **6** in $CDCl_3$, measured at 298 K.

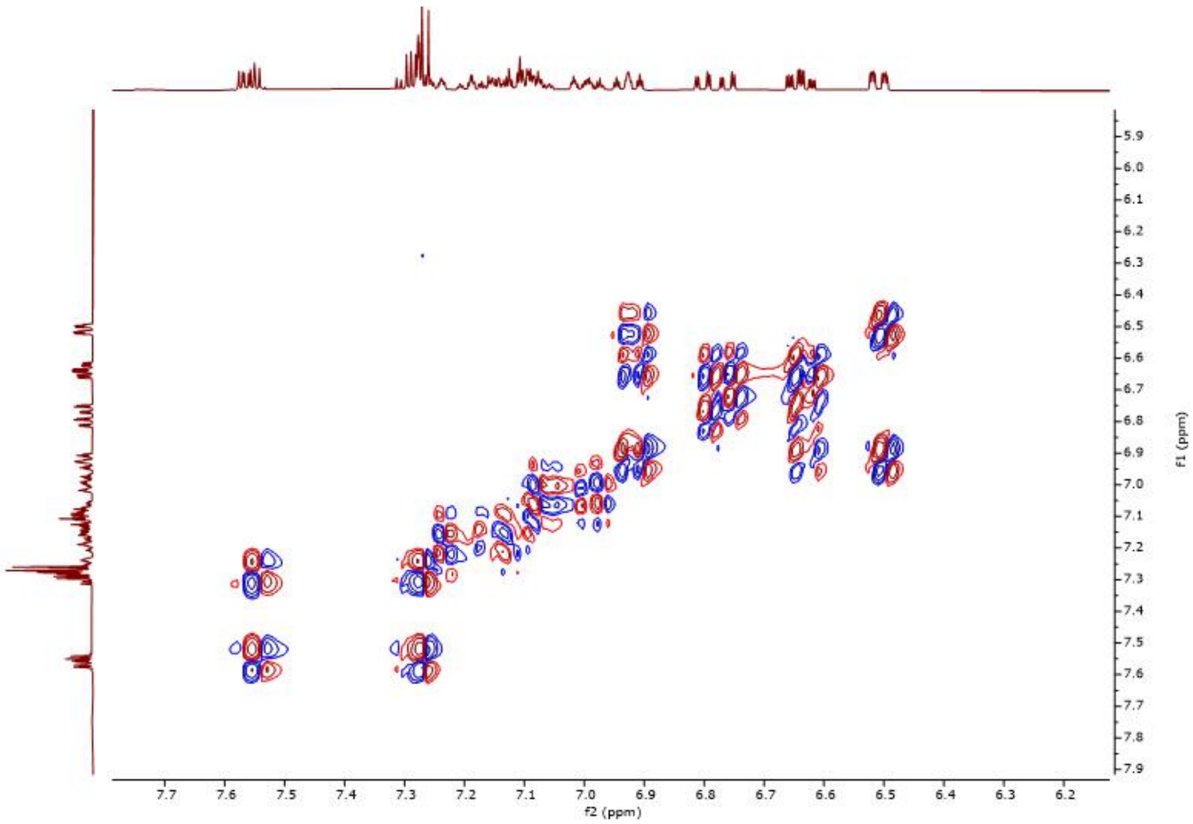

Figure S51. COSY NMR (400 MHz) of **6** in $CDCl_3$, measured at 298 K (expansion in aromatic region).

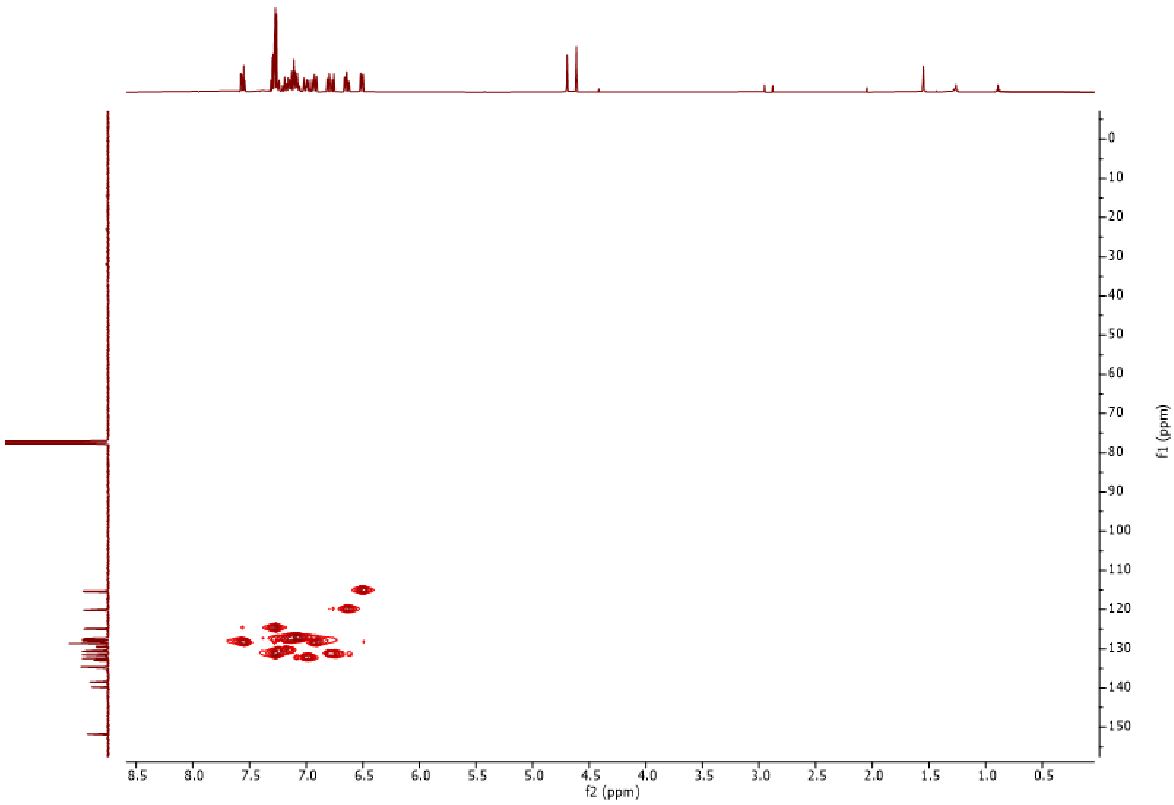

Figure S52. HSQC NMR (400 MHz) of **6** in $CDCl_3$, measured at 298 K.

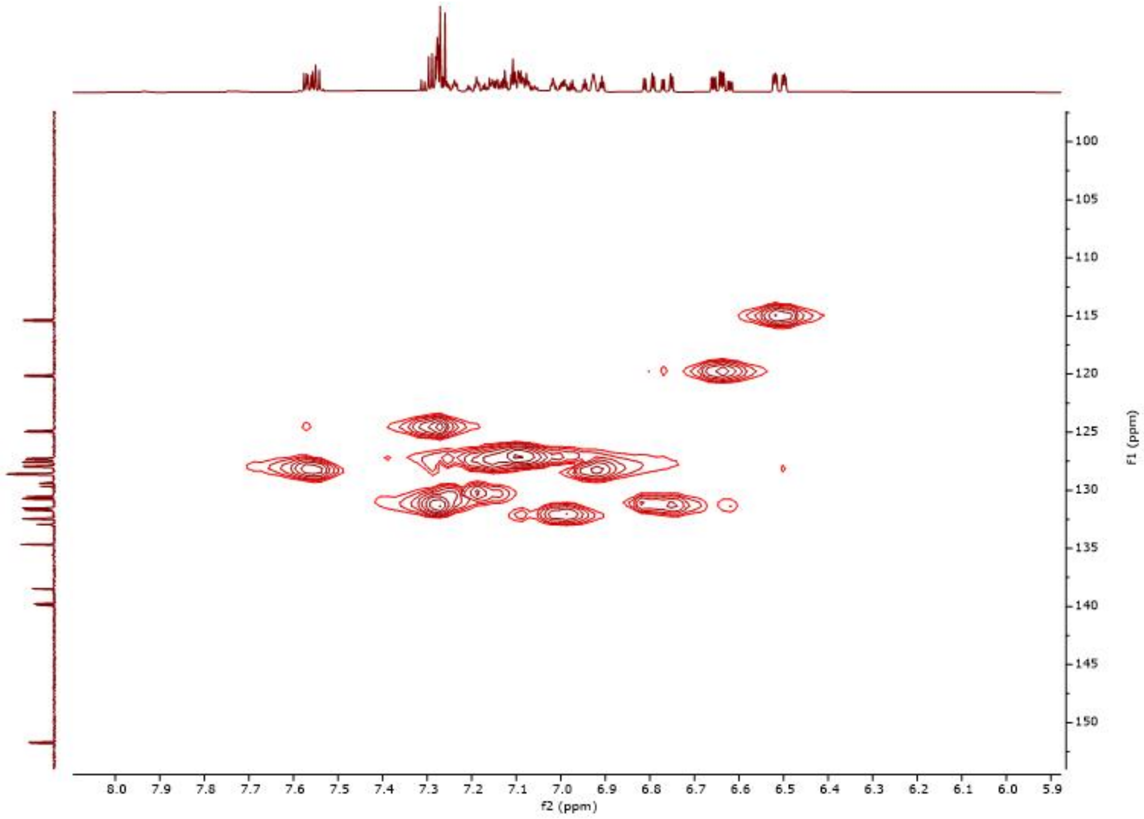

Figure S53. HSQC NMR (400 MHz) of **6** in $CDCl_3$, measured at 298 K (expansion in aromatic region).

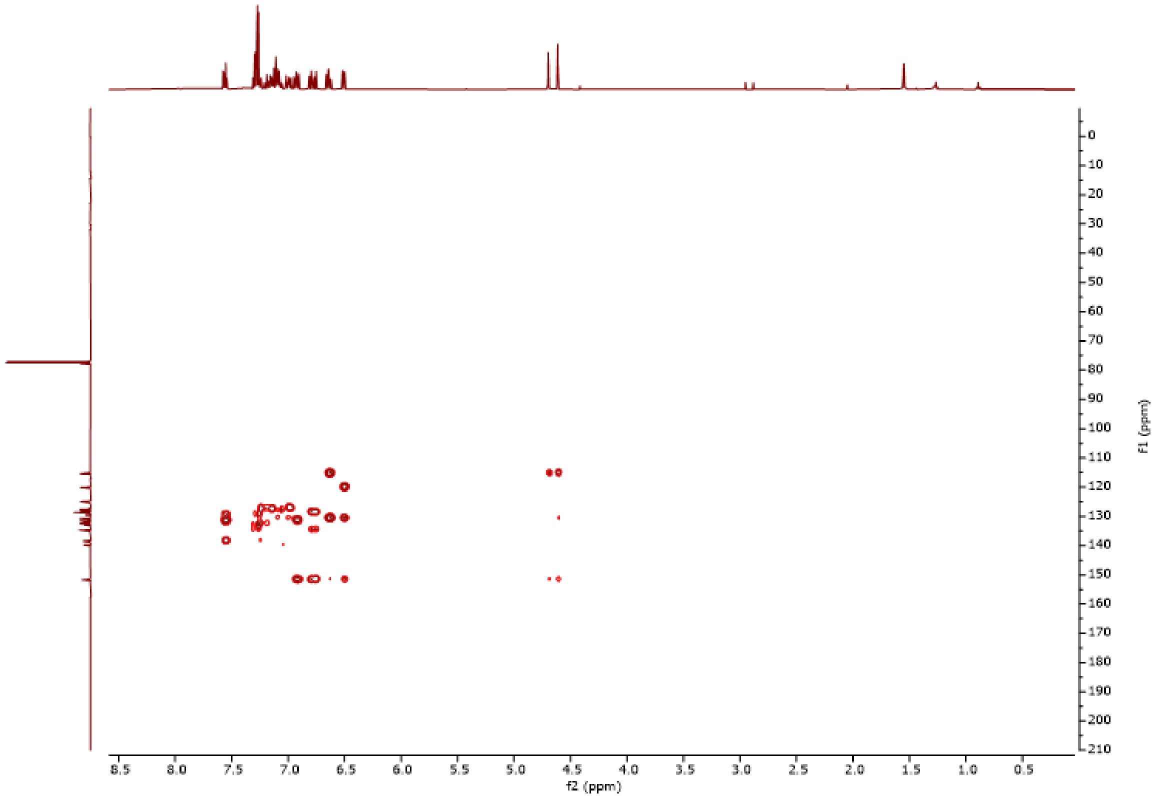

Figure S54. HMBC NMR (400 MHz) of **6** in $CDCl_3$, measured at 298 K.

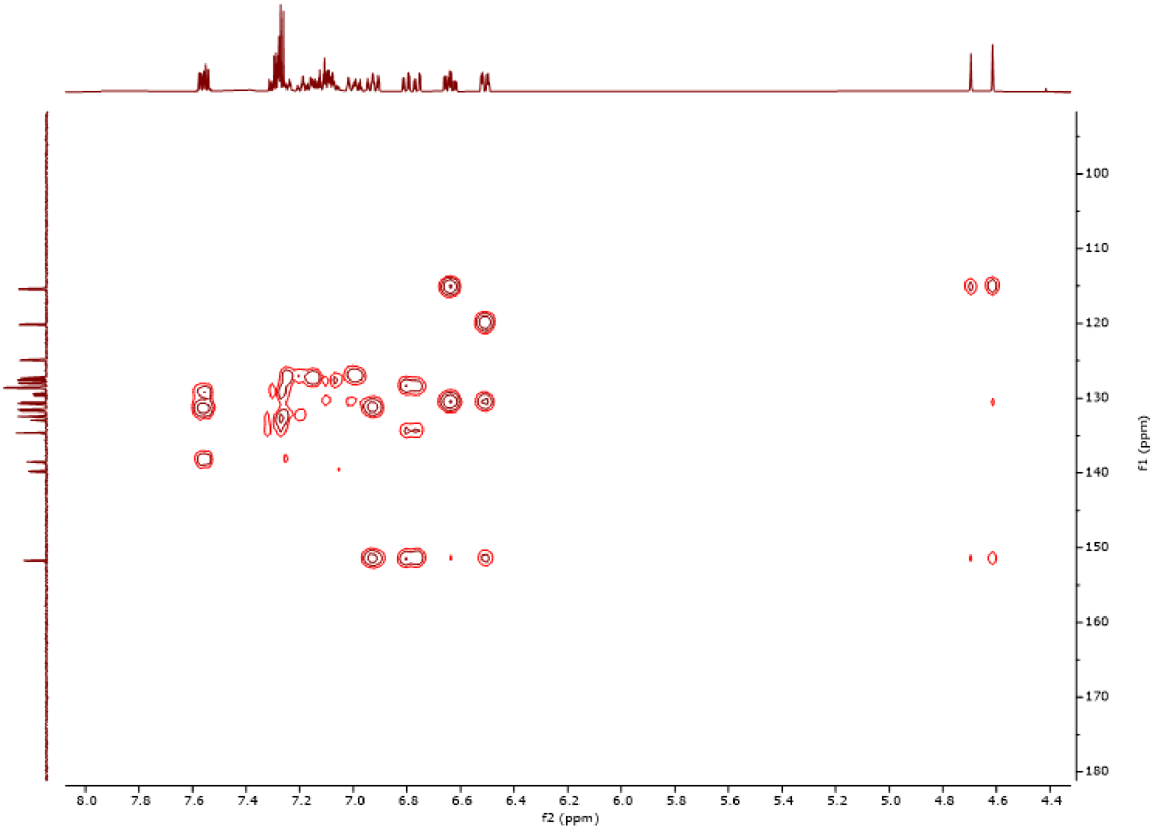

Figure S55. HMBC NMR (400 MHz) of **6** in $CDCl_3$, measured at 298 K (expansion in aromatic region).

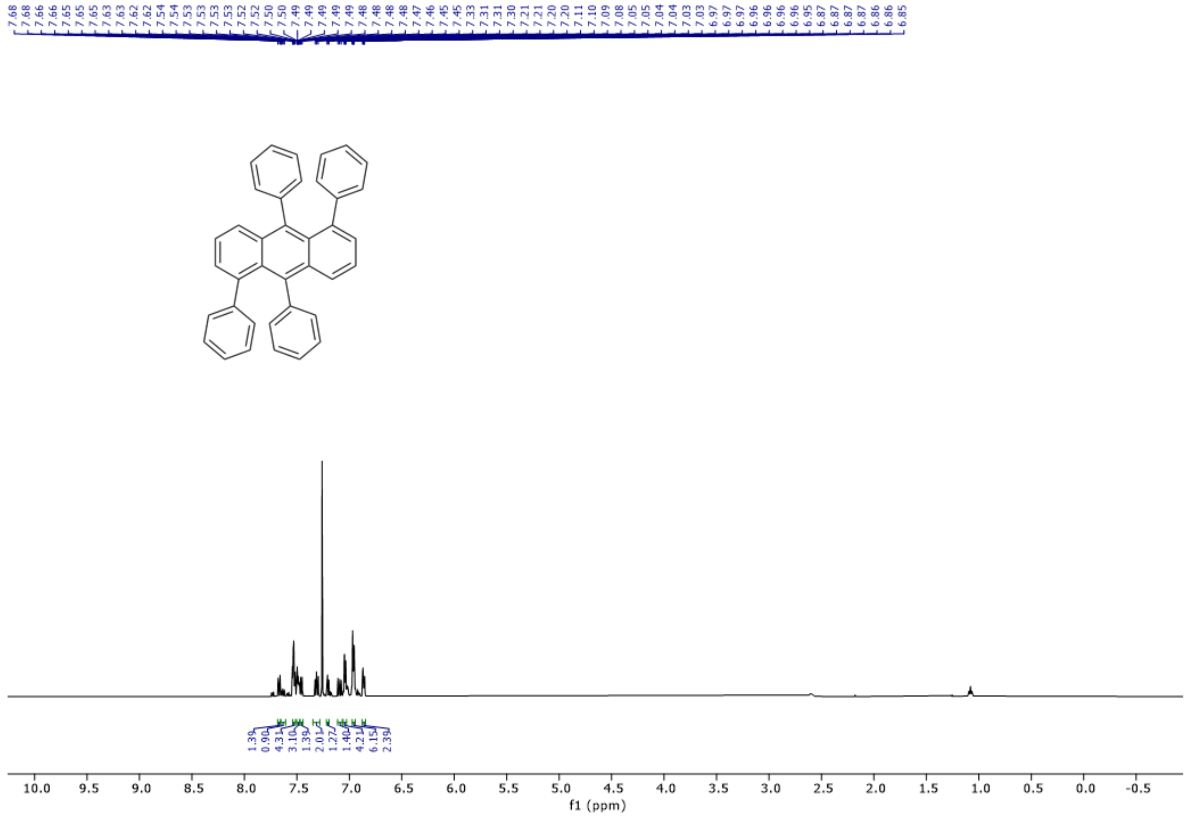

Figure S56. ^{1}H NMR (500 MHz) of **1-C0** in $CDCl_3$, measured at 298 K.

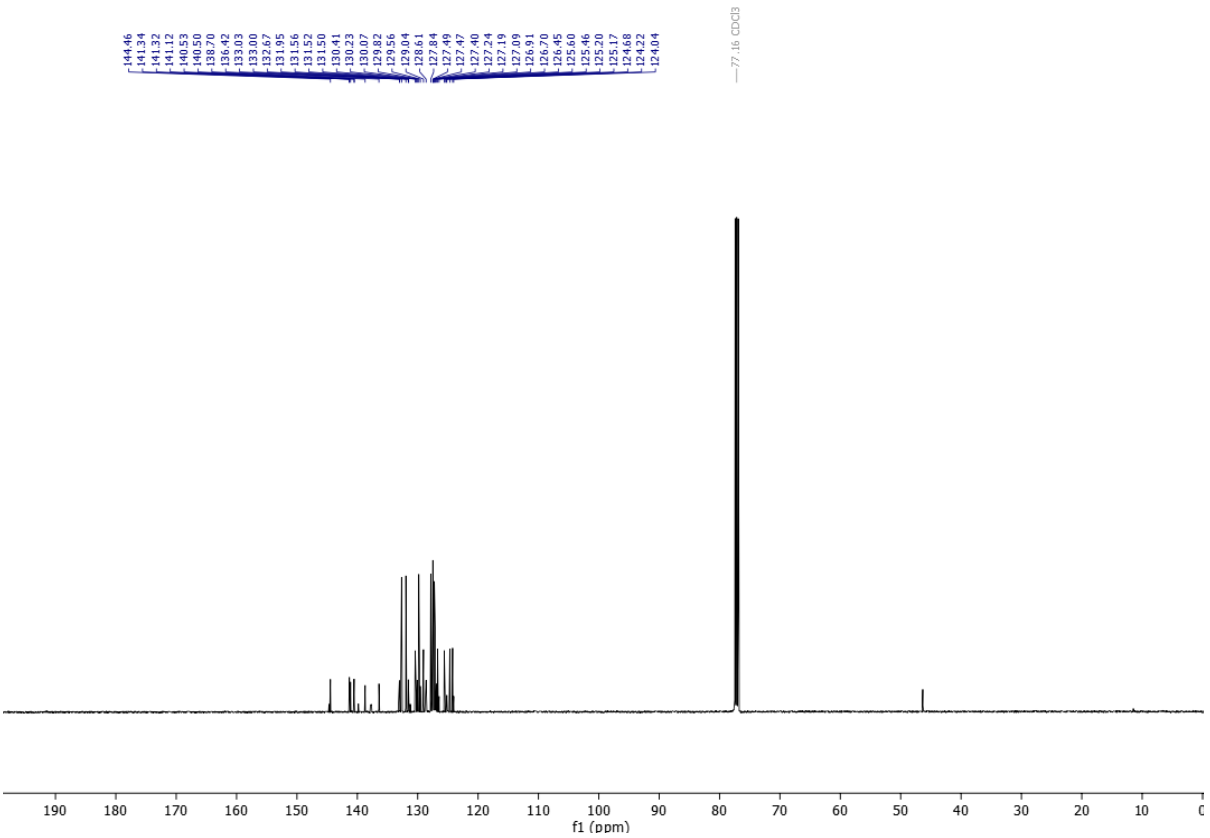

Figure S57. ^{13}C NMR (125 MHz) of **1-C0** in $CDCl_3$, measured at 298 K.

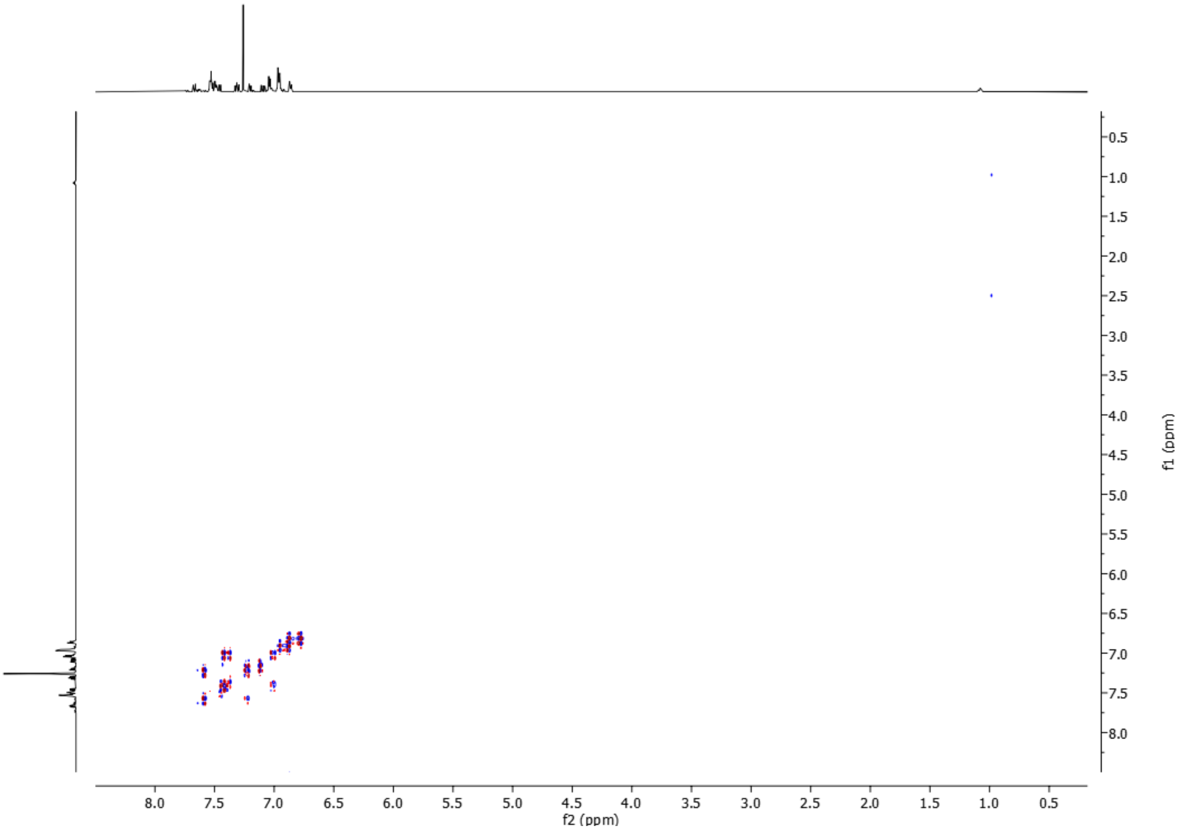

Figure S58. COSY NMR (500 MHz) of **1-C0** in $CDCl_3$, measured at 298 K.

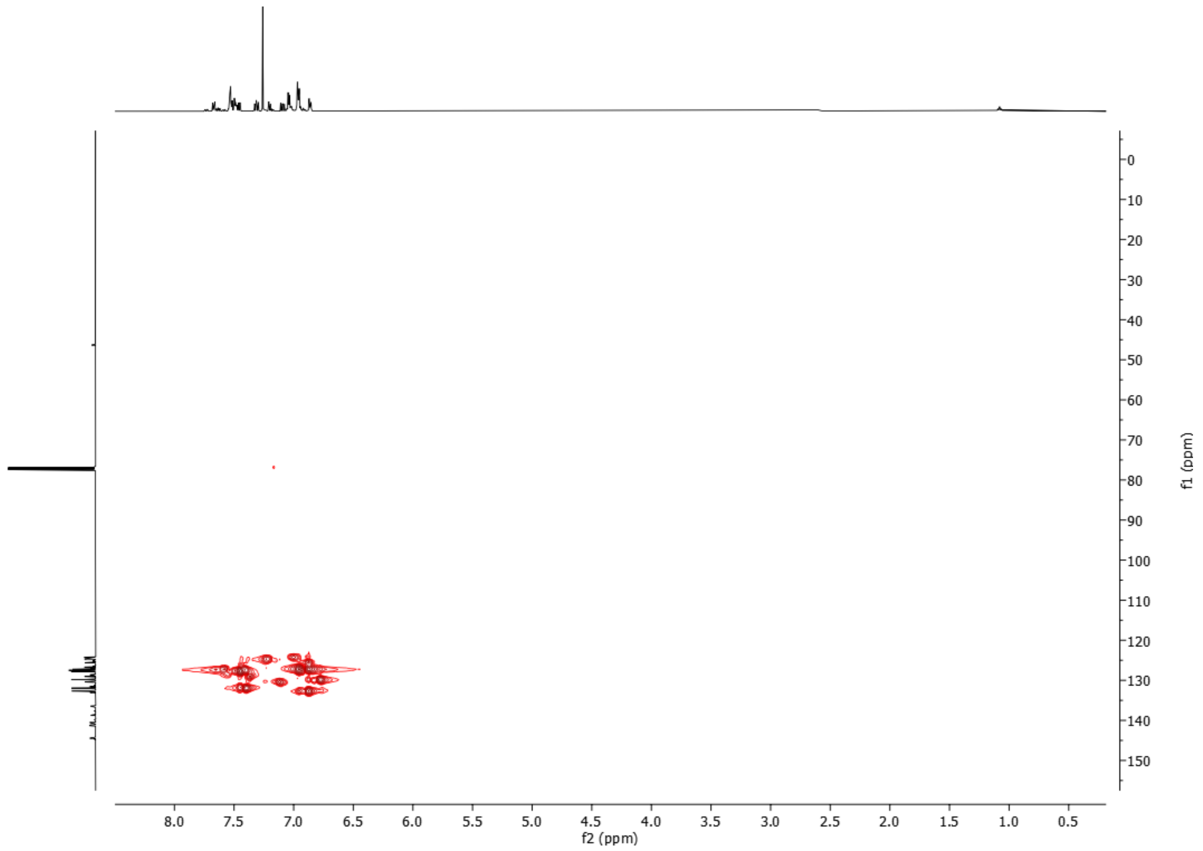

Figure S59. HSQC NMR (500 MHz) of **1-C0** in $CDCl_3$, measured at 298 K.

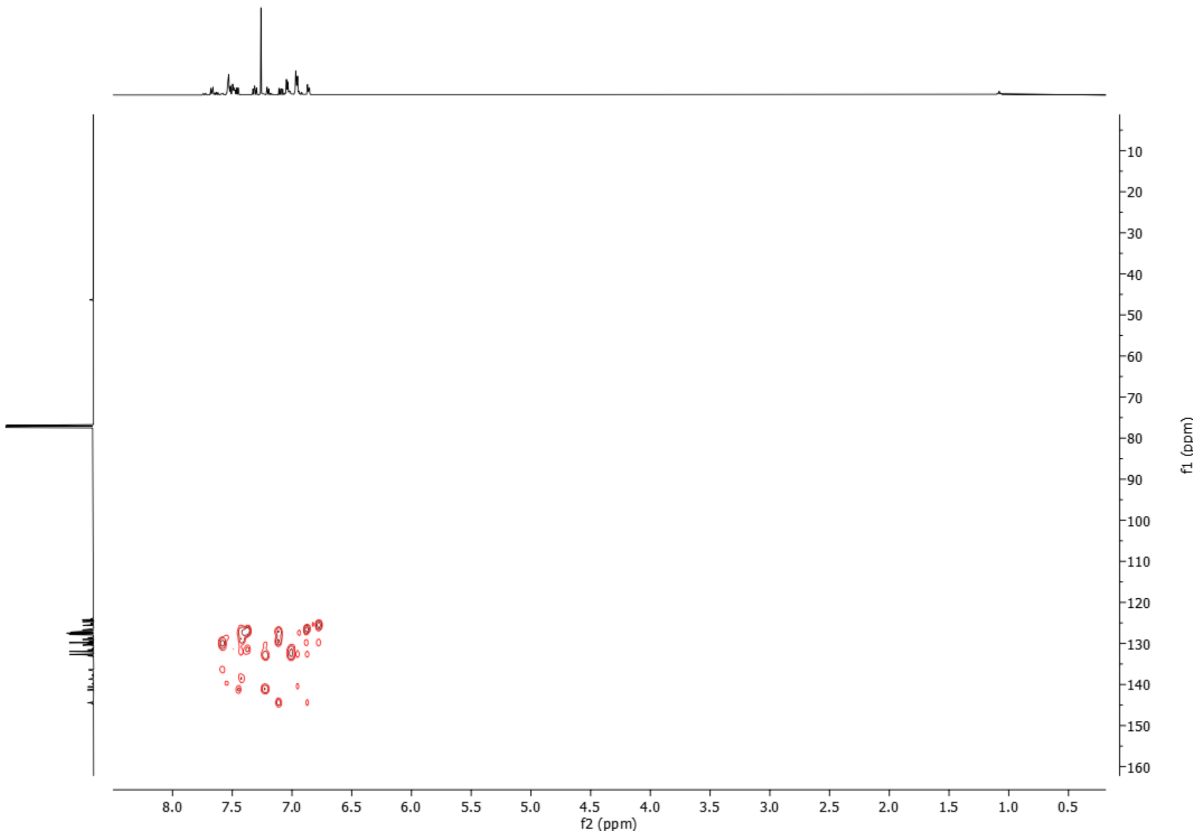

Figure S60. HMBC NMR (500 MHz) of **1-C0** in $CDCl_3$, measured at 298 K.

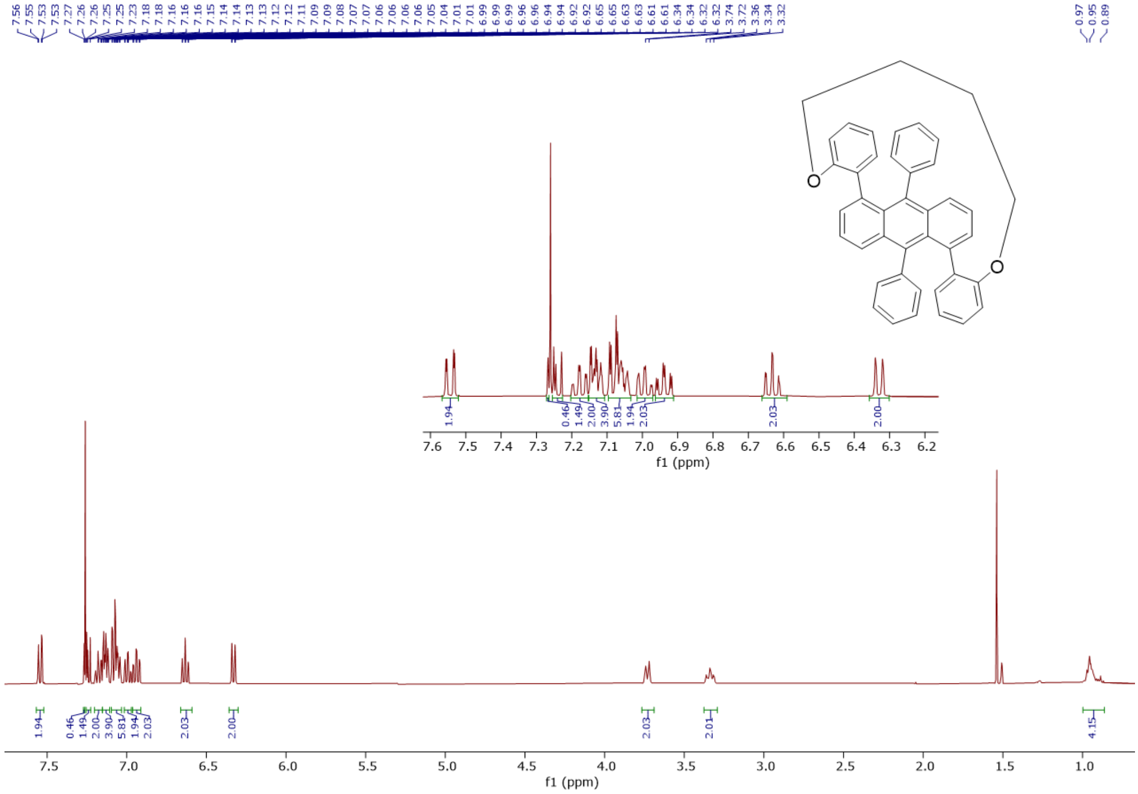

Figure S61. ^{1}H NMR (400 MHz) of **1-C4** in $CDCl_3$, measured at 298 K.

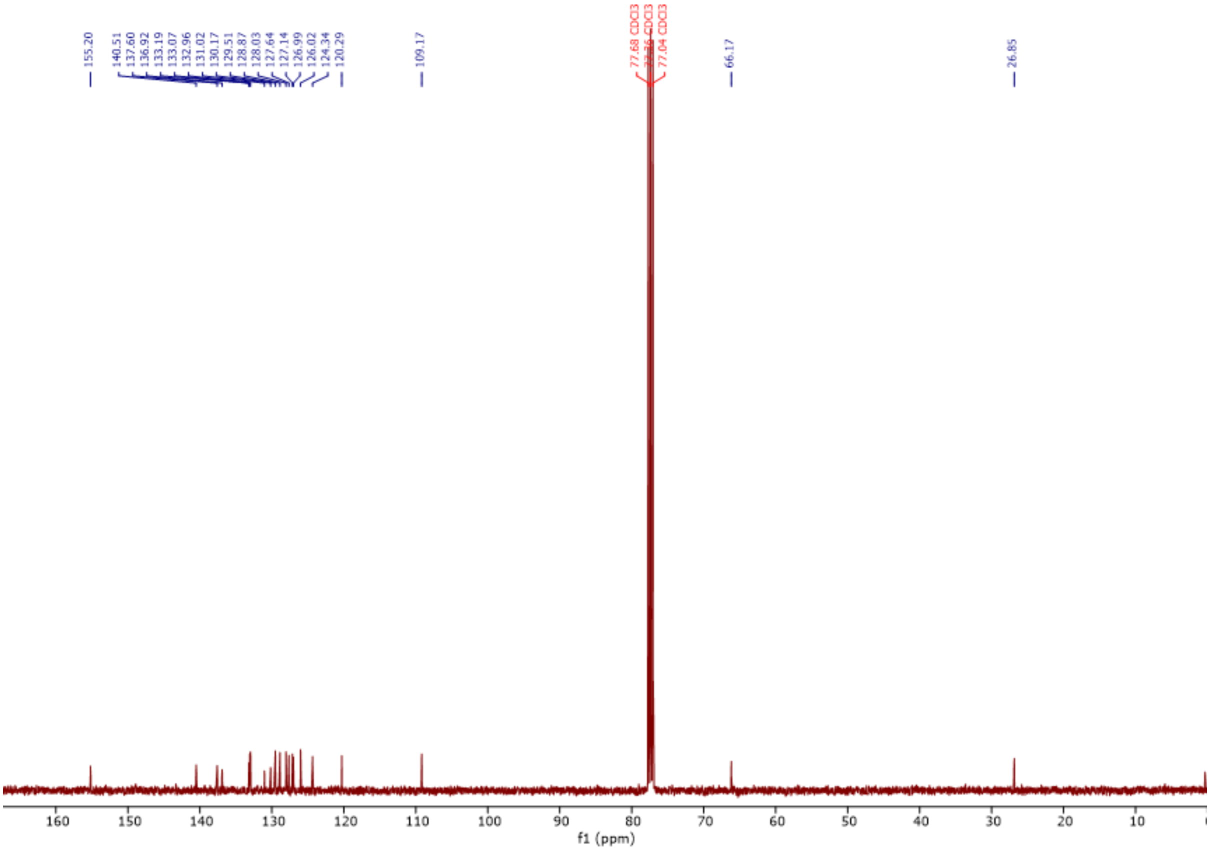

Figure S62. ^{13}C NMR (400 MHz) of **1-C4** in $CDCl_3$, measured at 298 K.

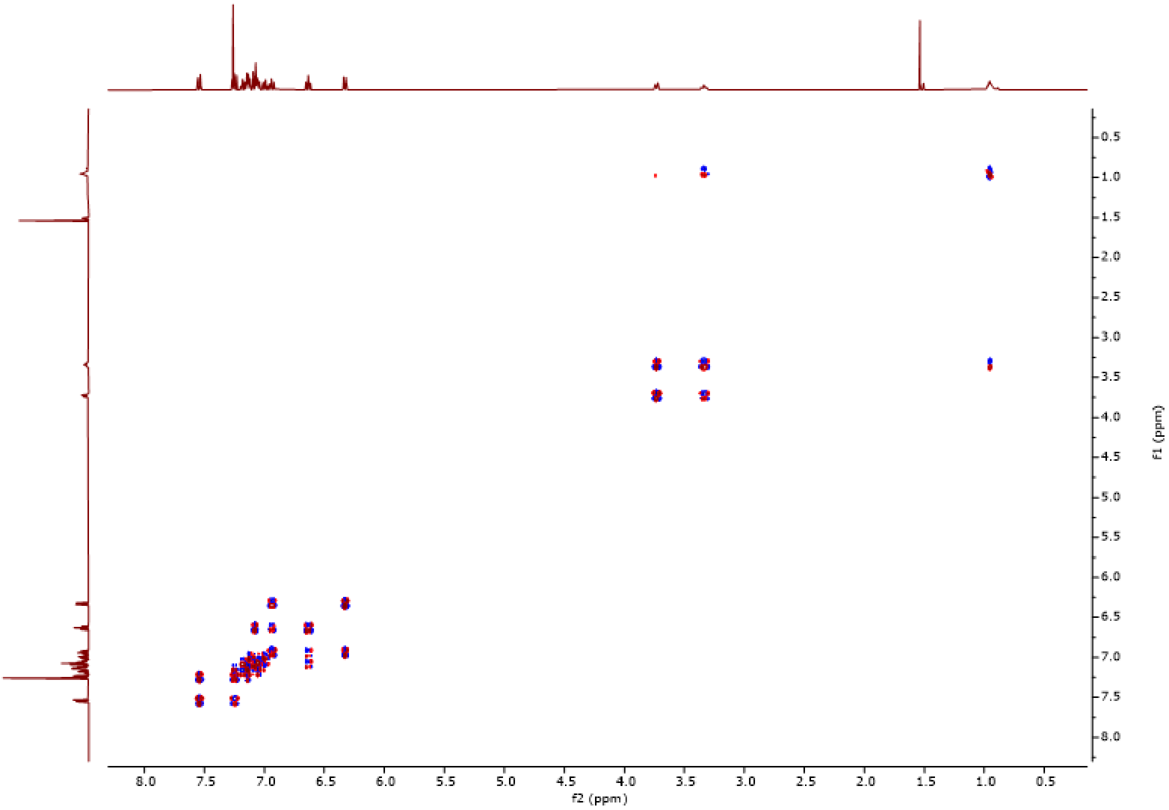

Figure S63. COSY NMR (400 MHz) of **1-C4** in $CDCl_3$, measured at 298 K.

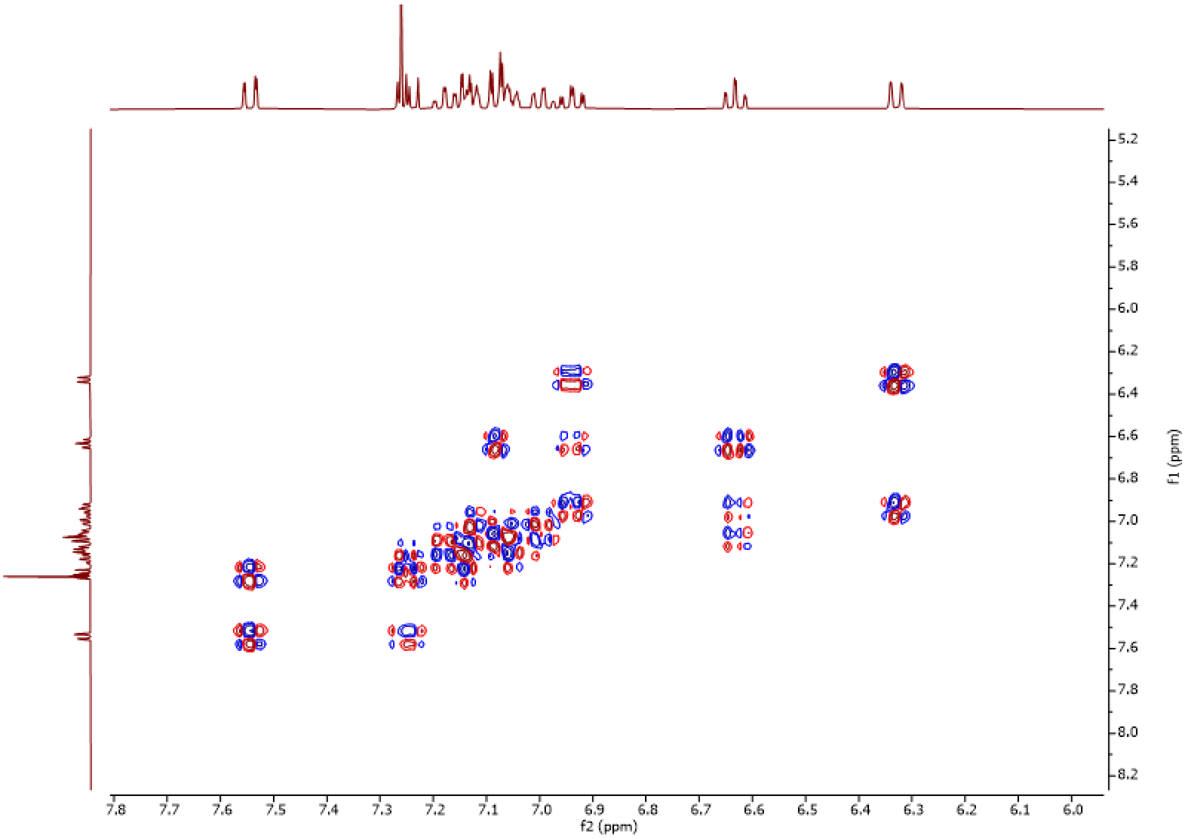

Figure S64. COSY NMR (400 MHz) of **1-C4** in $CDCl_3$, measured at 298 K (expansion in aromatic region).

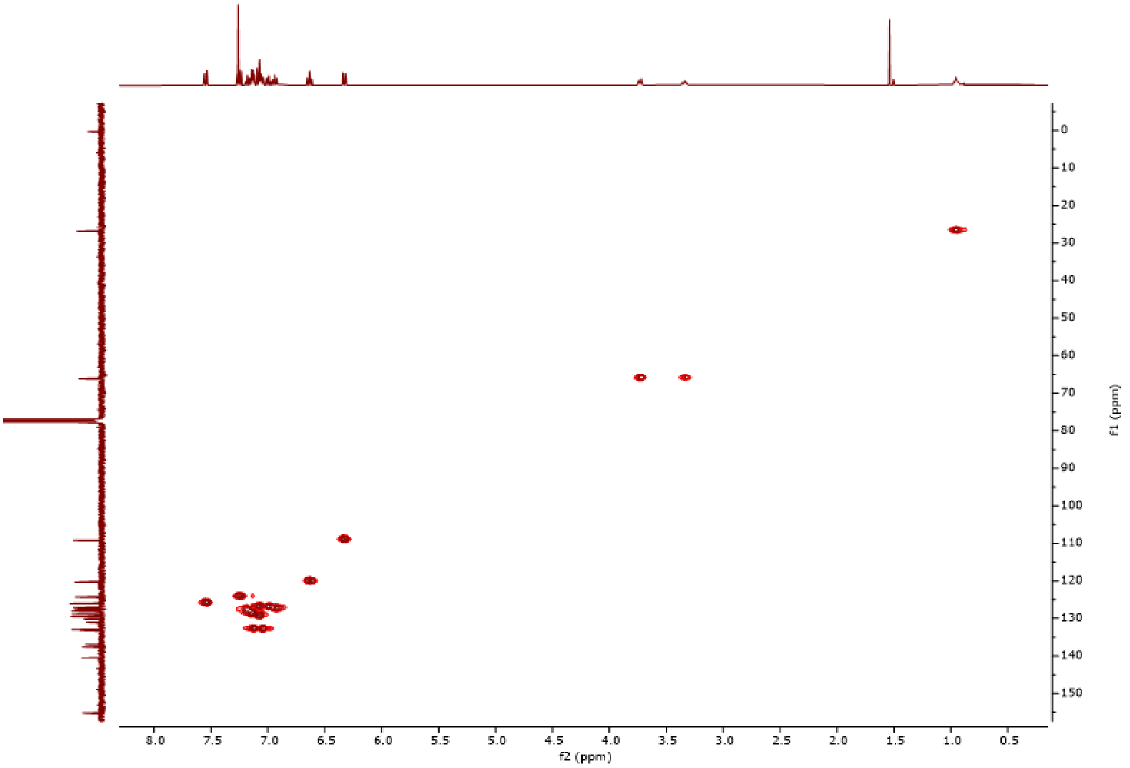

Figure S65. HSQC NMR (400 MHz) of **1-C4** in $CDCl_3$, measured at 298 K.

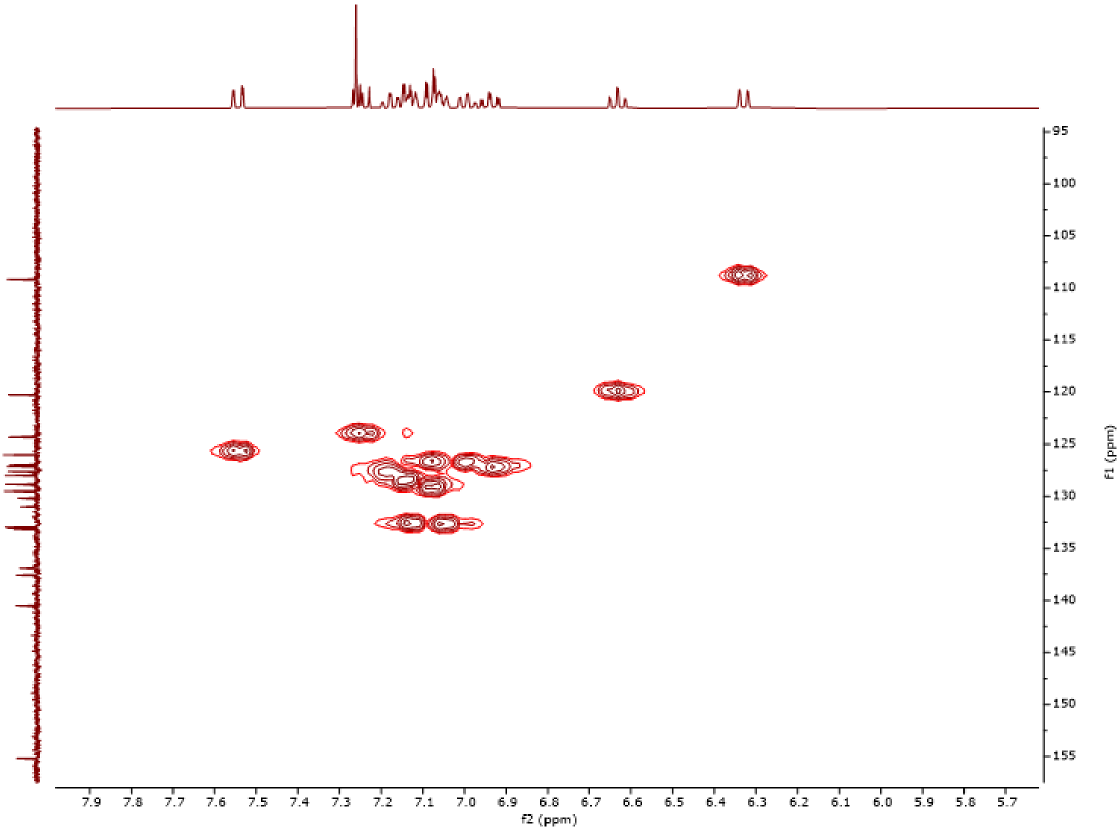

Figure S66. HSQC NMR (400 MHz) of **1-C4** in $CDCl_3$, measured at 298 K (expansion in aromatic region).

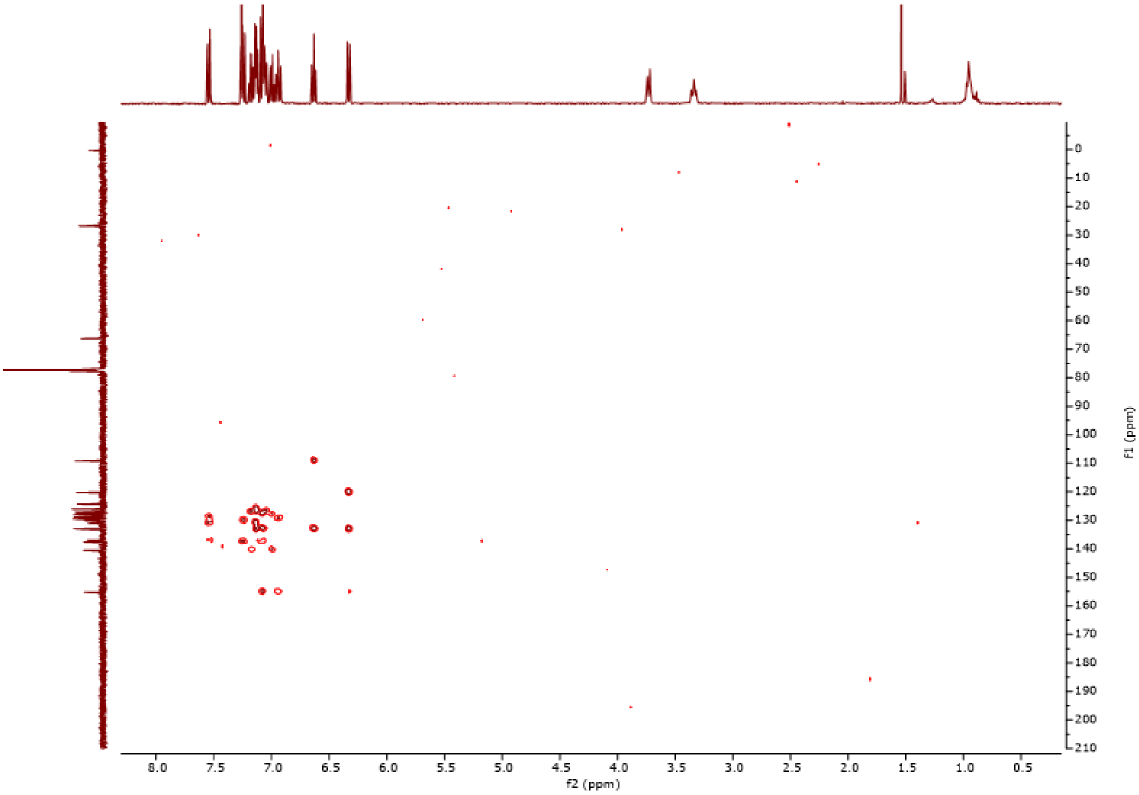

Figure S67. HMBC NMR (400 MHz) of **1-C4** in $CDCl_3$, measured at 298 K.

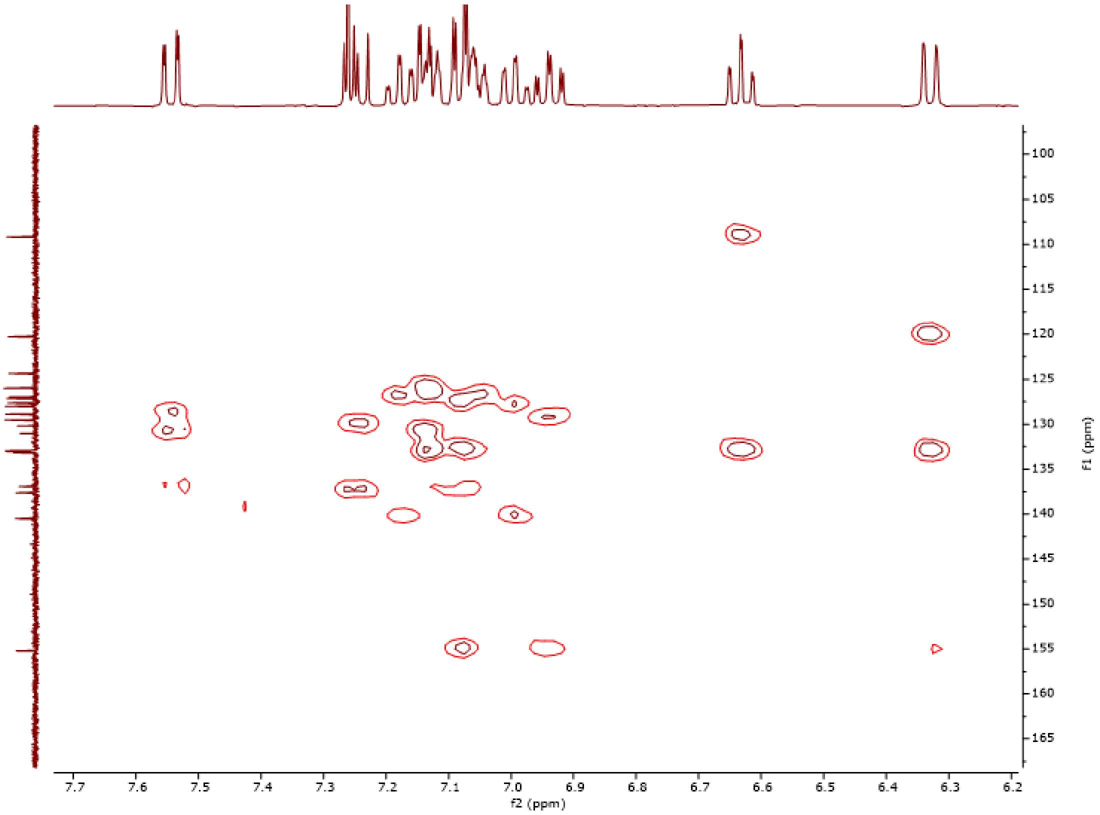

Figure S68. HMBC NMR (400 MHz) of **1-C4** in $CDCl_3$, measured at 298 K (expansion in aromatic region).

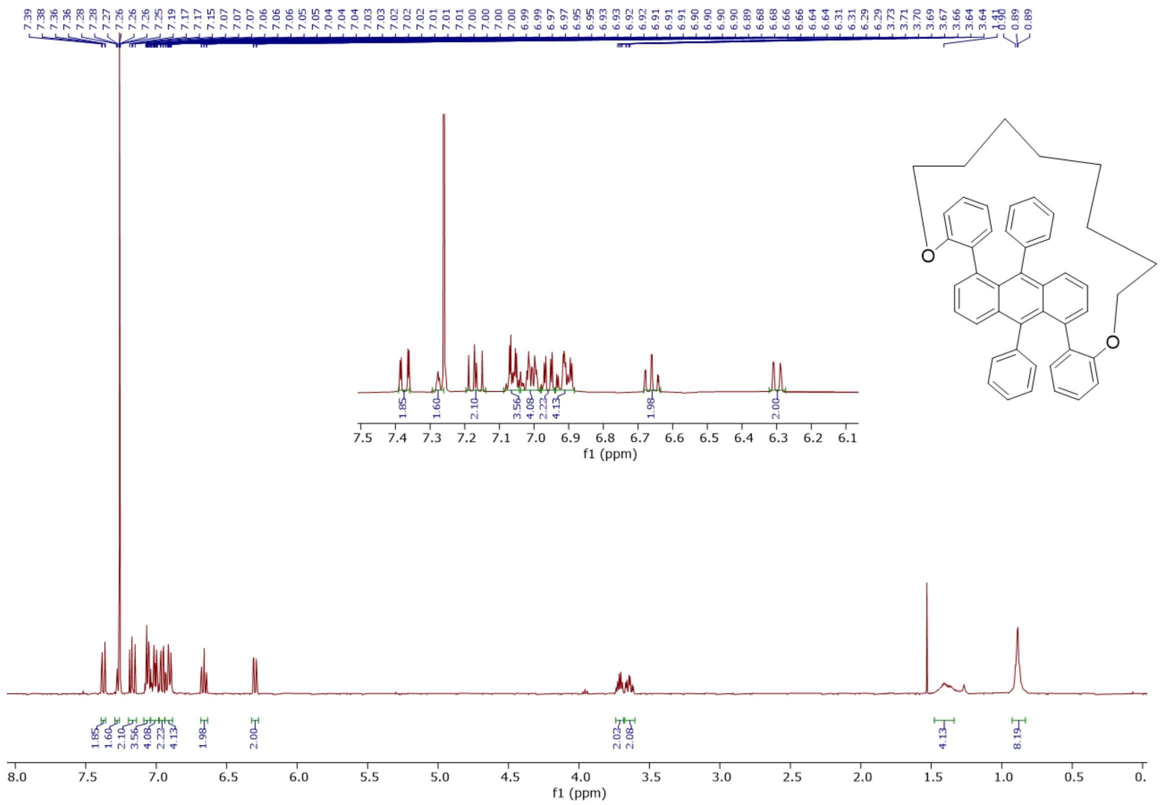

Figure S69. ^{1}H NMR (400 MHz) of **1-C8** in $CDCl_3$, measured at 298 K.

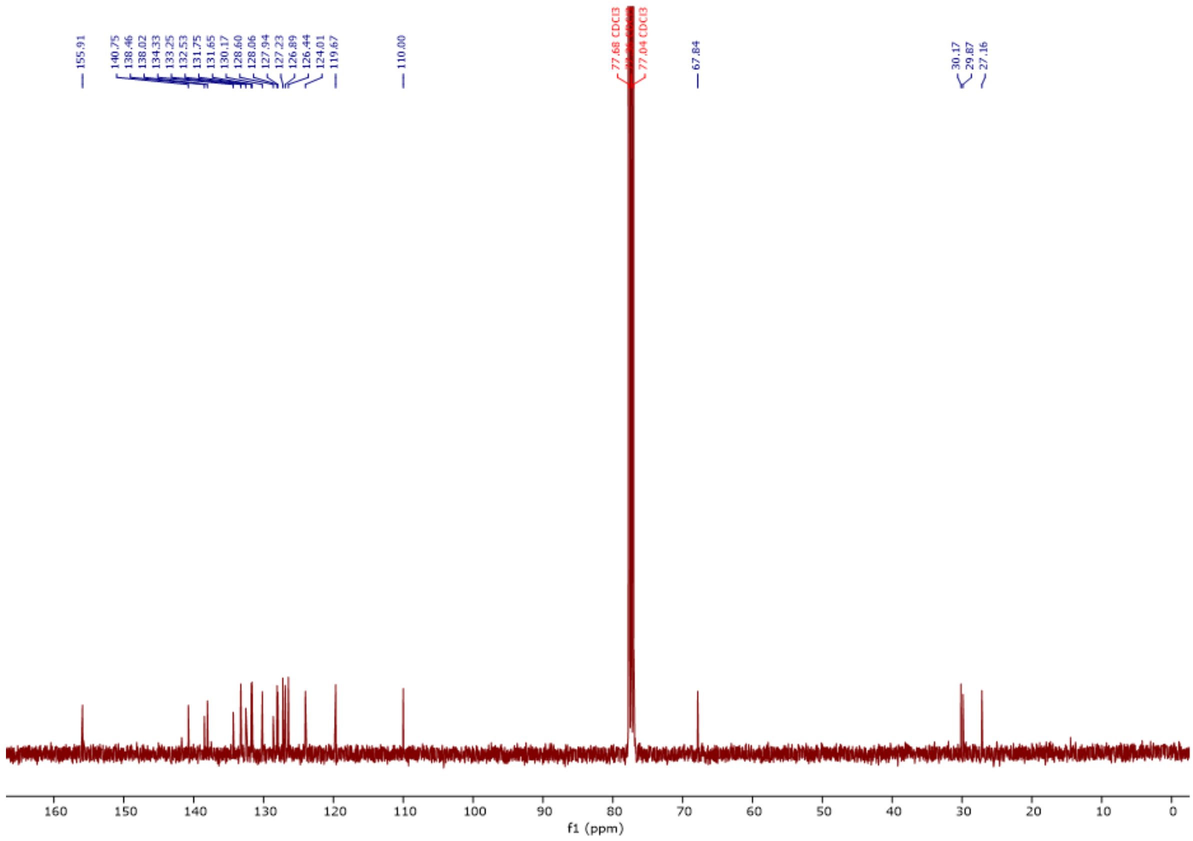

Figure S70. ^{13}C NMR (400 MHz) of **1-C8** in $CDCl_3$, measured at 298 K.

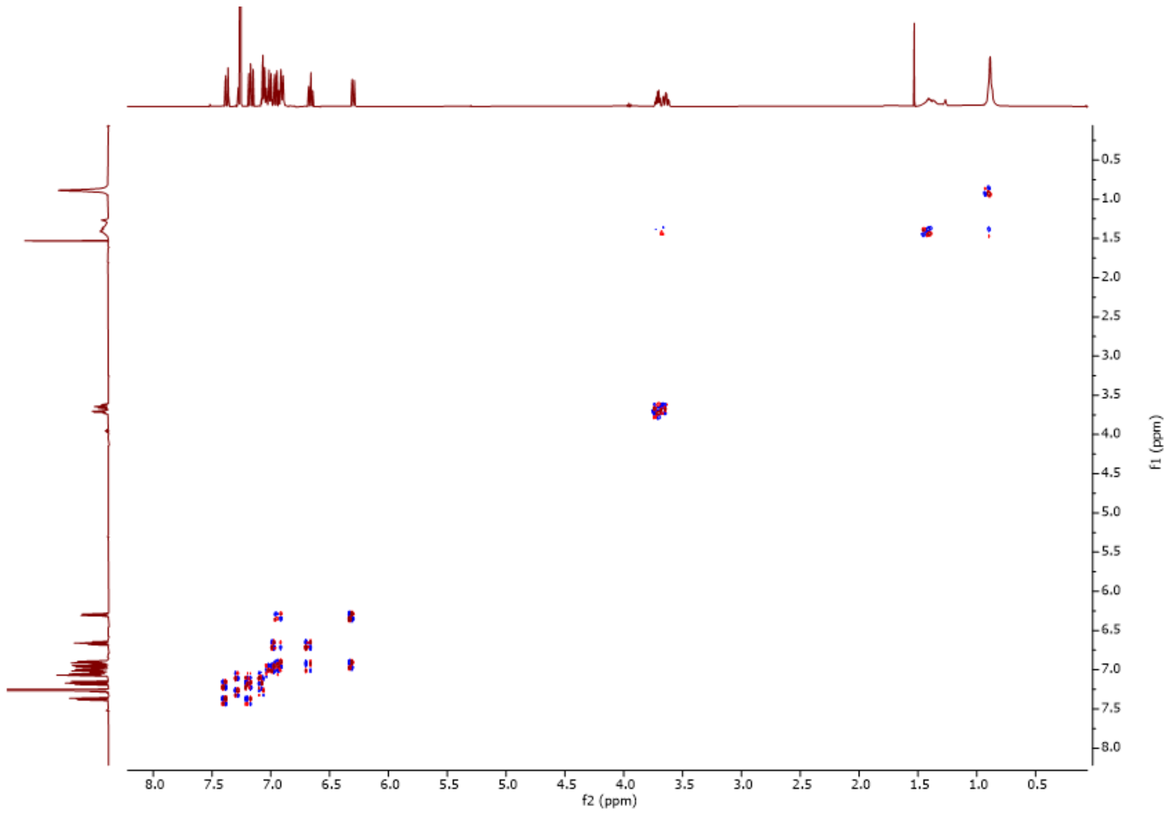

Figure S71. COSY NMR (400 MHz) of **1-C8** in $CDCl_3$, measured at 298 K.

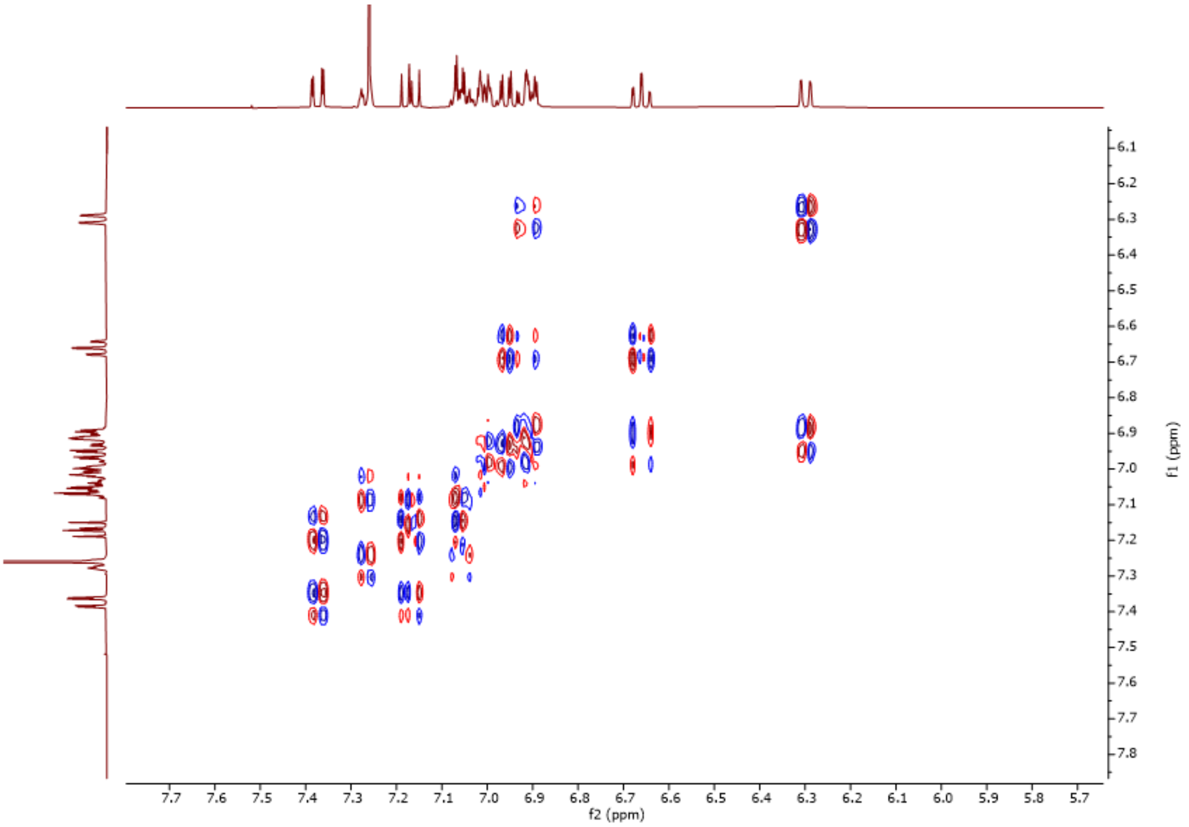

Figure S72. COSY NMR (400 MHz) of **1-C8** in $CDCl_3$, measured at 298 K (expansion in aromatic region).

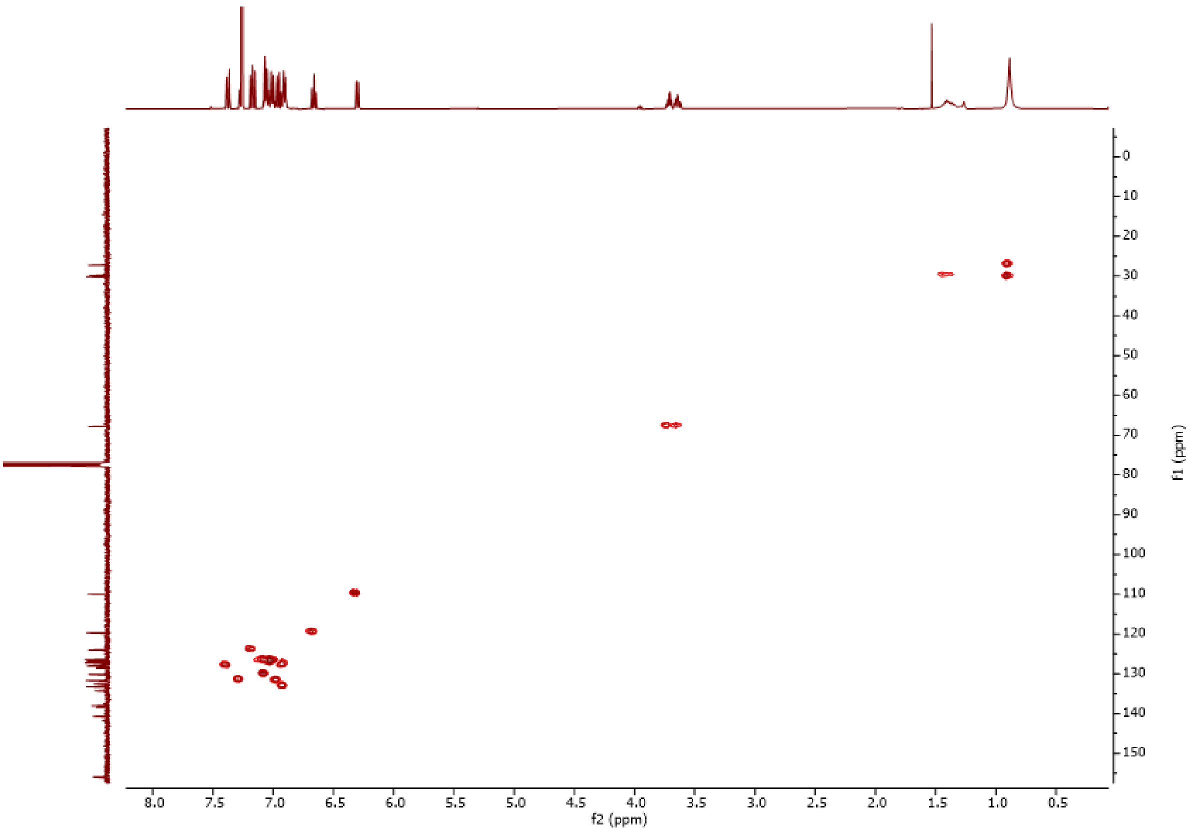

Figure S73. HSQC NMR (400 MHz) of **1-C8** in $CDCl_3$, measured at 298 K.

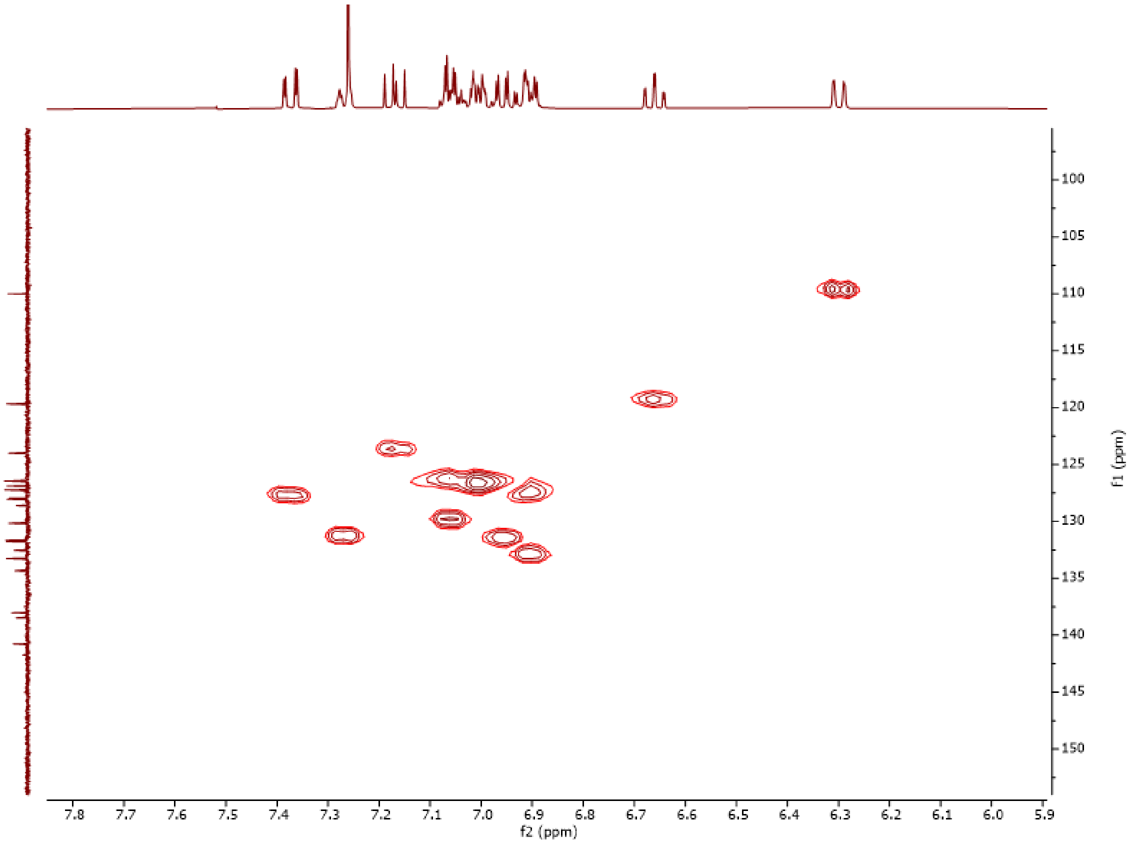

Figure S74. HSQC NMR (400 MHz) of **1-C8** in $CDCl_3$, measured at 298 K (expansion in aromatic region).

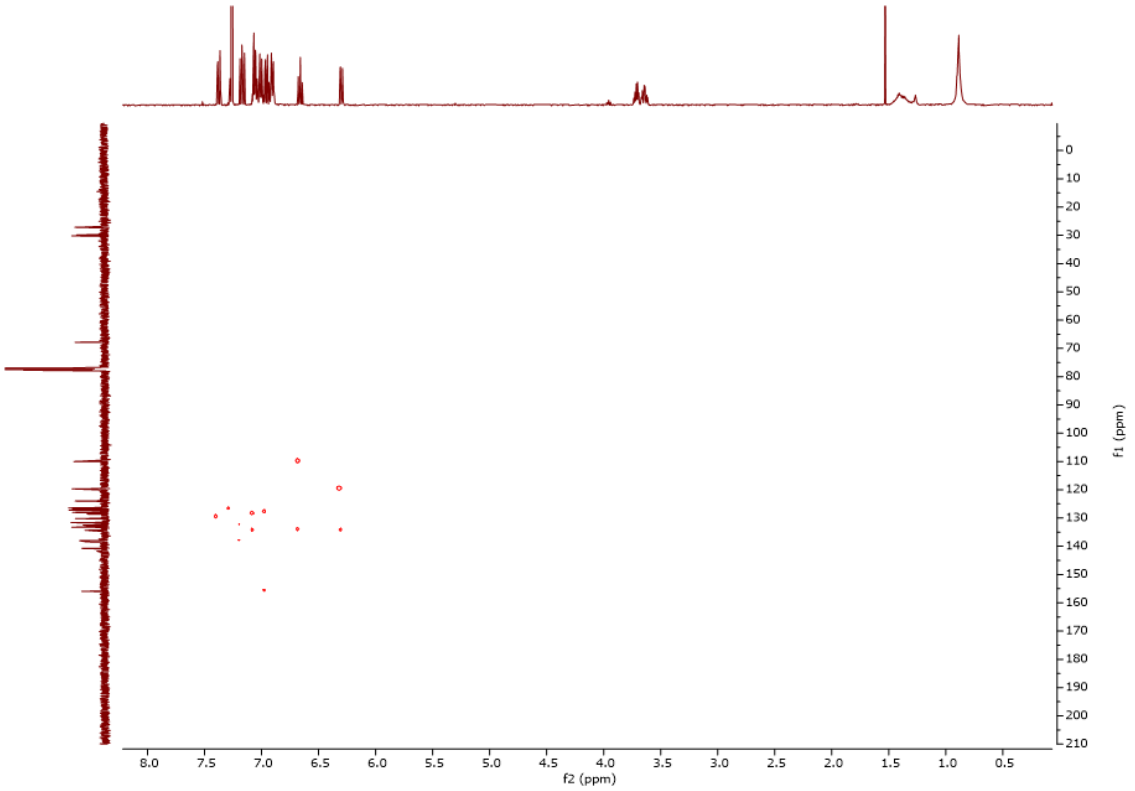

Figure S75. HMBC NMR (400 MHz) of **1-C8** in $CDCl_3$, measured at 298 K.

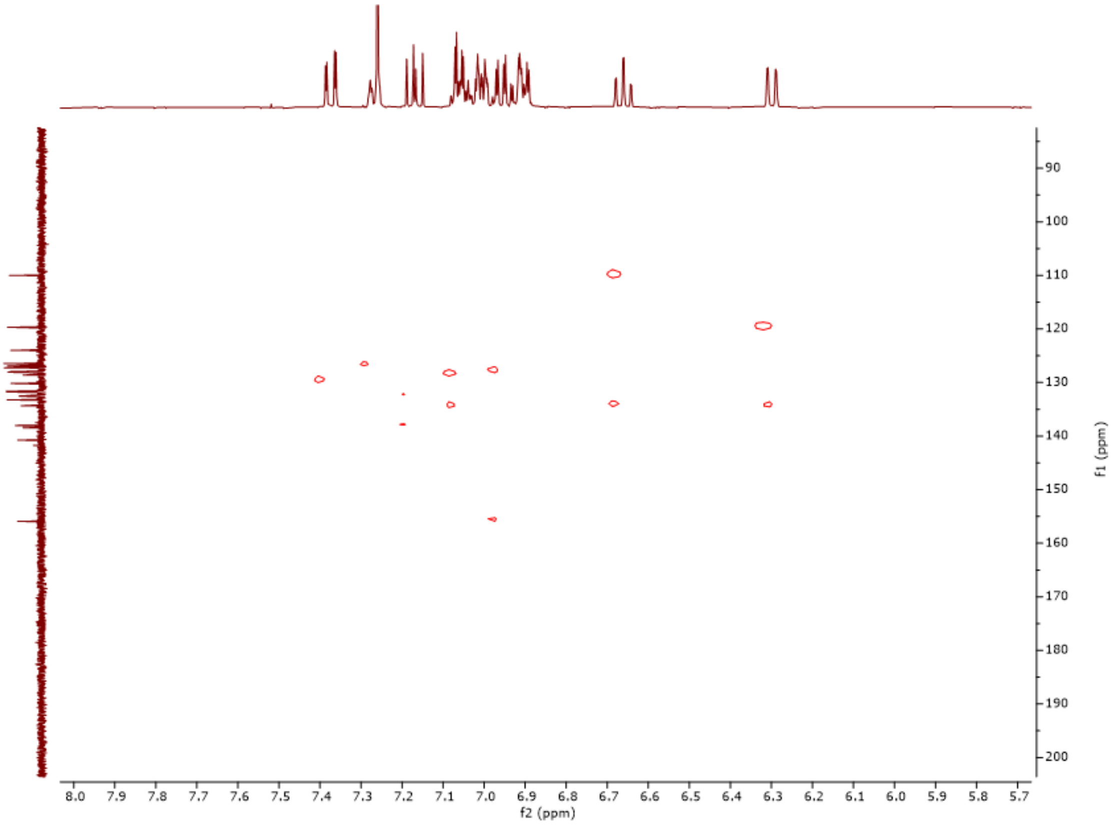

Figure S76. HMBC NMR (400 MHz) of **1-C8** in $CDCl_3$, measured at 298 K (expansion in aromatic region).

S4 HPLC chiral separation

Racemic **1-Cn** and **2-Cn** (n= 4, 8) were resolved using semi-preparative CHIRALPAK-IG column using 20% dichloromethane/hexane as the eluent, the *P*-enantiomer was eluted first followed by the *M*-enantiomer.

S4.1 **1-Cn**

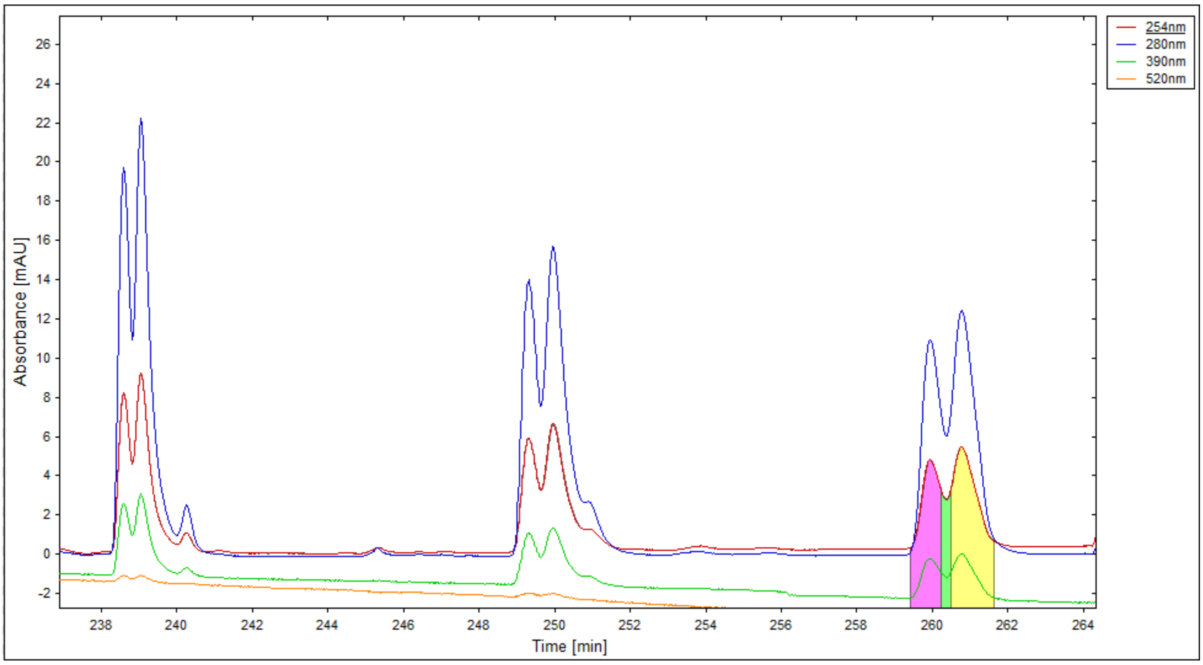

Figure S77. Chiral HPLC separation of **1-C4.**

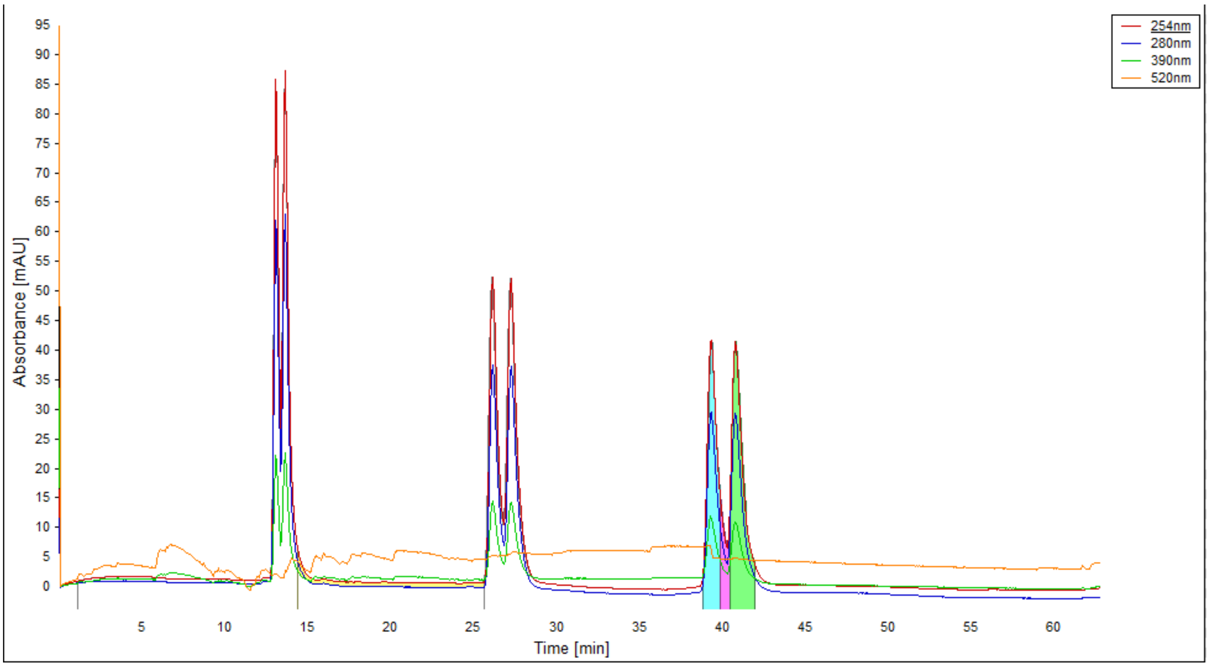

Figure S78. Chiral HPLC separation of **1-C8.**

S4.2 **2-Cn**

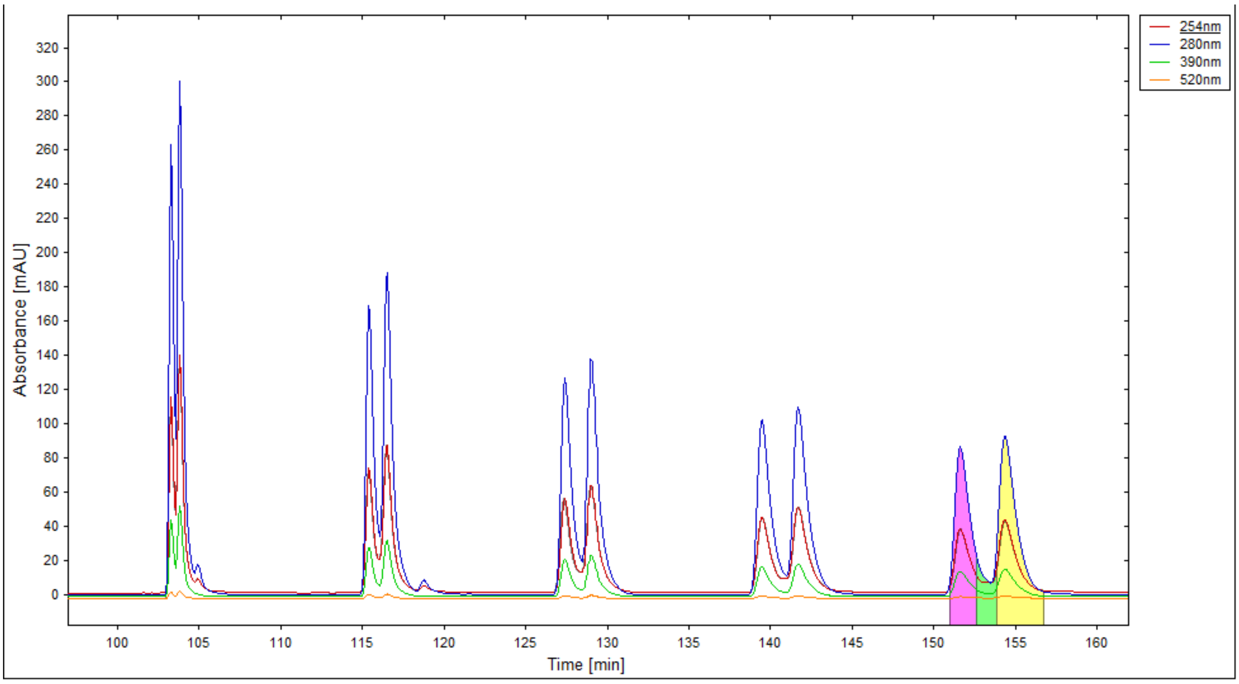

Figure S79. Chiral HPLC separation of **2-C4.**

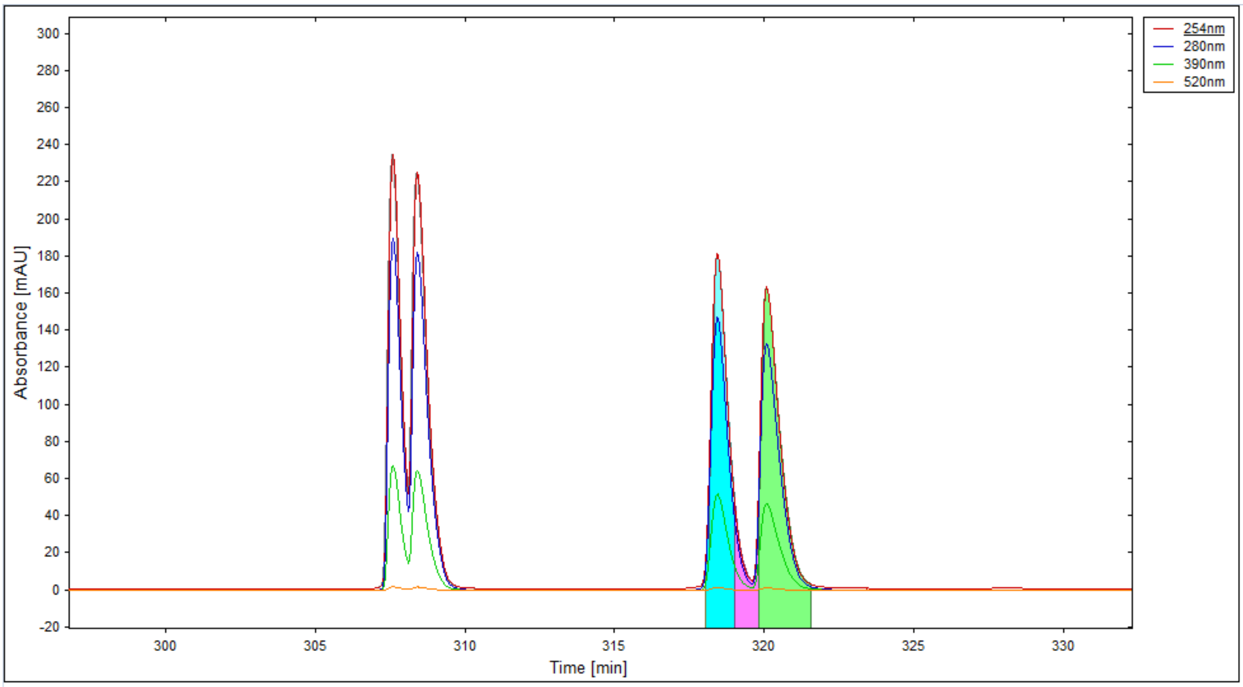

Figure S80. Chiral HPLC separation of **2-C8.**

S5 Photophysical Properties

S5.1 UV-vis absorption spectra

S5.1.1 **1-Cn**

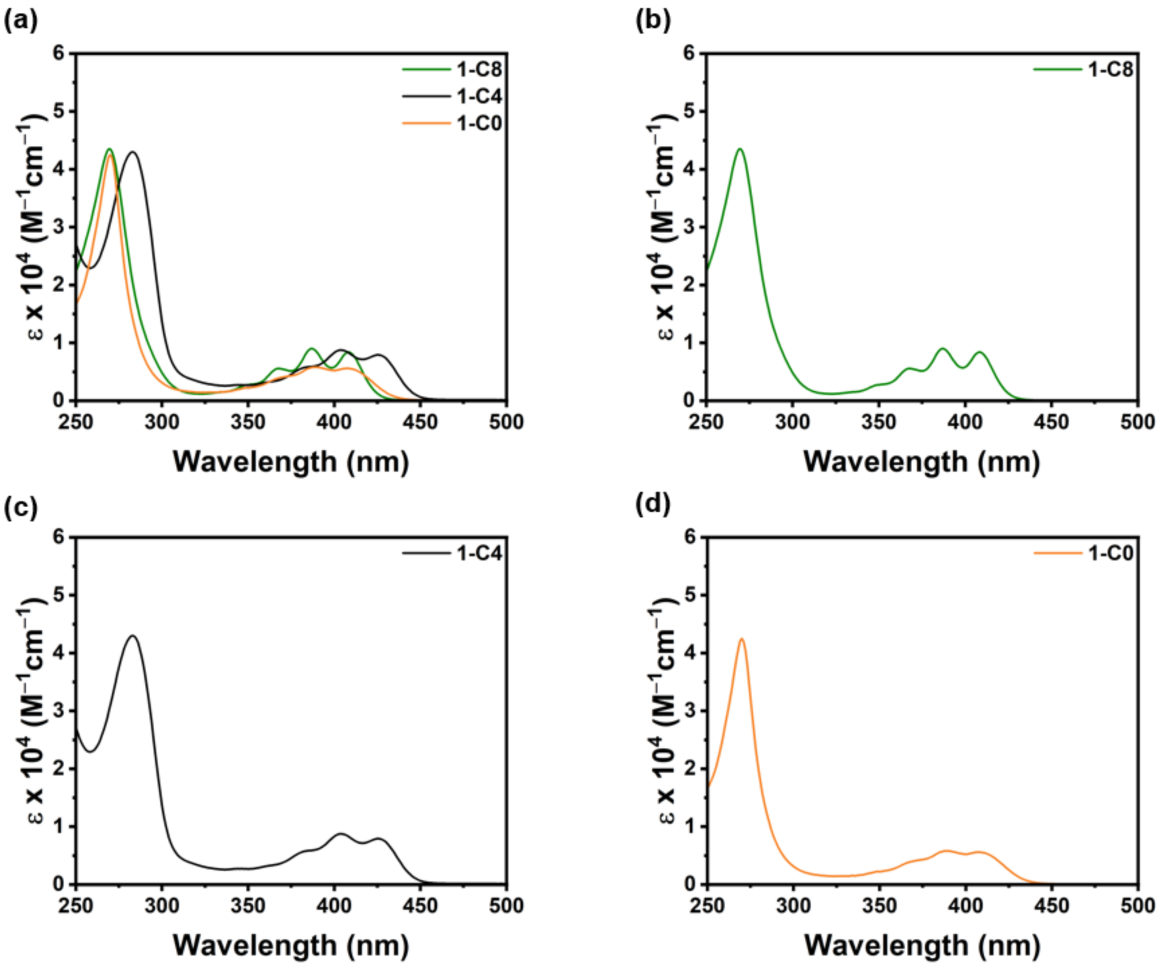

Figure S81. (a) Absorption spectra of **1-Cn** (n=0, 4, 8) in chloroform, measured at 298 K. (b) **1-C8** (c) **1-C4** (d) **1-C0**.

S5.1.2 **2-Cn**

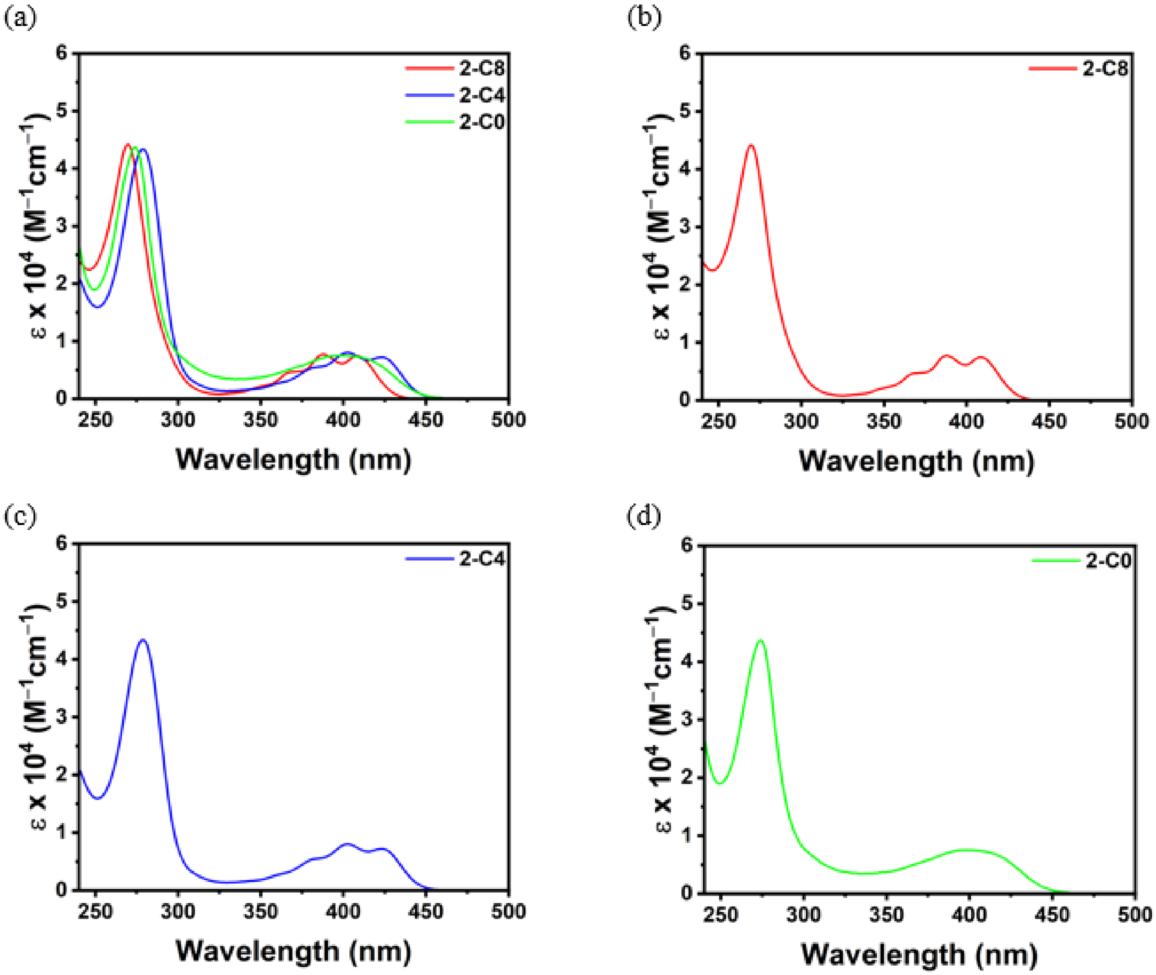

Figure S82. (a) Absorption of **2-Cn** (n=0, 4, 8) in chloroform, measured at 298 K. (b) **2-C8** (c) **2-C4** (d) **2-C0**.

S5.2 ECD spectra

S5.2.1 **1-Cn**

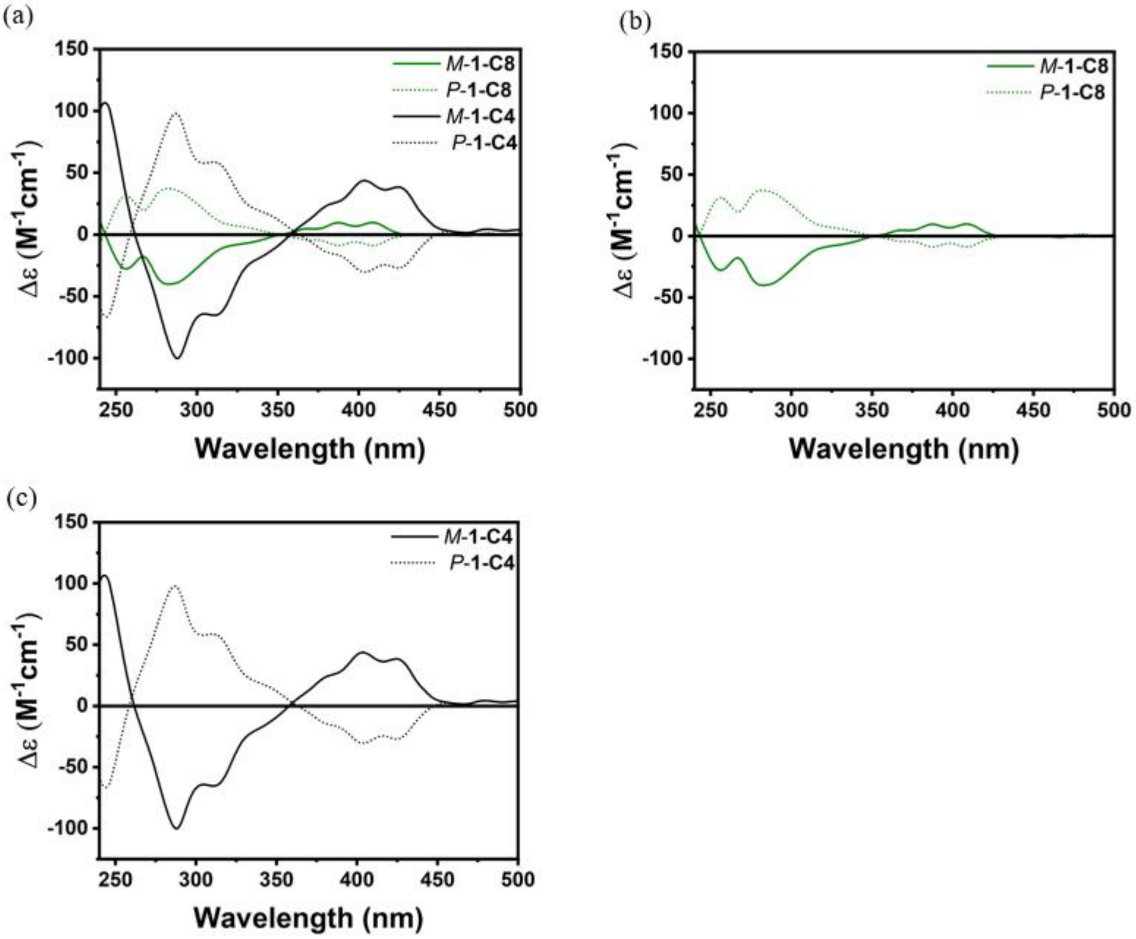

Figure S83. (a) ECD spectra of **1-Cn** (n= 4, 8) in chloroform, measured at 298 K. (b) **1-C8** (c) **1-C4**.

S5.2.2 **2-Cn**

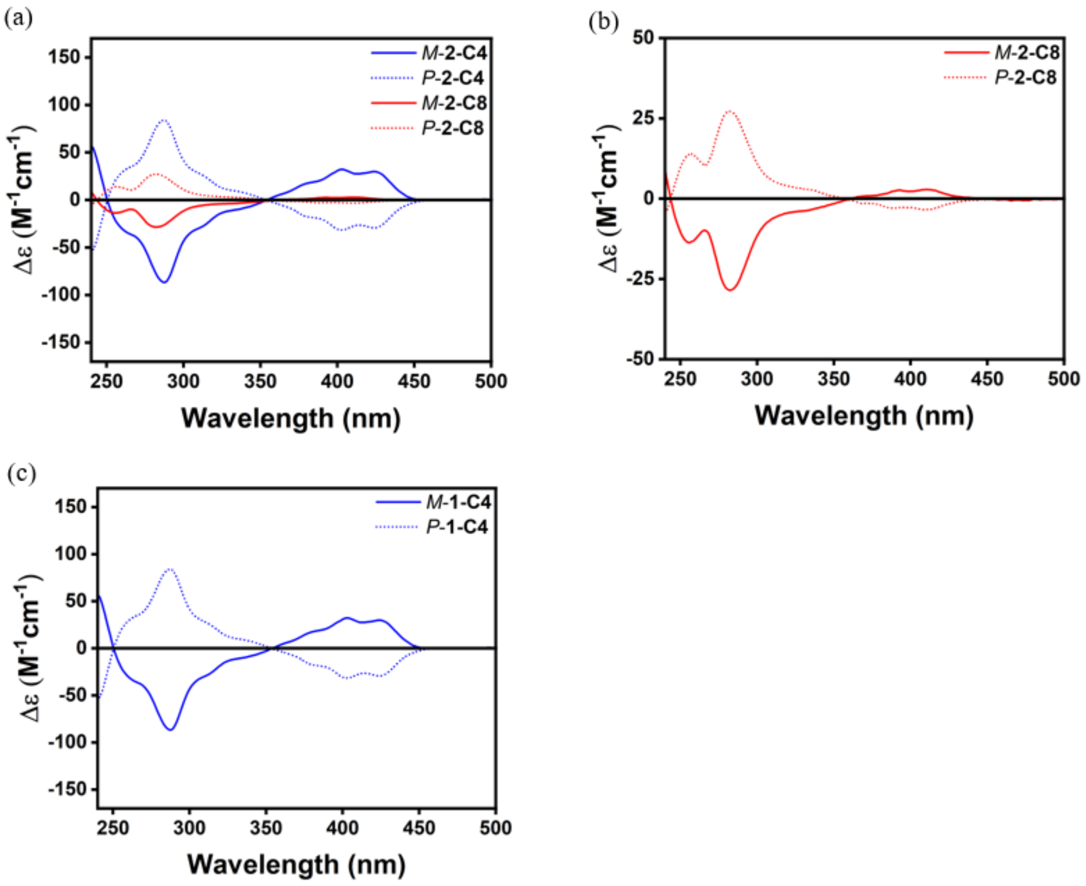

Figure S84. (a) ECD spectra of **2-Cn** (n= 4, 8) in chloroform, measured at 298 K. (b) **2-C8** (c) **2-C4**.

S5.2.3 Dissymmetry factor (g_{abs}) of 1-Cn

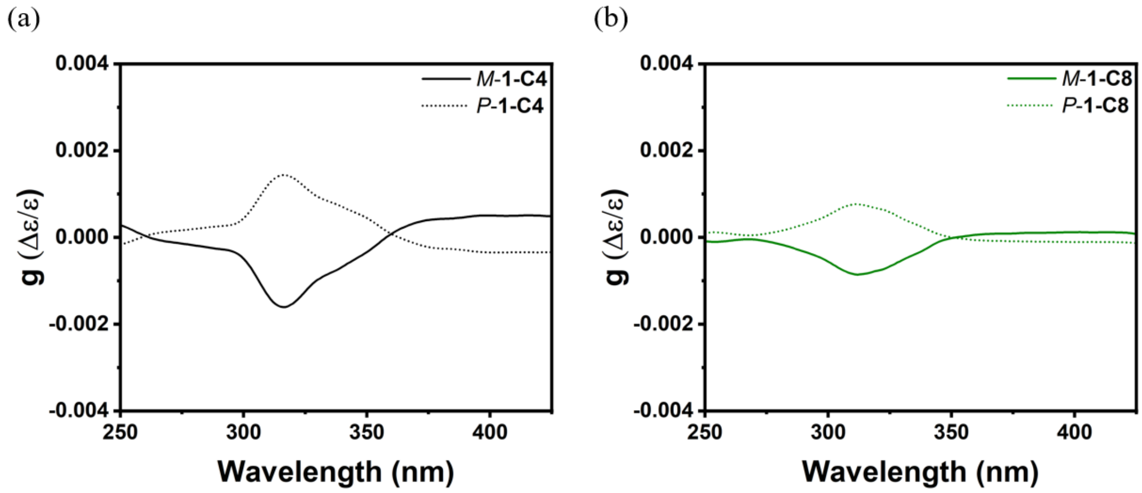

Figure S85. Experimental g_{abs} of **1-Cn** (n=4, 8) in chloroform (a) **1-C4** (b) **1-C8**.

S5.2.4 Dissymmetry factor (g_{abs}) of 2-Cn

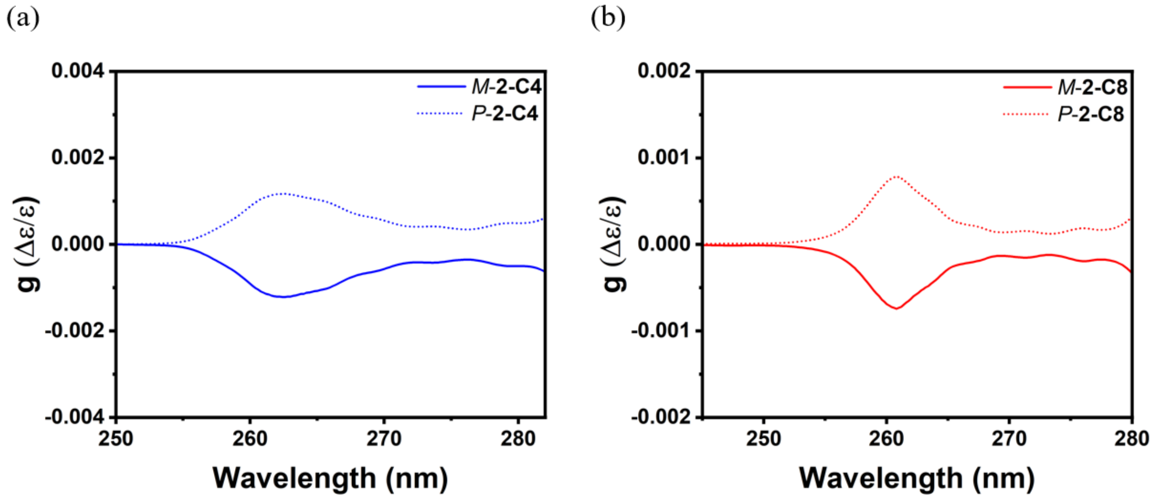

Figure S86. Experimental g_{abs} of **2-Cn** (n=4, 8) in chloroform (a) **2-C4** (b) **2-C8**.

S5.3 Fluorescence spectra

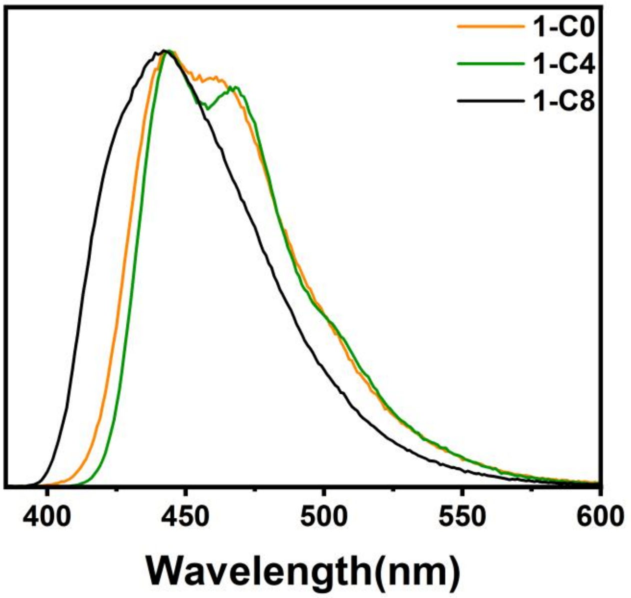

Figure S87. Fluorescence spectra of **1-Cn (n=0, 4, 8)** in hexane at 298 K.

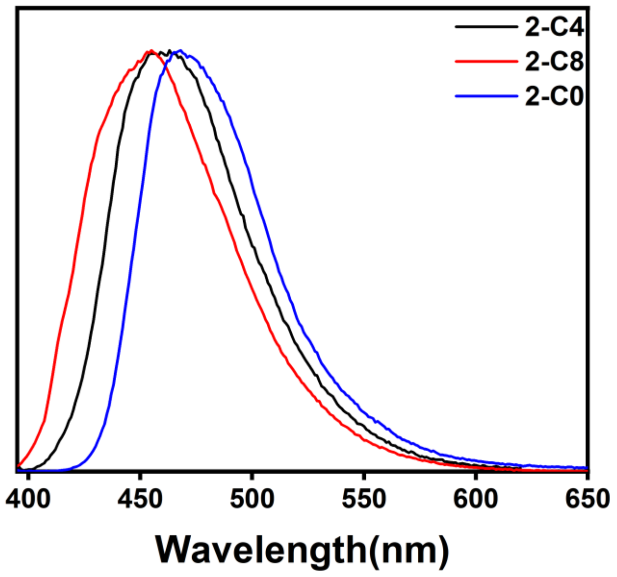

Figure S88. Fluorescence spectra **2-Cn (n=0, 4, 8)** in hexane at 298 K.

S5.4 Excitation spectra

S5.4.1 **1-Cn**

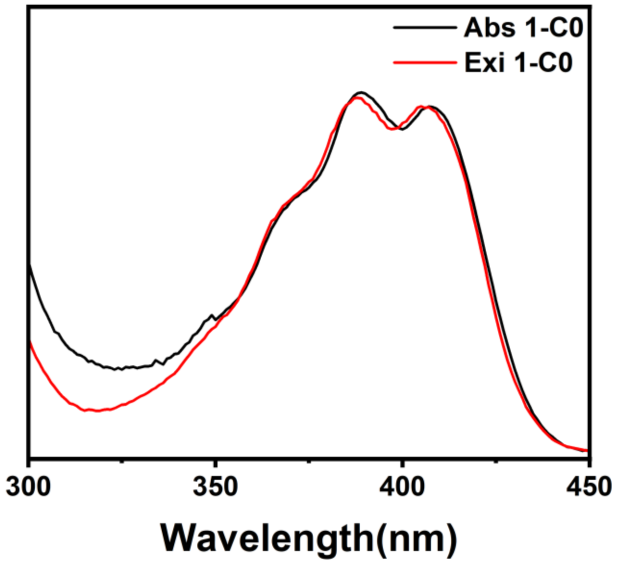

Figure S89. Excitation (red) and absorption (black) of **1-C0**.

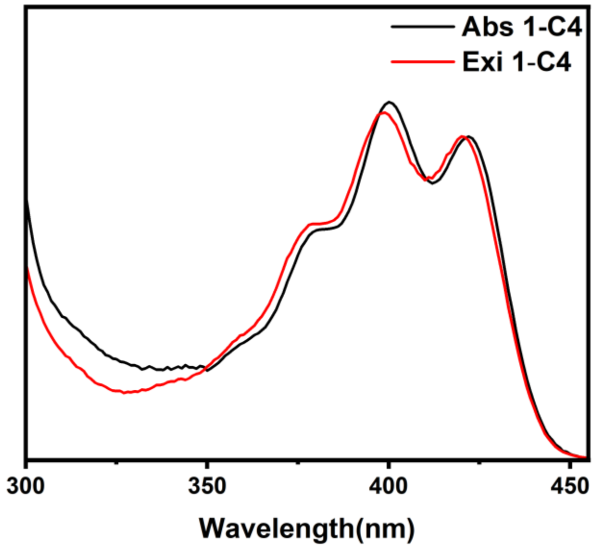

Figure S90. Excitation (red) and absorption (black) of **1-C4**.

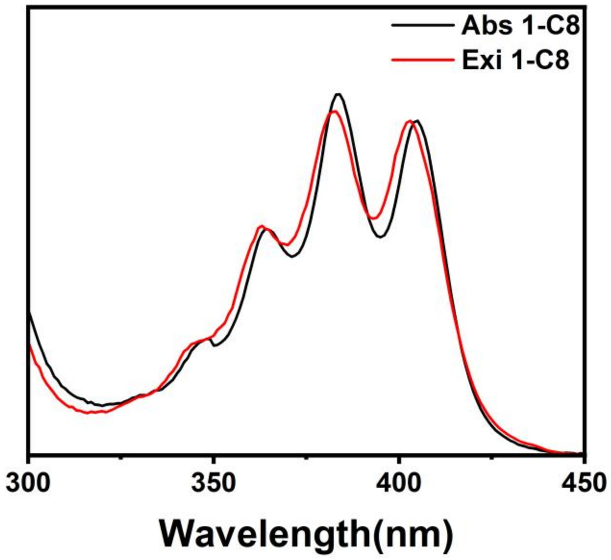

Figure S91. Excitation (red) and absorption (black) of **1-C8**.

S5.4.2 **2-Cn**

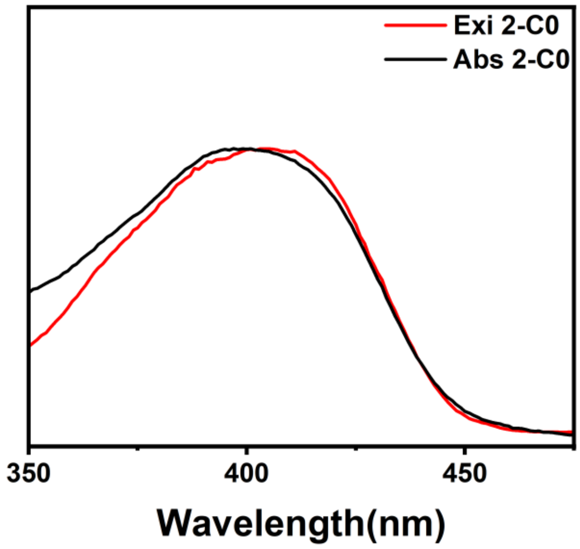

Figure S92. Excitation (red) and absorption (black) of **2-C0**.

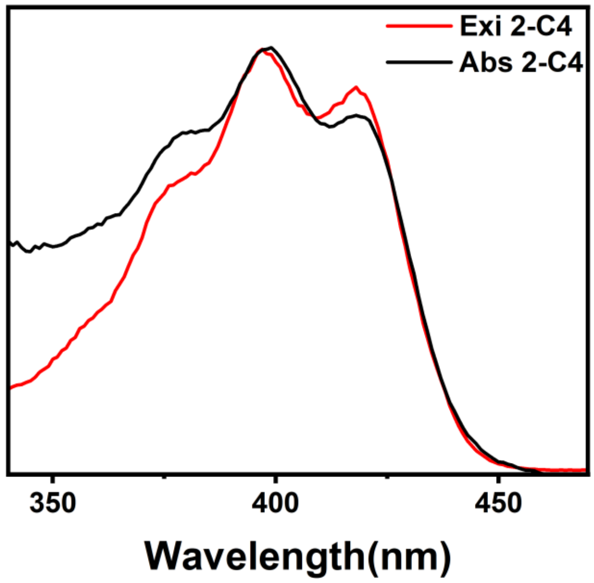

Figure S93. Excitation (red) and absorption (black) of **2-C4**.

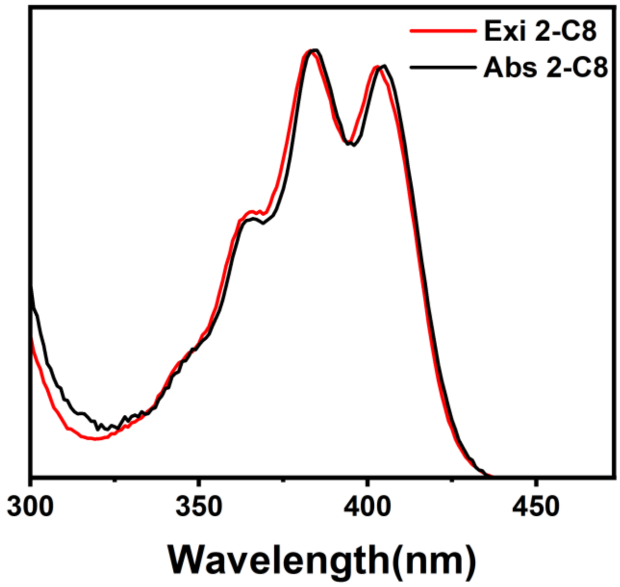

Figure S94. Excitation (red) and absorption (black) of **2-C8**.

S5.5 Fluorescence lifetime

S5.5.1 **1-Cn**

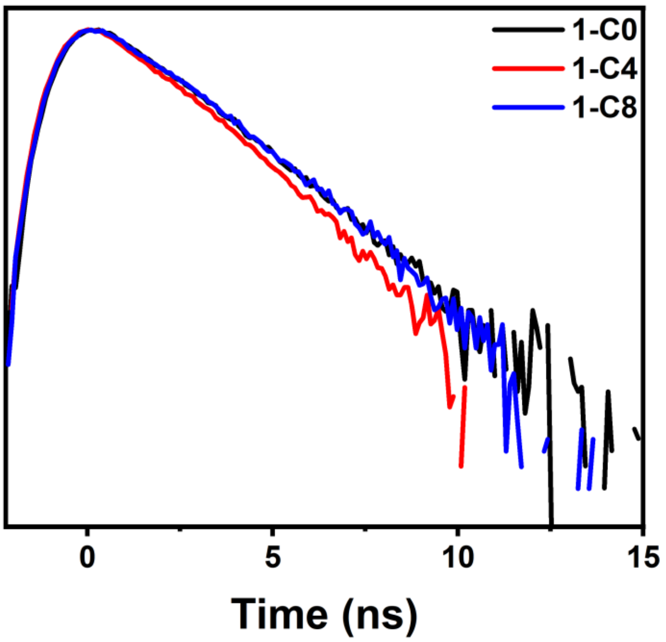

Figure S95. Fluorescence lifetimes of **1-Cn** (n=0, 4, 8) in hexane at 298K.

S5.5.2 **2-Cn**

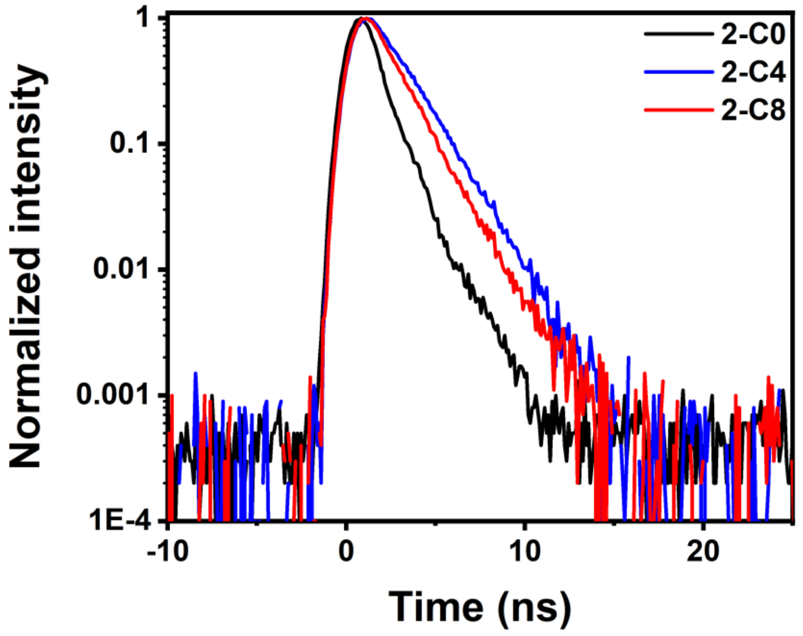

Figure S96. Fluorescence lifetime of **2-Cn** (n=0, 4, 8) in hexane at 298K.

Table 1. Fluorescence lifetime of all synthesized molecules

	Molecule	Lifetime (ns)
1	**1-C0**	1.58
2	**1-C4**	1.45
3	**1-C8**	1.62
4	**2-C0**	0.5
5	**2-C4**	1.6
6	**2-C4**	1.2

S5.6 Fluorescence quantum yields

Table 2. Fluorescence quantum yield ($\emptyset_f$) of all synthesized molecules

	Molecule	$\emptyset_f$
1	**1-C0**	10%
2	**1-C4**	11%
3	**1-C8**	16%
4	**2-C0**	3%
5	**2-C4**	10%
6	**2-C4**	30%

S6 Single-crystal X-ray diffraction crystallography (SCXRD).

S6.1 X-ray structures

S6.1.1 **2-C4**

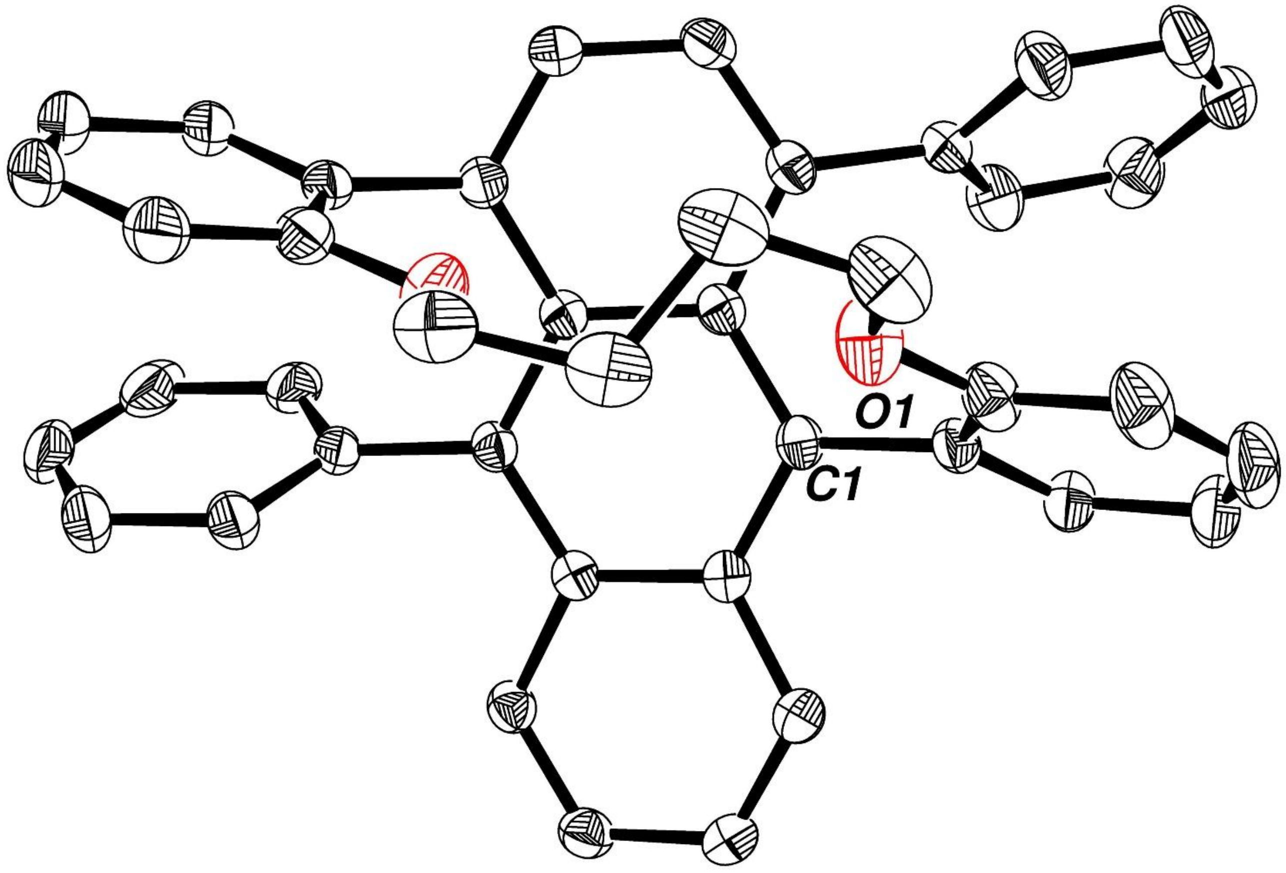

Figure S97. Top view of the ORTEP ellipsoid representation of **2-C4**, 50% probability.

S6.1.2 **1-C4**

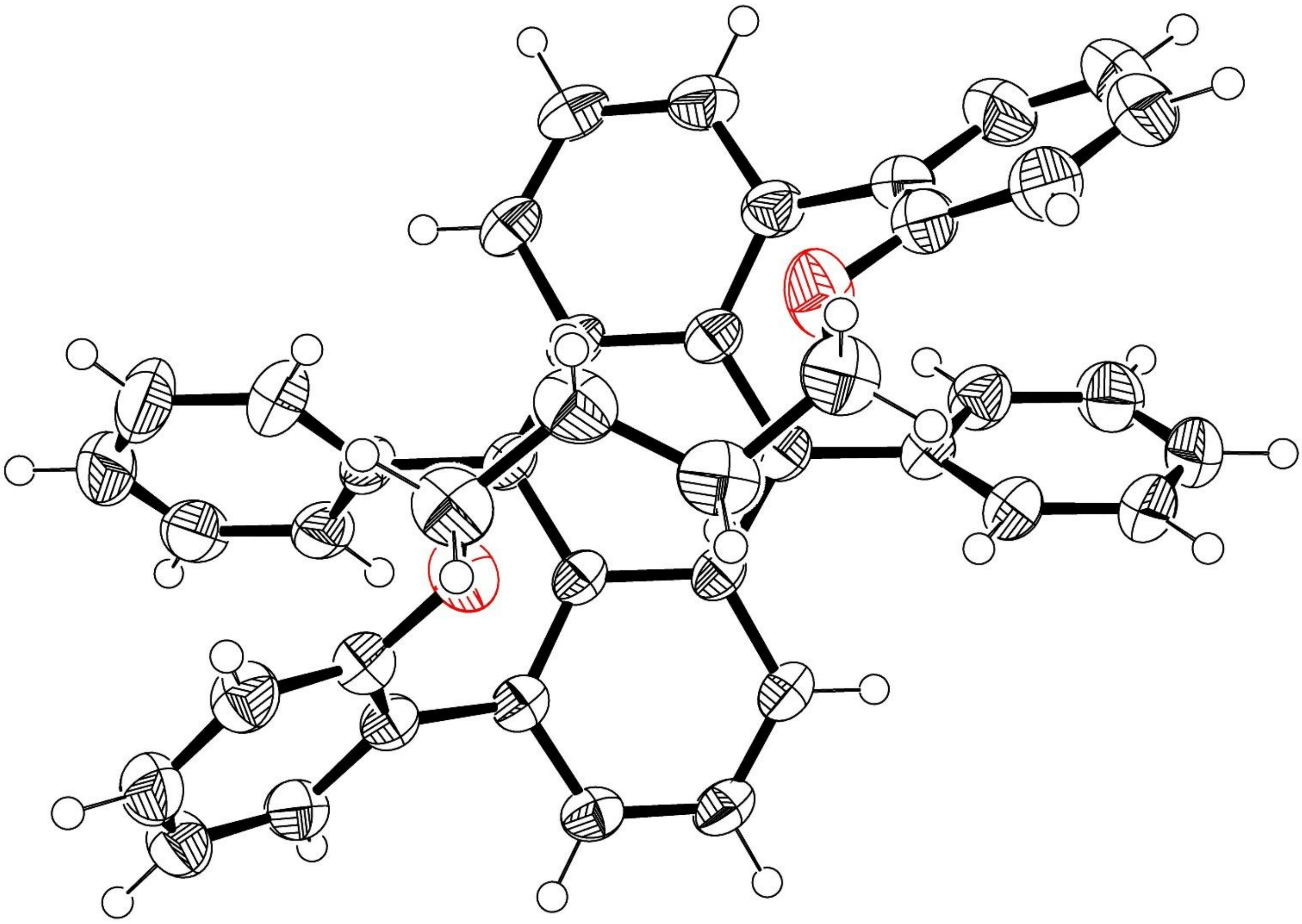

Figure S98. Top view of the ORTEP ellipsoid representation of **1-C4**, 50% probability.

S6.1.3 **2-C8**

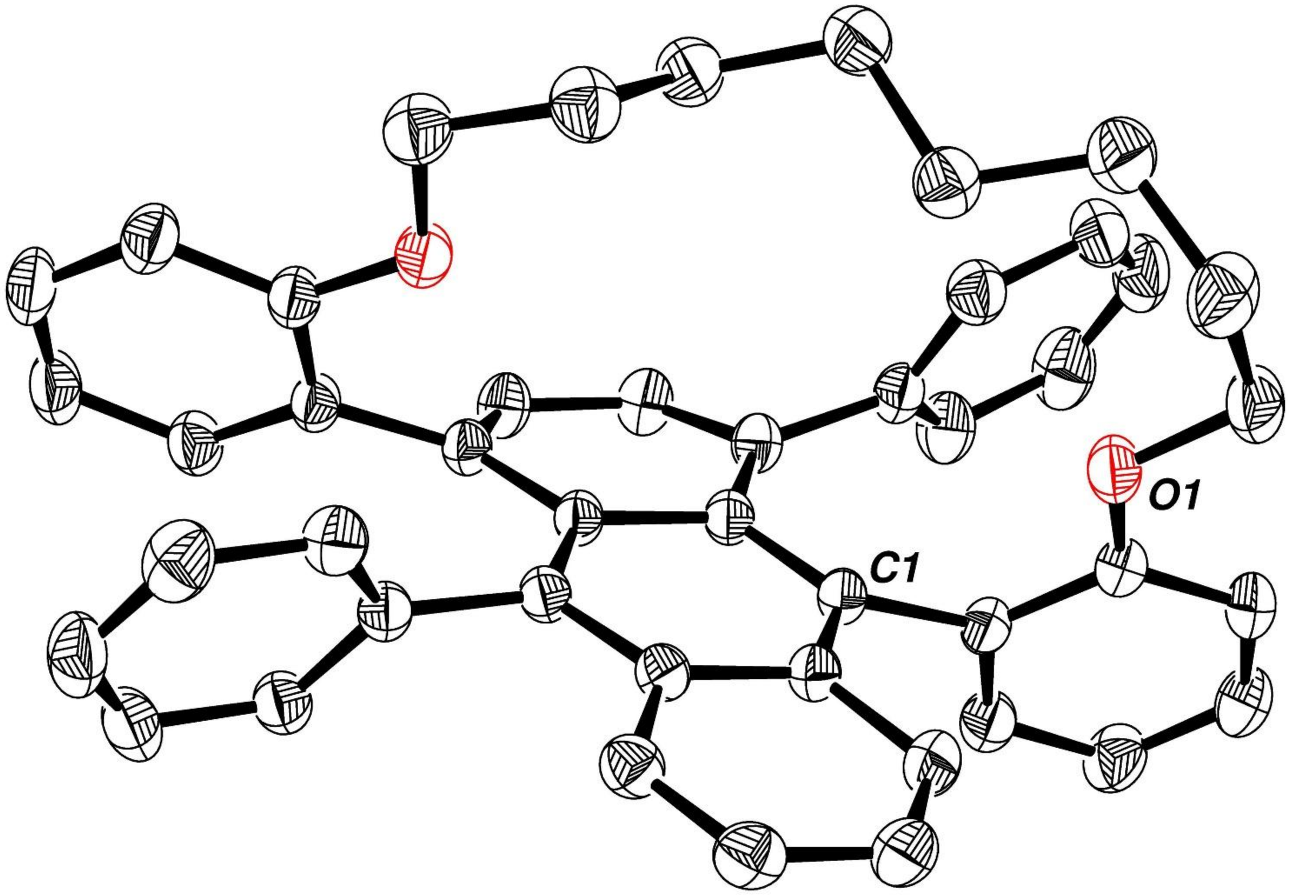

Figure S99. Top view of the ORTEP ellipsoid representation of **2-C8**, 50% probability.

S6.1.4 **1-C8**

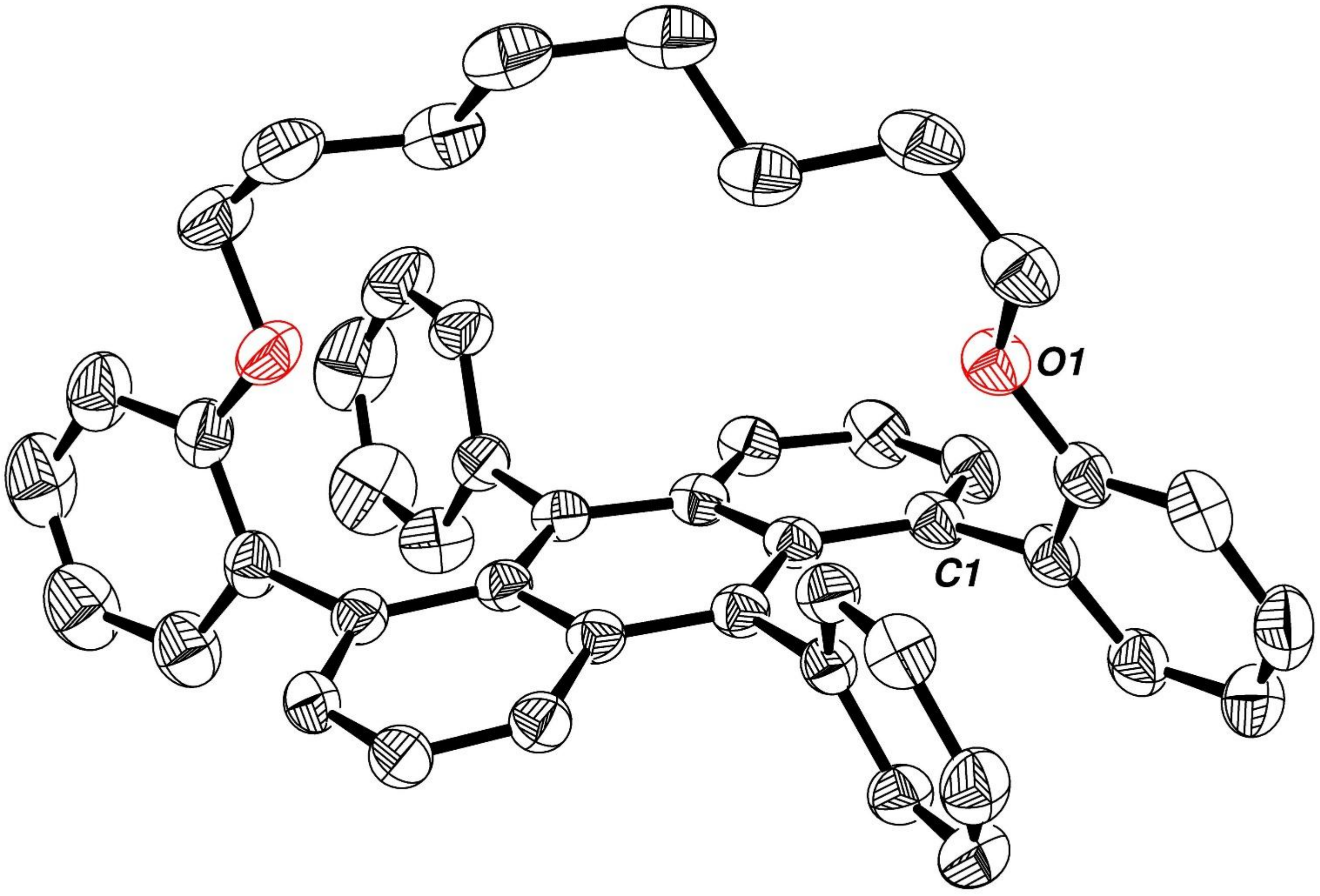

Figure S100. Top view of the ORTEP ellipsoid representation of **1-C8**, 50% probability.

S6.1.5 **4-C8**

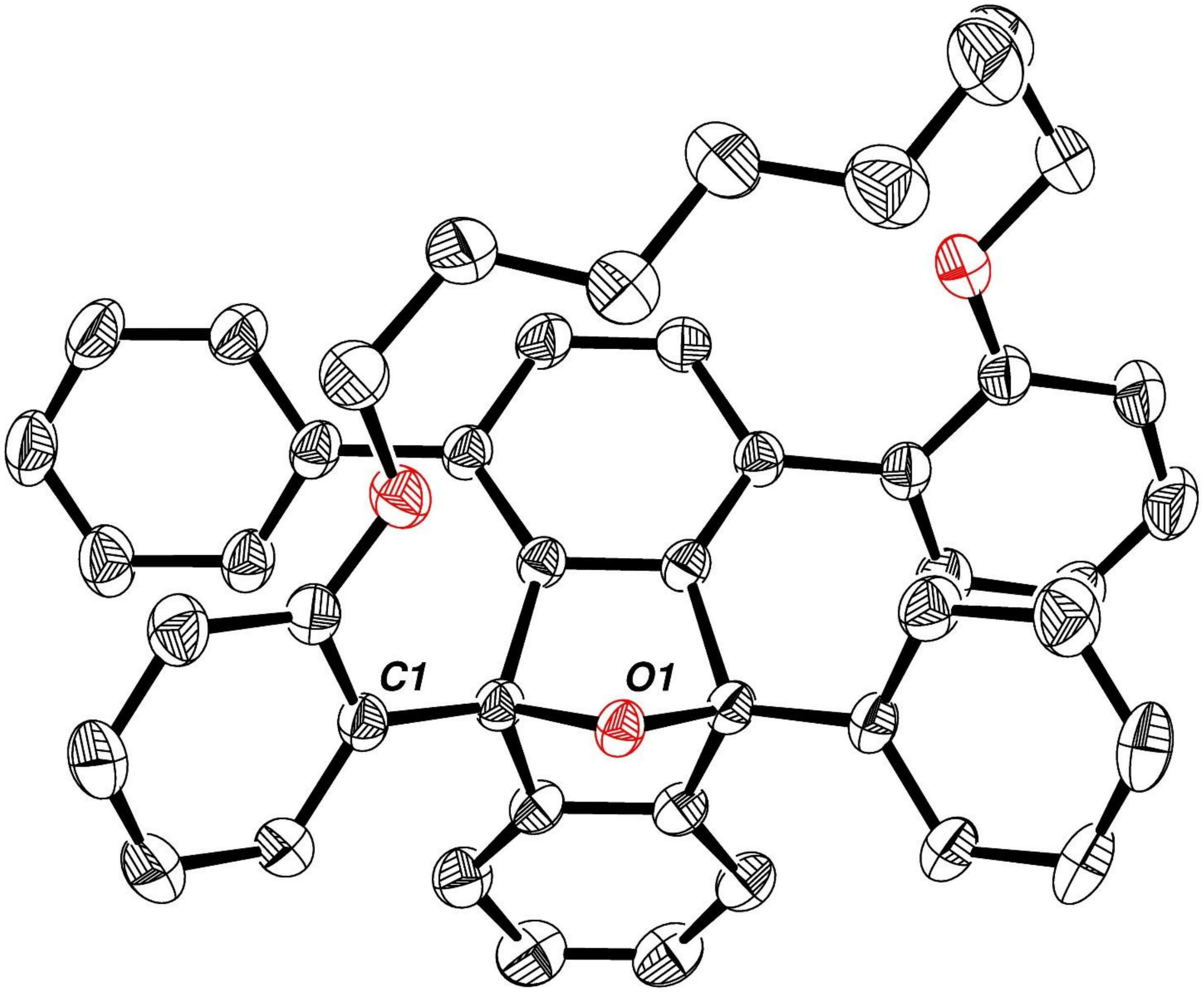

Figure S101. Top view of the ORTEP ellipsoid representation of **4-C8**, 50% probability.

S6.1.6 **2-C0**

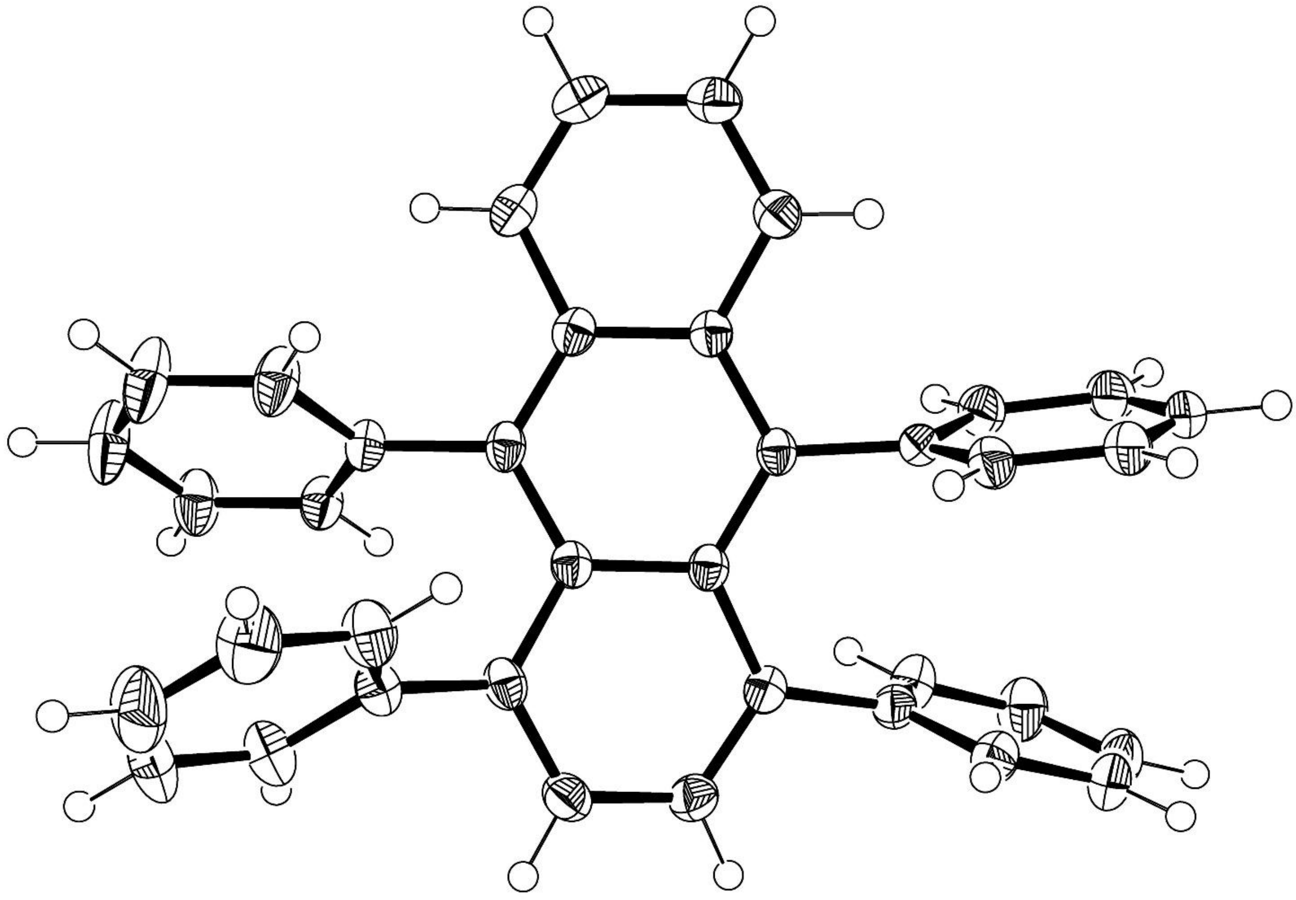

Figure S102. Top view of the ORTEP ellipsoid representation of **2-C0**, 50% probability.

S6.2 Crystals data

S6.2.1 **2-C4**

Table 3. Crystal data and structure refinement for **2-C4.**

Identification code	OriGi99
CCDC No	2471207
Empirical formula	$C_{42}H_{32}O_2$
Formula weight	568.67
Temperature/K	149.99(10)
Crystal system	triclinic
Space group	P-1
a/Å	13.8023(2)
b/Å	14.0475(2)
c/Å	17.1368(2)
α/°	90.7610(10)
β/°	101.3620(10)
γ/°	110.6410(10)
Volume/Å^3	3035.97(7)
Z	4
ρ_{calc}g/cm^3	1.244
μ/mm^{-1}	0.075
F(000)	1200.0
Crystal size/mm^3	0.47 × 0.13 × 0.11
Radiation	Mo Kα (λ = 0.71073)
2Θ range for data collection/°	3.6 to 55.998
Index ranges	-18 ≤ h ≤ 18, -18 ≤ k ≤ 18, -22 ≤ l ≤ 22
Reflections collected	100307
Independent reflections	14654 [R_{int} = 0.0529, R_{sigma} = 0.0767]
Data/restraints/parameters	14654/0/793
Goodness-of-fit on F^2	1.058
Final R indexes [I>=2σ (I)]	R_1 = 0.0426, wR_2 = 0.1162
Final R indexes [all data]	R_1 = 0.0564, wR_2 = 0.1208
Largest diff. peak/hole / e Å^{-3}	0.34/-0.21

S6.2.2 **1-C4**

Table 4. Crystal data and structure refinement for **1-C4.**

CCDC No	2471208
Empirical formula	$C_{42}H_{32}O_2$
Formula weight	568.67
Temperature/K	230.15
Crystal system	monoclinic
Space group	$P2_1/c$
a/Å	15.0135(3)
b/Å	14.4952(2)
c/Å	14.1389(3)
α/°	90
β/°	102.321(2)
γ/°	90
Volume/Å^3	3006.1(1)
Z	4
ρ_{calc}g/cm^3	1.257
μ/mm^{-1}	0.076
F(000)	1200.0
Crystal size/mm^3	0.34 × 0.29 × 0.21
Radiation	MoKα (λ = 0.71073)
2Θ range for data collection/°	3.95 to 64.664
Index ranges	-21 ≤ h ≤ 21, -21 ≤ k ≤ 20, -21 ≤ l ≤ 21
Reflections collected	61415
Independent reflections	9474 [R_{int} = 0.0349, R_{sigma} = 0.0249]
Data/restraints/parameters	9474/0/397
Goodness-of-fit on F^2	1.038
Final R indexes [I>=2σ (I)]	R_1 = 0.0454, wR_2 = 0.1194
Final R indexes [all data]	R_1 = 0.0719, wR_2 = 0.1351
Largest diff. peak/hole / e Å^{-3}	0.25/-0.27

S6.2.3 **2-C8**

Table 5. Crystal data and structure refinement for **2-C8.**

CCDC No	2471209
Empirical formula	$C_{46}H_{40}O_2$
Formula weight	624.78
Temperature/K	210.0(1)
Crystal system	monoclinic
Space group	$P2_1/c$
a/Å	13.7941(3)
b/Å	10.4938(2)
c/Å	23.5497(6)
α/°	90
β/°	99.102(2)
γ/°	90
Volume/$Å^3$	3365.96(13)
Z	4
$\rho_{calc}g/cm^3$	1.233
μ/mm^{-1}	0.074
F(000)	1328.0
Crystal size/mm^3	0.52 × 0.41 × 0.27
Radiation	Mo Kα (λ = 0.71073)
2Θ range for data collection/°	4.258 to 64.578
Index ranges	$-18 \leq h \leq 20$, $-15 \leq k \leq 15$, $-32 \leq l \leq 31$
Reflections collected	46652
Independent reflections	10286 [R_{int} = 0.0277, R_{sigma} = 0.0266]
Data/restraints/parameters	10286/0/434
Goodness-of-fit on F^2	1.061
Final R indexes [I>=2σ (I)]	R_1 = 0.0459, wR_2 = 0.1196
Final R indexes [all data]	R_1 = 0.0670, wR_2 = 0.1313
Largest diff. peak/hole / e $Å^{-3}$	0.31/-0.19

S6.2.4 **1-C8**

Table 6. Crystal data and structure refinement for **1-C8.**

CCDC No	2471206
Empirical formula	$C_{49}H_{47}O_2$
Formula weight	667.86
Temperature/K	200.0(1)
Crystal system	monoclinic
Space group	I2/a
a/Å	11.9497(4)
b/Å	22.5559(7)
c/Å	27.8715(8)
α/°	90
β/°	99.603(3)
γ/°	90
Volume/Å^3	7407.1(4)
Z	8
ρ_{calc}g/cm^3	1.198
μ/mm^{-1}	0.071
F(000)	2856.0
Crystal size/mm^3	0.36 × 0.09 × 0.09
Radiation	Mo Kα (λ = 0.71073)
2Θ range for data collection/°	4.672 to 62.164
Index ranges	-16 ≤ h ≤ 16, -26 ≤ k ≤ 30, -33 ≤ l ≤ 39
Reflections collected	26643
Independent reflections	9396 [R_{int} = 0.0290, R_{sigma} = 0.0362]
Data/restraints/parameters	9396/0/461
Goodness-of-fit on F^2	1.023
Final R indexes [I>=2σ (I)]	R_1 = 0.0568, wR_2 = 0.1422
Final R indexes [all data]	R_1 = 0.0866, wR_2 = 0.1569
Largest diff. peak/hole / e Å^{-3}	0.26/-0.36

S6.2.5 **4-C8**

Table 7. Crystal data and structure refinement for **4-C8**

CCDC No	2471210
Empirical formula	$C_{46}H_{40}O_3$
Formula weight	640.78
Temperature/K	210.0(1)
Crystal system	orthorhombic
Space group	$Pna2_1$
a/Å	15.7755(2)
b/Å	10.8416(2)
c/Å	20.2833(3)
α/°	90
β/°	90
γ/°	90
Volume/Å^3	3469.09(9)
Z	4
ρ_{calc}g/cm^3	1.227
μ/mm^{-1}	0.075
F(000)	1360.0
Crystal size/mm^3	0.29 × 0.24 × 0.18
Radiation	Mo Kα (λ = 0.71073)
2Θ range for data collection/°	4.016 to 64.79
Index ranges	$-23 \le h \le 21$, $-16 \le k \le 16$, $-28 \le l \le 29$
Reflections collected	72983
Independent reflections	10924 [R_{int} = 0.0434, R_{sigma} = 0.0295]
Data/restraints/parameters	10924/1/442
Goodness-of-fit on F^2	1.042
Final R indexes [I>=2σ (I)]	R_1 = 0.0428, wR_2 = 0.1151
Final R indexes [all data]	R_1 = 0.0524, wR_2 = 0.1211
Largest diff. peak/hole / e Å^{-3}	0.37/-0.20
Flack parameter	-0.1(3)

S6.2.6 **2-C0**

Table 8. Crystal data and structure refinement for 2-C0.

CCDC No	2471211
Empirical formula	$C_{38}H_{26}$
Formula weight	482.59
Temperature/K	180.0(1)
Crystal system	monoclinic
Space group	$P2_1/n$
a/Å	7.6053(2)
b/Å	12.4687(4)
c/Å	28.1521(8)
α/°	90
β/°	96.141(3)
γ/°	90
Volume/Å^3	2654.3(1)
Z	4
ρ_{calc}g/cm^3	1.208
μ/mm^{-1}	0.068
F(000)	1016.0
Crystal size/mm^3	0.32 × 0.08 × 0.06
Radiation	Mo Kα (λ = 0.71073)
2Θ range for data collection/°	4.376 to 64.342
Index ranges	-9 ≤ h ≤ 11, -16 ≤ k ≤ 18, -39 ≤ l ≤ 40
Reflections collected	20806
Independent reflections	7792 [R_{int} = 0.0355, R_{sigma} = 0.0471]
Data/restraints/parameters	7792/0/343
Goodness-of-fit on F^2	1.053
Final R indexes [I>=2σ (I)]	R_1 = 0.0488, wR_2 = 0.1218
Final R indexes [all data]	R_1 = 0.0721, wR_2 = 0.1329
Largest diff. peak/hole / e Å^{-3}	0.27/-0.24

S7 Computational Details.

Geometry optimizations and transition states were performed using density functional theory (DFT).[3] The functional we have chosen is B3LYP with the 6-311G(d) basis set and dispersion correction (GD3).[4] No symmetry restrictions were applied in any of the optimizations. The optimal geometries for all structures were confirmed as minima by frequency calculations. No negative frequencies were found for any minimum points presented in this work. All calculations were performed using Gaussian 16. The UV-vis and CD spectral transition of the molecules, time dependent (TD)-DFT calculations were performed using the CAM-B3LYP functional with the 6-31G(d) basis set. To identify the lowest-energy conformers, we employed GMMX using the MMFF94 force field, with an energy window of up to 5 kcal/mol. The 16 lowest-energy conformers obtained from GMMX were further optimized using DFT at the B3LYP/6-311G(d)-GD3 level of theory.

Relative energies of conformers identified by GMMX after DFT optimization. One key parameter distinguishing the conformers is the dihedral angle of the anthracene moiety, termed as φ between A–B–C–D (see Figure 1 in the main text). The conformational space is represented as a function of this dihedral angle in the following graphs:

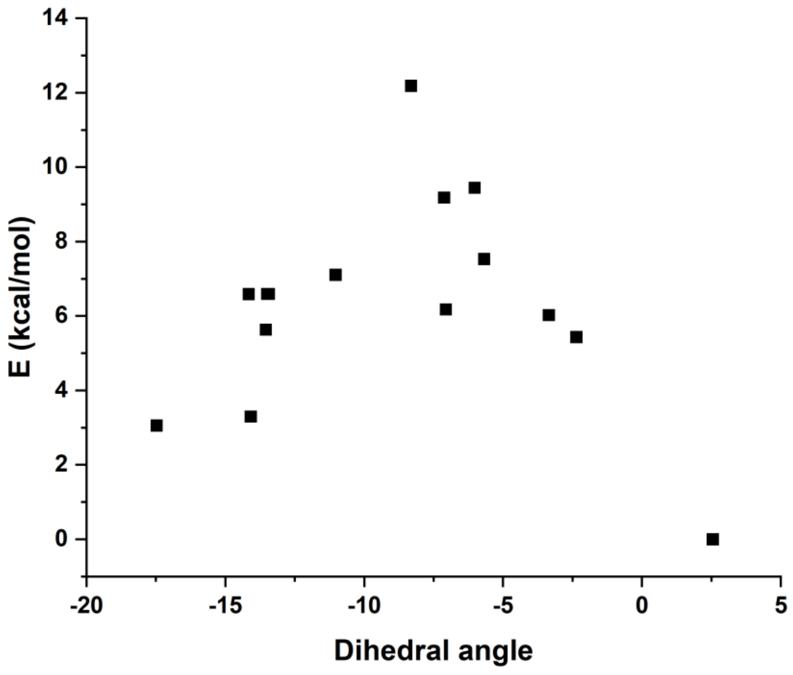

Figure S103. Plot of energy versus dihedral angle parameter distinguishing the conformers of **1-C8**. The y-axis represents the relative energy vs. the most stable conformer, and the x-axis corresponds to the dihedral (φ).

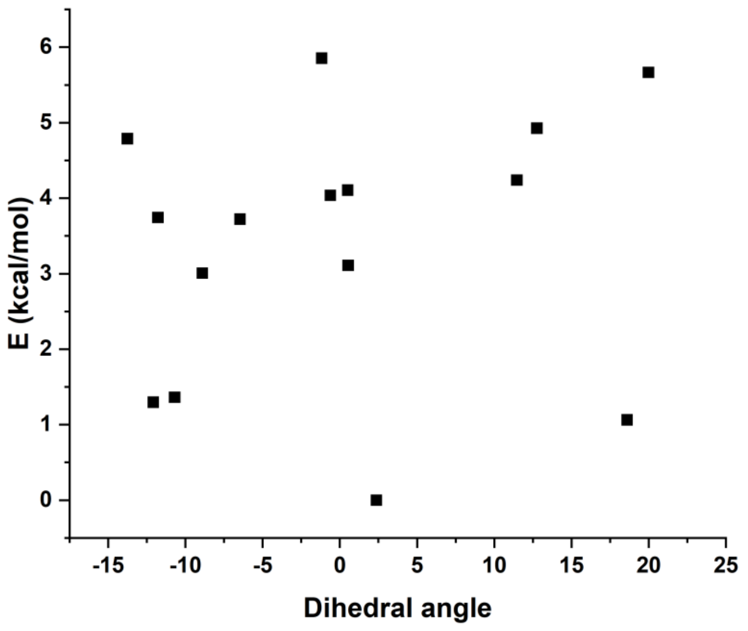

Figure S104. Plot of energy versus dihedral angle parameter distinguishing the conformers of **2-C8**. The y-axis represents the relative energy vs. the most stable conformer, and the x-axis corresponds to the dihedral (φ).

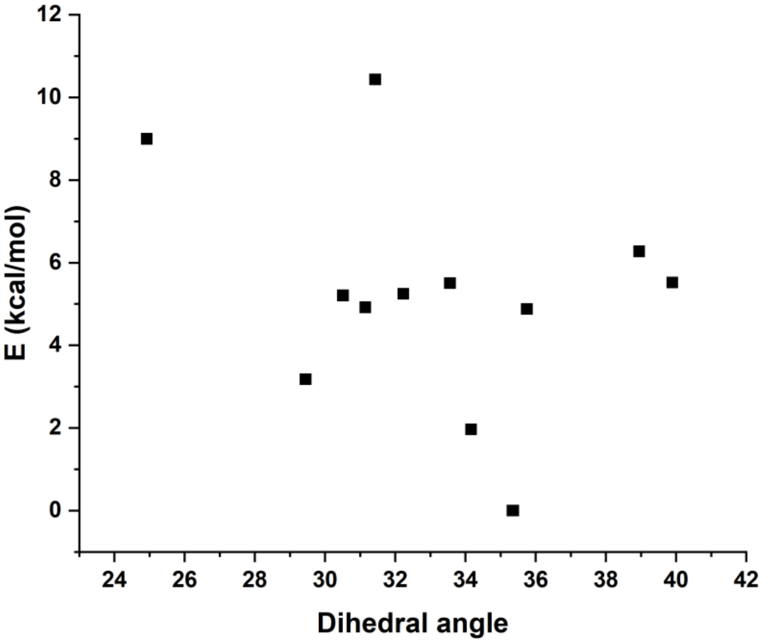

Figure S105. Plot of energy versus dihedral angle parameter distinguishing the conformers of **1-C4**. The y-axis represents the relative energy vs. the most stable conformer, and the x-axis corresponds to the dihedral (φ).

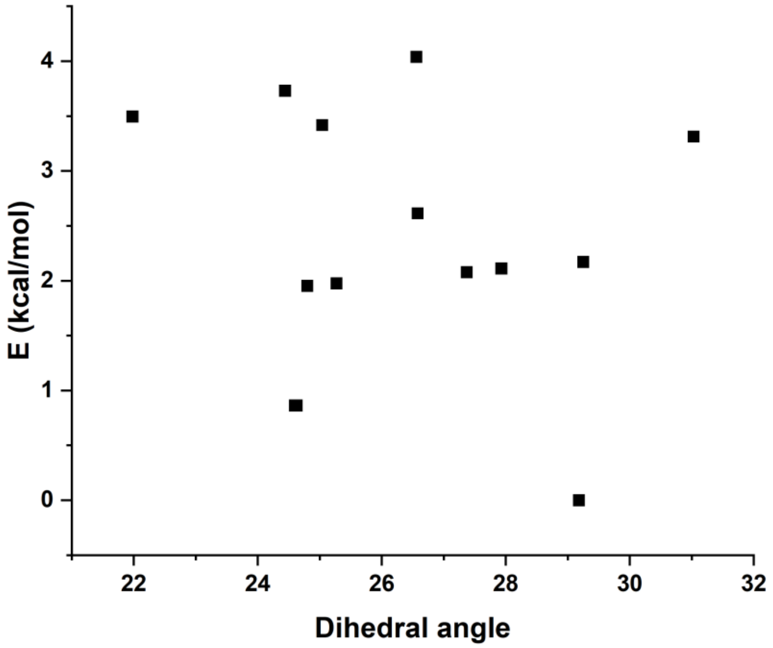

Figure S106. Plot of energy versus dihedral angle parameter distinguishing the conformers of **2-C4**. The y-axis represents the relative energy vs. the most stable conformer, and the x-axis corresponds to the dihedral (φ).

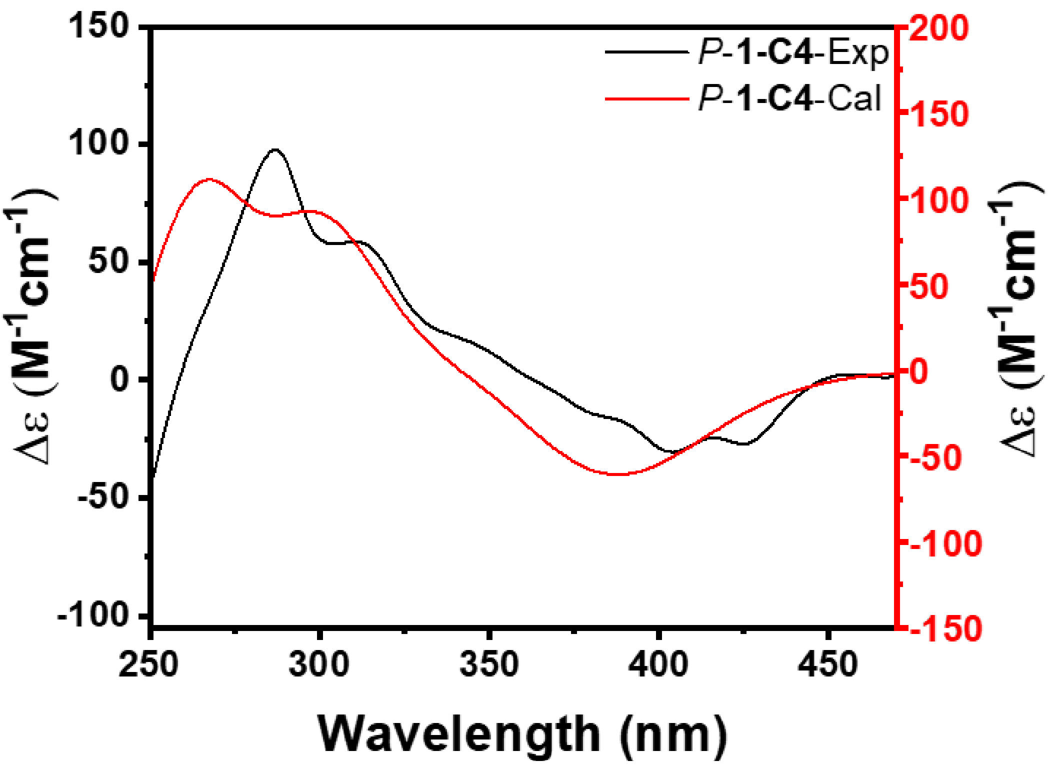

Figure S107. Black trace: experimental ECD spectra in chloroform. Red trace: calculated (TD-DFT-6-31G(d)-CAMB3LYP) spectra of *P*-**1**-**C4**.

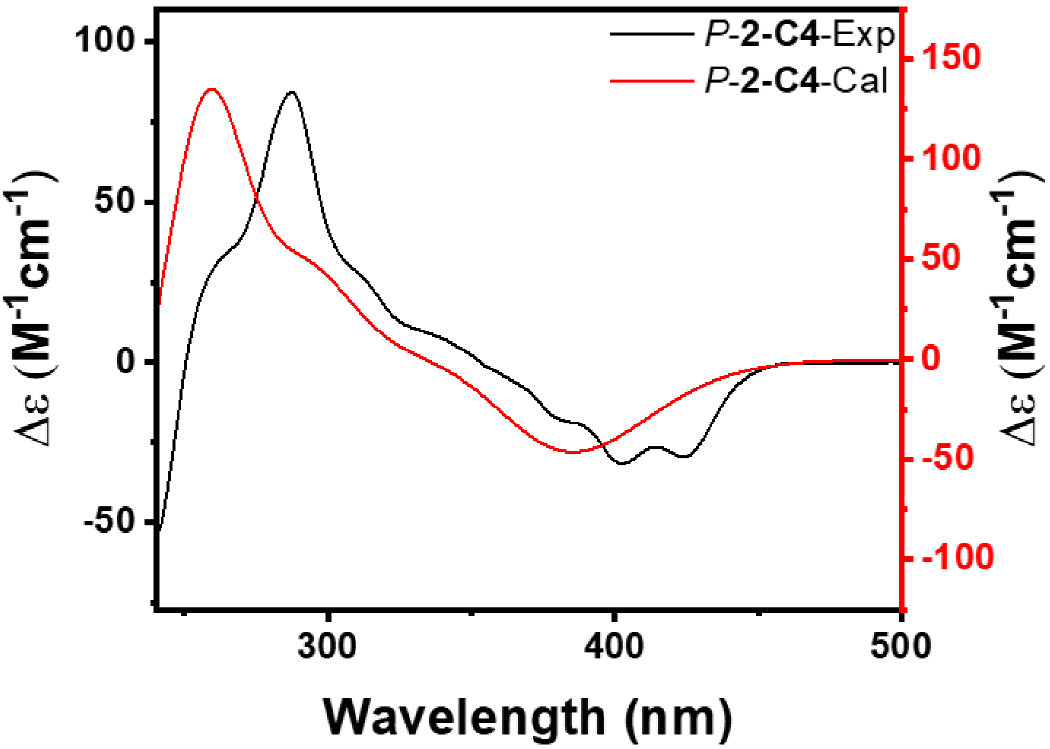

Figure S108. Black trace: experimental ECD spectra in chloroform. Red trace: calculated (TD-DFT-6-31G(d)-CAMB3LYP) spectra of *P*-**2**-**C4**.

S8 References

(1) Smet, M.; Shukla, R.; Fülöp, L.; Dehaen, W. A General Synthesis of Disubstituted Rubicenes. *Eur. J. Org. Chem.* **1998**, *1998* (12), 2769–2773. https://doi.org/10.1002/(SICI)1099-0690(199812)1998:12<2769::AID-EJOC2769>3.0.CO;2-7.

(2) Agrawal, A. R.; Shioukhi, I.; Deree, Y.; Bogoslavsky, B.; Shalev, O.; Hoffman, R.; Gidron, O. Controlling the Helicity and Handedness of Polyaromatics with Isobenzofuranophane. *Angew. Chem. Int. Ed.* **2025**, *n/a* (n/a), e202510423. https://doi.org/10.1002/anie.202510423.

(3) Calais, J.-L. Density-Functional Theory of Atoms and Molecules. R.G. Parr and W. Yang, Oxford University Press, New York, Oxford, 1989. IX + 333 Pp. Price £45.00. *Int. J. Quantum Chem.* **1993**, *47* (1), 101–101. https://doi.org/10.1002/qua.560470107.

(4) Lee, C.; Yang, W.; Parr, R. G. Development of the Colle-Salvetti Correlation-Energy Formula into a Functional of the Electron Density. *Phys. Rev. B* **1988**, *37* (2), 785–789. https://doi.org/10.1103/PhysRevB.37.785.
